# Swimming patterns of a multi-mode bacterial swimmer in fluid shear flow

**DOI:** 10.1016/j.bpj.2026.05.032

**Published:** 2026-05-30

**Authors:** Valeriia Fast, Agniva Datta, Jeungeun Park, Robert Großmann, Veronika Pfeifer, Yongsam Kim, Wanho Lee, Sookkyung Lim, Carsten Beta

**Affiliations:** 1 Institute of Physics and Astronomy, University of Potsdam, 14476 Potsdam, Germany; 2 Department of Mathematics, State University of New York at New Paltz, New Paltz, NY 12561, USA; 3 Department of Mathematics, Chung-Ang University, Seoul 06974, South Korea; 4 National Institute for Mathematical Sciences, Daejeon 34047, South Korea; 5 Department of Mathematical Sciences, University of Cincinnati, Cincinnati, OH 45221, USA; 6 Nano Life Science Institute (WPI-NanoLSI), Kanazawa University, Kanazawa 920-1192, Japan

## Abstract

Bacterial swimming is well characterized in uniform liquids at rest. The natural habitat of bacterial swimmers, however, is often dominated by moving fluids and interfaces, resulting in shear flows that may strongly alter bacterial navigation strategies. Here, we study how fluid shear flow affects the swimming motility of the soil bacterium *Pseudomonas putida*, a bacterial swimmer that moves in a versatile pattern composed of three different swimming modes, where the flagella may push, pull, or wrap around the cell body (multi-mode swimmer). We introduce a computer automated cell tracking and swimming mode detection tool to show that shear-induced alignment depends on the swimming mode, while motility and proximity to surfaces counteract the alignment effect. Moreover, filament wrapping becomes less efficient with increasing shear stress. Numerical simulations of realistic swimmer geometries complement our experimental results, providing more detailed mechanistic insights into movement patterns of bacterial swimmers in a shear flow.

## Significance

When colonizing plant roots in the soil or during spreading of infections in the human body, bacteria propel themselves through moving fluids. This work explores how fluid shear flow influences the swimming pattern of *Pseudomonas putida*, a soil bacterium that moves in three alternating swimming modes, where flagella push, pull, or wrap around the cell body. Using dual-color fluorescence microscopy together with semi-automated image analysis, we reveal that shear-induced alignment depends on the swimming mode. Moreover, increased shear stress stabilizes the pulling mode by reducing the efficiency of filament wrapping. Numerical simulations support our experimental findings, providing insights into bacterial movement patterns under flow conditions and emphasizing how distinct types of swimmers differ in their response to shear flow.

## Introduction

The locomotion of biological microswimmers in complex environments is central to many medical functions, such as the spreading of infections^1^^,^^2^ or sperm navigation inside the female reproductive tract.^3^^,^^4^ For bacteria, one of the largest classes of biological microswimmers, studies in uniform liquid environments have shown that they control their direction of locomotion by changing the mode of operation of their helical flagella. *Escherichia coli* (*E. coli*), the most widely studied example, carries flagella distributed all over its cell body (peritrichous flagellation) and moves in a run-and-tumble pattern.^5^ Here, counterclockwise (CCW) rotation of the flagella results in the formation of a coherent bundle that pushes the cell body forward (run). Persistent run episodes are interrupted by erratic turn events (tumbles) that are induced by one or several motors switching to clockwise (CW) rotation, driving the coherent bundle apart and resulting in a random reorientation of the cell body.^6^

Apart from the well-studied run-and-tumble motility of peritrichously flagellated *E. coli*, more complex swimming patterns have been reported for bacteria that exhibit other flagellation architectures.^7^ While *E. coli* always swims as a pusher, other species may exhibit combinations of different run modes (multi-mode swimming), such as in the run-reverse-flick pattern of monotrichously flagellated species.^8^ Here, we focus on the soil bacterium *Pseudomonas putida* (*P. putida*) as an example of a multi-mode bacterial swimmer that has been intensely studied both in open uniform liquid^9^ and in confined and complex environmental geometries.^10^^,^^11^^,^^12^^,^^13^ *P. putida* carries a tuft of several helical flagella located at one cell pole (lophotrichous flagellation).^14^ It may push itself forward by CCW rotation of its flagellar bundle, while CW operation of the flagellar motor pulls the cell body in the opposite direction. Additionally, *P. putida* can wrap its bundle of flagella around the cell body to move in a screw thread fashion (wrapped mode),^15^ a swimming mode that has also been reported for other polarly flagellated species.^16^^,^^17^^,^^18^ Wrapped mode formation is promoted by increasing the viscosity of the surrounding medium^16^^,^^19^ and plays a key role for chemotaxis of *P. putida* in nutrient gradients.^20^ Nevertheless, the functional relevance of filament wrapping and the general benefits of switching between different swimming modes are not fully understood and remain a matter of current debate.^21^

In many natural habitats of bacterial swimmers, fluid flows are commonly observed, such as blood flow in the vascular system or groundwater flows in a granular soil environment.^1^^,^^22^^,^^23^ Close to surfaces, the velocity gradients of the resulting shear flows will affect the direction of bacterial locomotion. According to the pioneering work by Jeffery, a passive elongated object in a linear shear flow will perform periodic revolutions, following so-called *Jeffery’s orbits.*^24^ The study of shear flow effects on particle dynamics was later extended by Bretherton and others.^25^^,^^26^ For active particles in a Poiseuille flow, two types of trajectory patterns, “tumbling” and “swinging,” defined by initial conditions and particle shape, were observed in experiments^27^ and described theoretically.^28^ Later, also rheotaxis in fluid shear flows, the directed drift of nonmotile and motile chiral objects across streamlines, was reported^29^^,^^30^^,^^31^^,^^32^^,^^33^ and complemented by mathematical models.^34^^,^^35^ For elongated swimmers with chiral filaments, the local shear-induced alignment with the flow competes with the chirality-induced side drift.^30^^,^^35^ Also close to a surface, the combination of shear flow and other hydrodynamic effects leads to the upstream motion of microswimmers.^30^^,^^31^^,^^36^ All of the above demonstrates that complex environmental flow conditions may strongly affect the spreading of a population of bacterial microswimmers.

In this work, we examine how the swimming behavior of a multi-mode bacterial swimmer is altered under flow conditions. We focus on *P. putida* and report experimental and theoretical results on the swimming pattern of this multi-mode swimmer in bulk and near a solid surface under local shear flow. To examine the runs in each swimming mode separately, dual-color fluorescent microscopy recordings were combined with semi-automatic tracking and swimming mode detection based on a dedicated software tool. On the theoretical side, these results were complemented with mathematical modeling of a realistic swimmer geometry. The model relied on Kirchhoff rod theory to describe the flagellar filaments and their hooks, rigid body dynamics to capture the motion of the cell body, and a generalization of the regularized Stokeslet method to account for their hydrodynamic interactions.^37^^,^^38^^,^^39^ In this way, we can evaluate the pros and cons of each swimming configuration. Furthermore, our study will provide the basis for future predictions of the spreading efficiency of biological and novel artificial microswimmers under flow conditions.

## Materials and methods

### Cell culture and staining

**Figure 1:**
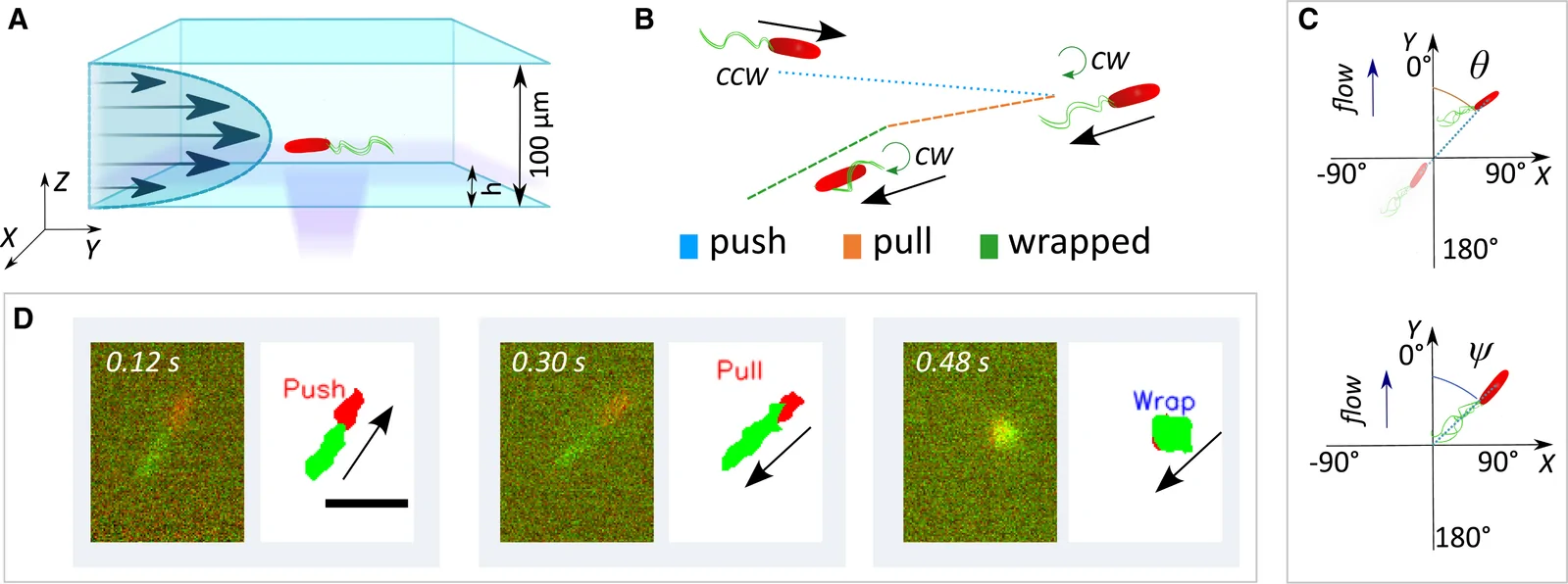
Experimental setup. (A) Bacterial suspension in Poiseuille flow in a microfluidic channel. Recordings are taken at a distance *h* above the surface. (B) Swimming modes of *P. putida*. (C) Angles in the x-y plane: *θ* is defined as the orientation of the velocity vector, *ψ* is the orientation of the cell body, i.e., the vector pointing from the center of mass of the flagellar bundle to the center of the cell body. (D) Examples of raw recording data and results of semi-automatic tracking (see the corresponding Videos S1 and S2 in the supplemental information). The arrows show the direction of the velocity vector in the co-moving frame; scale bar 5 μm.

We used the bacterial strain *Pseudomonas putida* KT 2440 *FliC*_S267C_ (later in the text referred to as “wild type”), where the protein FliC was genetically modified for fluorescent staining of the flagella bundle. These bacteria perform run-and-reverse motion in three swimming modes (push, pull, and wrapped; see Figure 1).^15^ To investigate the influence of the active propulsion, we tested a nonmotile strain for comparison. In this strain, the torque-generating stators MotAB and MotCD of the bacterial flagella were knocked out. The *P. putida* KT2440 *FliC*_S267C_ ΔmotAB ΔmotCD double knockout mutant was generated by sequential double homologous recombination as described elsewhere.^19^

A day before the experiment, a single colony was harvested from an LB-agar plate and transformed in 25 mL of tryptone broth media (10 g/L tryptone [AppliChem], 5 g/L NaCl). The solution was placed in an incubator at 30°C and shaken at 300 rpm for 14 h (up to a cell density of OD_600_ = 0.4). Later, washing with motility buffer (11.2 g/L K_2_HPO_4_, 4.8 g/L KH_2_PO_4_, 3.93 g/L NaCl, 0.029 g/L EDTA, and 5 g/L glucose), the fluorescent staining and further filtration of aggregates were done as described in Pfeifer et al.^40^ In short, a washed concentrated bacterial suspension was incubated with Alexa Fluor 488 C5-maleimide (Invitrogen, Thermo Fisher Scientific) over 30 min at room temperature for staining of the flagella. After that, the suspension was washed and re-incubated with the red membrane dye FM4-64 (Invitrogen, Thermo Fisher Scientific) to visualize the cell body. During the last step of staining, the bacterial suspension was gently filtered to remove aggregates of cells that appeared during intermediate centrifugation and washing steps. Finally, the cell suspension was resuspended to OD_600_ ∼0.2 (low-density suspension of cells, to avoid interactions of cells) in motility buffer, which forced the bacteria to stop growing but kept them motile.

### Microfluidic setup and image acquisition

The suspension of fluorescently stained cells was injected with a syringe into a rectangular polymer channel of type *μ*-Slide 0.1 VI, ibiTreat (Ibidi), with height 100 μm (the setup is shown in Figure 1A). For experiments under flow conditions, the bacterial suspension was pushed through the channel with a constant flow rate by a syringe pump (Harvard Apparatus, 11 Elite). To achieve a stationary state in the system, the pump was running 20 min before the first measurements. For the bulk experiments, the flow rates were 250 nL/min or 500 nL/min, resulting in local shear rates of *γ* = 1.5 s^−1^ or *γ* = 3.0 s^−1^, respectively, at a distance of *h* = 20 μm above the surface. For h = 5 μm above the surface, the flow rate was decreased to 125 nL/min to maintain a shear rate of *γ* = 3.0 s^−1^. The experiments with nonmotile mutant cells were carried out at *h* = 20 μm and under a local shear rate of *γ* = 3.0 s^−1^. The measurements were run 40 times (over 4 days) for the height above bottom surface *h* = 20 μm and shear rate *γ* = 3.0 s^−1^, 32 times (over 3 days) for *h* = 20 μm and *γ* = 1.5 s^−1^, 23 times (over 3 days) for *h* = 20 μm in the absence of flow, 10 times (on one day) for *h* = 5 μm and *γ* = 3.0 s^−1^, and 5 times (on one day) for the case of passive cells. On new measurement days, a fresh cell culture was used.

For fluorescence imaging, an inverted microscope (Olympus, IX71) was equipped with a monochromatic blue LED source with *λ* = 470 nm (Prizmatix, UHP-T-LED-470). For splitting the red signal of the cell body and the green signal of the flagella, the splitting optics (Hamamatsu, W-View Gemini) was used. The recordings were taken with a CMOS high-speed camera (Hamamatsu, ORCA-Fusion BT Digital CMOS camera, C15440-20UP) in a fixed focal plane at distances of *h* = 5 μm, or 20 μm above the bottom of the microfluidic channel (see Figure 1A). Images were acquired with a time resolution of 100 frames per second, using a 60x UPLFLN-PH objective (Olympus) with a small depth of field (DP = 0.7 μm) to obtain information from a thin layer of the sample.

### Image processing and cell tracking

To track and distinguish cells in different swimming modes, bacteria were stained with fluorescent dyes (red for the body and green for the flagella; see paragraph on “cell culture and staining” above and Figure 1B). Preliminary processing of the fluorescence recordings (channel splitting and frame cropping) was done with the freely available ImageJ software as described earlier.^15^ For further tracking and analysis, a custom-made Python code was used^41^ (see also supplemental information).

**Figure 2:**
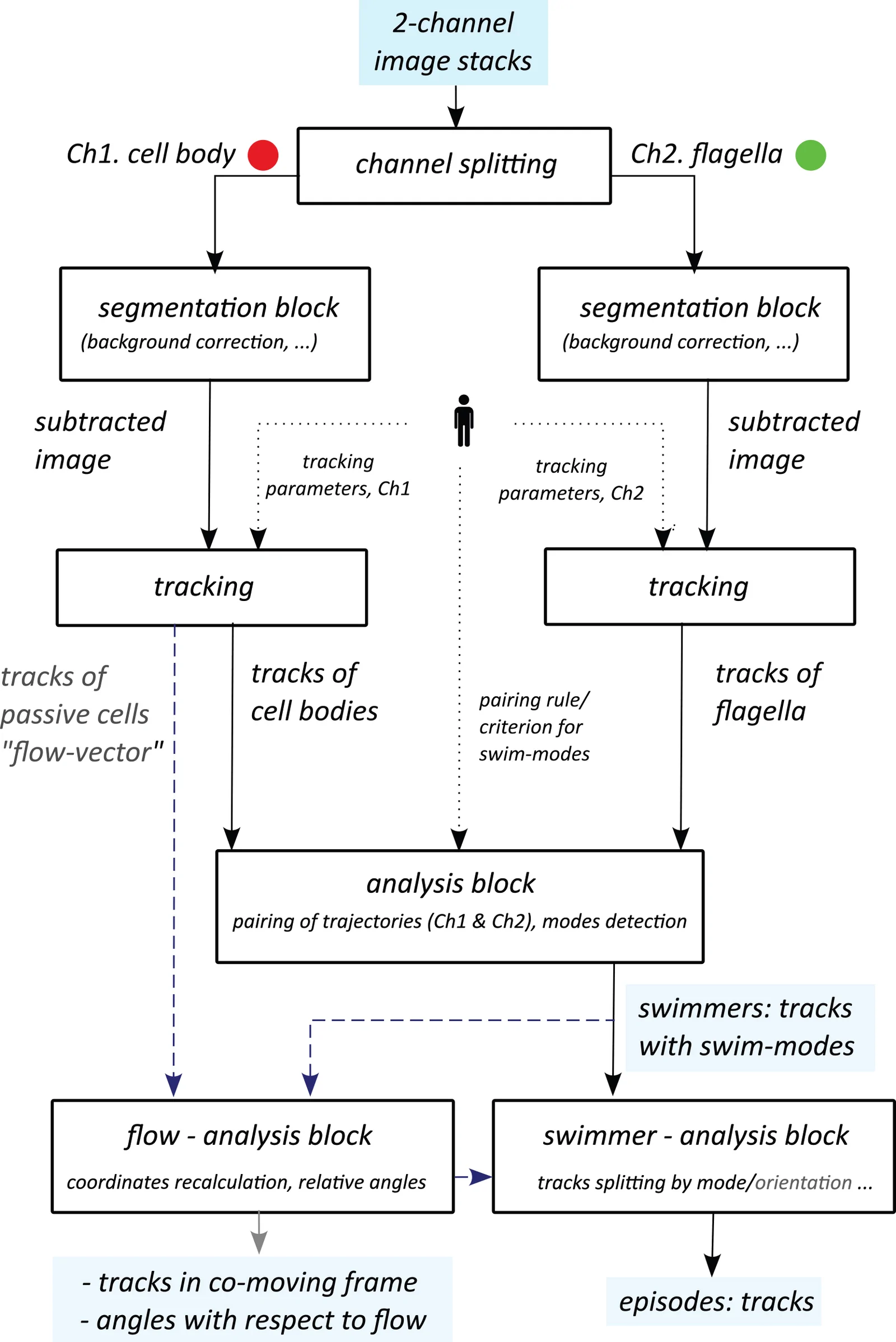
Diagram of the imaging analysis workflow. In the analysis block, the tracks of swimmers are considered as detected from the positions of the swimmers’ cell bodies unless otherwise stated. With the blue dashed arrows, the steps required for the flow subtraction are shown. We use the human icon and dotted arrows to mark the parameters that a user must specify manually.

The two channels corresponding to the fluorescence recordings of the red and green signals were segmented and tracked separately; see the workflow scheme in Figure 2. For every position in a trajectory in the red channel (associated with the center of mass of the cell body), the position of the corresponding trajectory in the green channel (associated with the center of mass of the flagellar bundle) of the same bacterium was determined based on a search radius, which was taken to be comparable to the size of the bacterium. The two points were then connected by a vector.

To subtract the flow velocity, separate recordings with only nonmotile cells were used for each measurement condition. The speed of well-resolved inactive cells was averaged and subtracted from the velocities of the cell bodies of the active swimmers obtained from the analysis of trajectories in the red channel. To distinguish between the swimming modes, the orientation of the vector pointing from the center of mass of the flagella to the center of the cell body with respect to the direction of cell displacement was analyzed; see Figure 1C. If the vector connecting flagella and cell body was pointing in the same direction as the displacement vector (after subtracting the fluid flow speed), the cell was assigned to be a pusher (see Figure S1; Table S1 in supplemental information). On the other hand, a puller was identified when these vectors were oriented in opposite directions. Finally, if the distance between the centers of mass of the cell body and the flagellar bundle fell below a threshold of 1.1 μm, the swimmer was assigned to move in wrapped mode (see also Table S1 in supplemental information). Prior to the swimming mode detection, trajectories were split into individual run episodes, for which the swimming modes were then identified separately; see also Figure 1B.

Note that this analysis relies on two types of orientation angles (see Figure 1C). The angle *ψ* characterizes the orientation of the cell body and is defined as the angle between the vector, which connects the centers of mass of the flagellar bundle and the cell body, and the direction of the fluid flow velocity, determined from the drift direction of nonmotile cells. The angle *ψ* can be determined for both pushers and pullers, but not for wrapped swimmers, due to the overlap of a cell body and a flagellar bundle. Also, for the passive nonmotile mutant cells, *ψ* can be determined. On the other hand, the angle *θ* represents the orientation of the swimming velocity vector with respect to the direction of fluid flow in the co-moving frame. The velocity vector is determined by connecting the cell body positions in adjacent time frames; see also Figure 1C and Figure S3 in supplemental information. The cell’s center of mass positions were detected for the time step *ΔT* = 0.05 s to determine the bacteria displacement vector and calculation of angle *θ*. To quantify differences between the angular histograms that were obtained under different flow conditions, a detailed statistical analysis was performed; see supplemental information.

### Mathematical framework

To simulate the hydrodynamics of a swimming bacterial cell in a viscous fluid, we employ the regularized Stokeslet formulation coupled with Kirchhoff rod theory, implemented within the immersed boundary framework, which is well suited for fluid-structure interaction problems.^38^^,^^42^ The cell body is modeled as a neutrally buoyant, rigid body, and the flagellum is treated as an elastic slender filament governed by Kirchhoff rod theory (see Figure S2; Table S2 in supplemental information). The flagellum is attached to the pole of the cell body to complete the full cell model, and this cell is immersed in an incompressible Newtonian fluid. The bacterial flagellar motor generates torque that drives the rotation of the flagellum. Simultaneously, an equal and opposite torque is applied to the cell body, resulting in its counterrotation. The swimming motion is calculated by enforcing force-free and torque-free conditions on the surrounding fluid. This framework enables the accurate capture of both direct mechanical interactions and fluid-structure coupling between the cell body and its motor-driven, flexible flagella. See supplemental information for a detailed mathematical description.

## Results

### Switches between swimming modes counteract alignment of bacteria in shear flow

**Figure 3:**
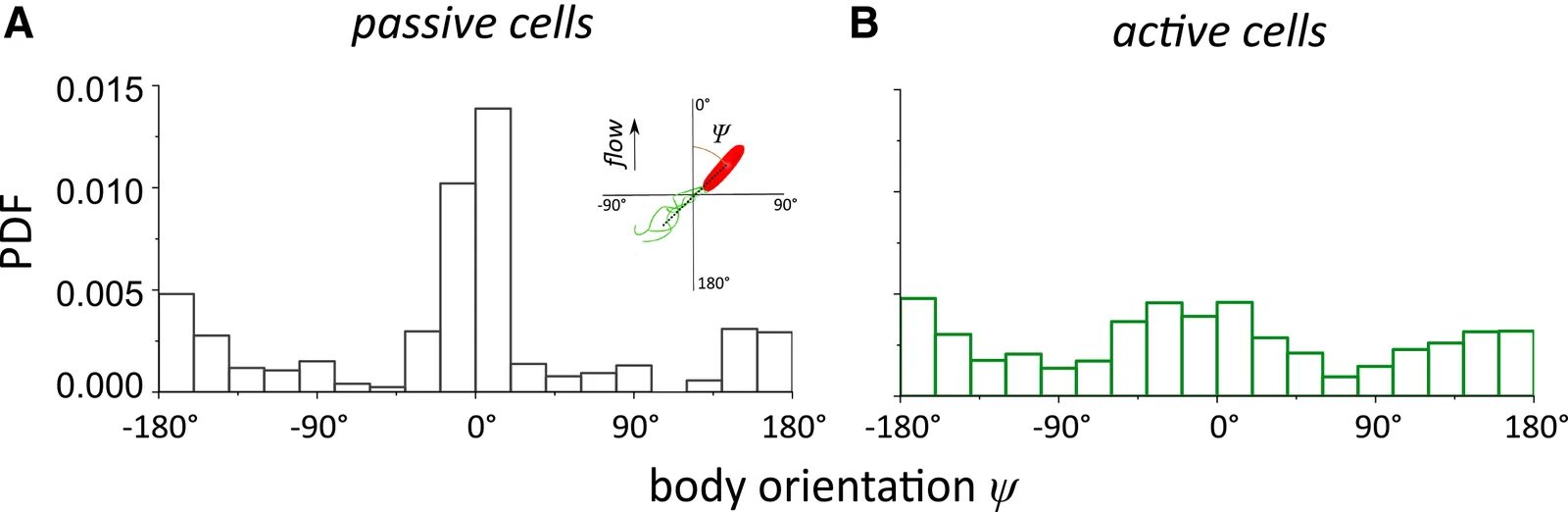
Flow alignment of cell bodies of motile and nonmotile bacteria. Distribution of body orientations (*ψ* angles) of (A) nonmotile mutant cells and (B) motile wild-type cells with respect to flow. Only unwrapped configurations (push and pull for swimming cells) were considered; shear rate *γ* = 3.0 s^−1^, measurement h = 20 μm above the bottom surface. Numbers of passive cells *n*_1_ = 19 (A) and active cells *n*_2_ = 116 (B). The number of data points are 3,319 frames (A) and 2,397 frames (B). For statistical analysis and comparison of the histograms, see supplemental information.

It is well known that the orientation of elongated objects in a moving fluid is affected by shear flow.^2^^,^^24^^,^^26^^,^^43^^,^^44^ Here, we analyze the impact of shear flow on the alignment of motile *P. putida* cells. We first elucidated the overall impact of motility on the alignment of rod-shaped, lophotrichously flagellated bacterial cells in a shear flow. We compared the cell body orientations (*ψ* angles) of motile *P. putida* wild-type cells with nonmotile *P. putida* mutant cells. A Δ*motAB* Δ*motCD* double mutant, deficient in both stators of the flagellar motor, was used as the nonmotile reference case, as these cells still carry flagellar filaments, but are unable to rotate their flagellar motors.^19^ For both wild-type and nonmotile cells, alignment with the flow direction was observed, indicated by peaks at 0° and 180° in the orientation angle *ψ* histograms displayed in Figure 3. However, as can be seen from the heights of the peaks in the histogram, the alignment effect was weaker for the motile wild type compared with the nonmotile mutant cells, where the alignment peaks were more pronounced.

### Shear-induced alignment of swimming trajectories is reduced close to surfaces

**Figure 4:**
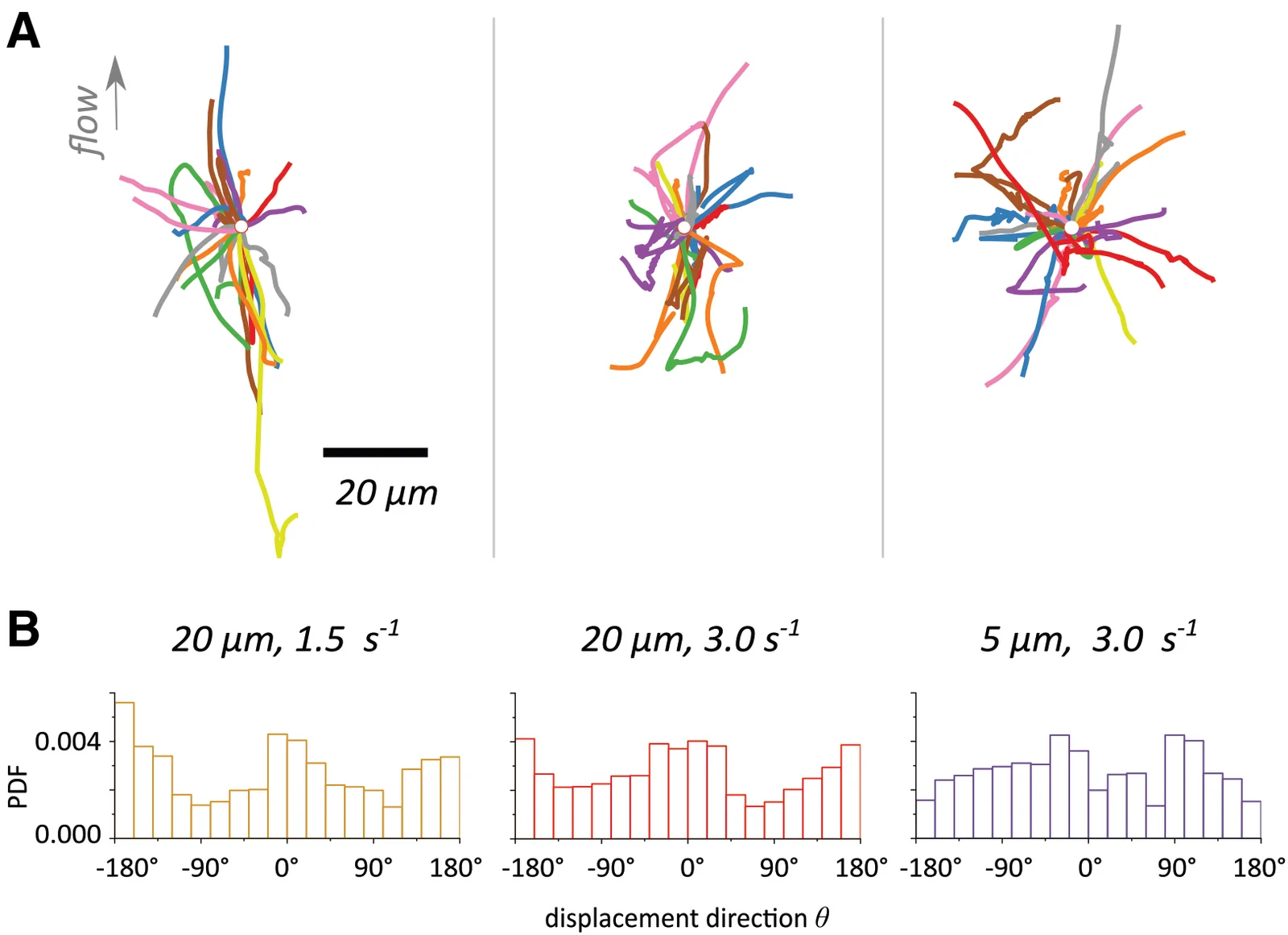
Alignment of bacterial locomotion for different shear rates *γ* = 1.5 s^−1^ and 3.0 s^−1^, and different distances *h* = 20 μm and 5 μm from the surface. (A) Typical swimmers’ trajectories in the co-moving frame, where the 25 longest tracks are shown. Flow speed is subtracted, flow direction is parallel with the Y axis, and the beginning of the tracks is marked by a white circle. (B) Distribution of *θ* angles (swimming direction) with respect to the flow in bulk (*h* = 20 μm) and near the surface (*h* = 5 μm). The time lag is Δ*T* = 0.05 s. Numbers of cells: *n*_1_ = 188 (left), *n*_2_ = 462 (middle), and *n*_3_ = 144 (right). The number of data points are 1,385 (left), 2,209 (middle), and 1,081 (right). For statistical analysis and comparison of the histograms, see supplemental information.

Even though switching between swimming modes compromises alignment in a shear flow, a clear preference to orient with the flow direction was observed. This was also reflected at the level of cell trajectories. In Figure 4A, the swimming trajectories of *P. putida* wild-type cells are shown for two different shear rates in the bulk fluid (at a distance of 20 μm away from the bottom of the microchannel) and in a shear flow close to a surface (at a distance of 5 μm from the bottom of the microchannel). The trajectories are displayed in a co-moving reference frame (i.e., the speed of fluid flow in the focal plane was subtracted), and their starting positions were centered at the origin.

For the two bulk fluid cases (left and middle), the pattern of trajectories is extended in flow direction, indicating an alignment of the direction of motion with the fluid flow. This is also reflected in the histogram of displacement angles *θ* shown in Figure 4B. For both shear rates, similar peaks at 0° and 180° indicate a preferred swimming direction with or against the fluid flow. In contrast, alignment vanished in the vicinity of the surface; see Figure 4, right-hand side. Here, the orientations of trajectories are more isotropic, and no clear peaks can be distinguished in the histogram of the displacement angle. Note that correlations in the data, resulting from the high degree of persistence during run phases, may lead to additional fluctuations in the histograms. For an in-depth statistical analysis that takes these effects into account and quantifies the differences between the histograms, see supplemental information.

### Pushers and pullers align more strongly than swimmers in wrapped mode

**Figure 5:**
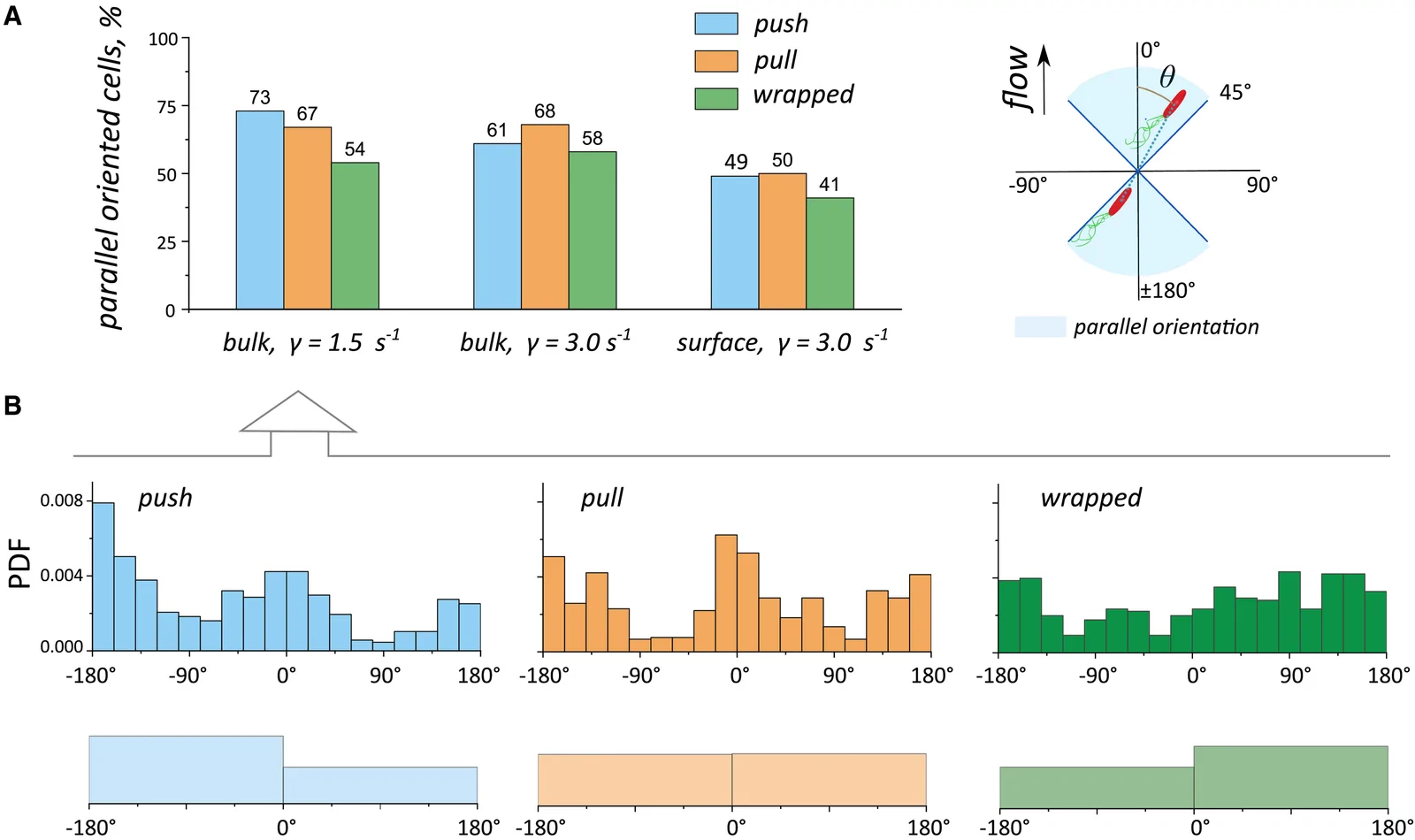
Flow alignment of bacteria in dependence on the swimming mode. (A) Percentage of displacements parallel to the direction of fluid flow, i.e., within a deviation from streamlines of less than ±45°; see insert on the right; “bulk” refers to *h* = 20 μm, and “surface” refers to *h* = 5 μm above bottom surface of the channel. (B) Distributions of *θ* angles is separated according to swimming modes (*h* = 20 μm, *γ* = 1.5 s^−1^). The time lag is Δ*T* = 0.05 s. Numbers of pushers *n*_1_ = 75 (left), pullers *n*_2_ = 70 (middle), and wrapped mode swimmers *n*_3_ = 43 (right). The number of data points are 437 (left), 521 (middle), and 427 (right). For statistical analysis and comparison of the histograms, see supplemental information.

In a fluid shear flow away from solid boundaries, we observed an overall alignment of swimming trajectories with the flow direction that breaks down close to a solid boundary. In order to find out to what extent alignment depends on the swimming mode, we performed dual-color fluorescence imaging experiments allowing us to identify for each run, whether the respective cells move in push, pull, or wrapped mode (relying on our semi-automated tracking and assignment software; see Figures 1D and 2 and materials and methods). Based on the assignments of runs to the different swimming modes, we quantified the orientations of cell displacements in the co-moving frame relative to the flow direction for each swimming mode separately. We classified a displacement as being aligned with the flow if its orientation deviated from the flow by less than 45° (with or against the flow direction; see schematic in Figure 5A). The bar plots in Figure 5A display the percentages of displacements aligned in flow direction for each swimming mode separately. The three different flow configurations correspond to the cases shown in Figure 4.

The bar plots confirm that in a shear flow far from solid surfaces, swimming directions preferentially align with the fluid flow, whereas alignment vanishes at the surface. With respect to the different swimming modes, these results moreover indicate that push and pull modes have an increased tendency to align as compared with cells swimming in wrapped mode. Comparing different shear rates far from the wall (left and middle bar plot), we observed that differences between the swimming modes became less pronounced at higher shear rate. As frequent turn events in the trajectories of active swimmers limit the degree of alignment that can be achieved, we speculate that with increasing shear rate, the different swimming modes eventually saturate at similar levels of alignment.

We also considered the temporal alignment dynamics of individual swimmers (see Figure S4 in supplemental information). For long episodes in push or pull mode, the *θ* angle mostly fluctuates around 180° (against flow direction). Cells in wrapped mode, in contrast, reorient faster and undergo more pronounced changes in their swimming direction. Note, however, that only small numbers of sufficiently long trajectories were available in our data, so that we cannot provide a rigorous statistical analysis of the alignment dynamics.

In Figure 5B, we consider the full histograms of displacement angles *θ* of all three swimming modes for a shear flow of *γ* = 1.5 s^−1^ and *h* = 5 μm away from a solid surface. In agreement with the bar plots in Figure 5A, the histograms of push and pull modes exhibit pronounced peaks at 0° and 180°, indicating alignment in flow direction, while no clear pattern is observed in the histogram of the wrapped mode. Interestingly, the histogram for *P. putida* swimmers in push mode additionally shows a preferred directional drift across streamlines in −*x* direction (see also Figure 1A), which means toward the right-hand side with respect to the negative flow (−*y*) direction. This can be seen as an indication of bacterial rheotaxis in bulk resulting from an interplay between the helical flagellar bundle and the velocity gradient of the fluid shear flow.^29^^,^^31^ Even though a similar chiral flagellar tuft is present in both pushers and pullers, contrary to our expectations, no drift was observed for runs in pulling mode. However, the compact wrapped configuration of the swimmer, which does not exhibit pronounced alignment with the streamlines, shows a directional bias to the opposite side, which, together with the behavior of pushers and pullers, will be considered in the following numerical simulations.

### Numerical simulations confirm different rheotactic responsiveness of pushers and pullers

To further substantiate whether *P. putida* cells swimming in push and pull modes show different sensitivity in their rheotactic response to shear flow, we performed numerical simulations. Our computational cell model is based on the real geometry of *P. putida*, with the exception that the cell has a single superflagellum at one pole. This simplification assumes that all flagella, when driven by a constant torque, are synchronized to form a flagellar bundle. For each swimming mode, the flagellar motor applies a specific constant torque to produce that mode. No tumbles or transitions between swimming modes occur within a given simulation. To mimic the experimental setting, each cell is positioned in a shear flow, such that the fluid velocity is zero in the focal plane where the cell is located. By imposing zero velocity in the focal plane, the flow field in our simulations corresponds to the co-moving frame of reference of the cell; i.e., the flow speed in the focal plane, as seen from the laboratory frame of reference, was subtracted. A uniform shear rate of *γ* = 3.0 s^−1^ was applied in all simulations, unless stated otherwise (see materials and methods and supplemental information for further details).

**Figure 6:**
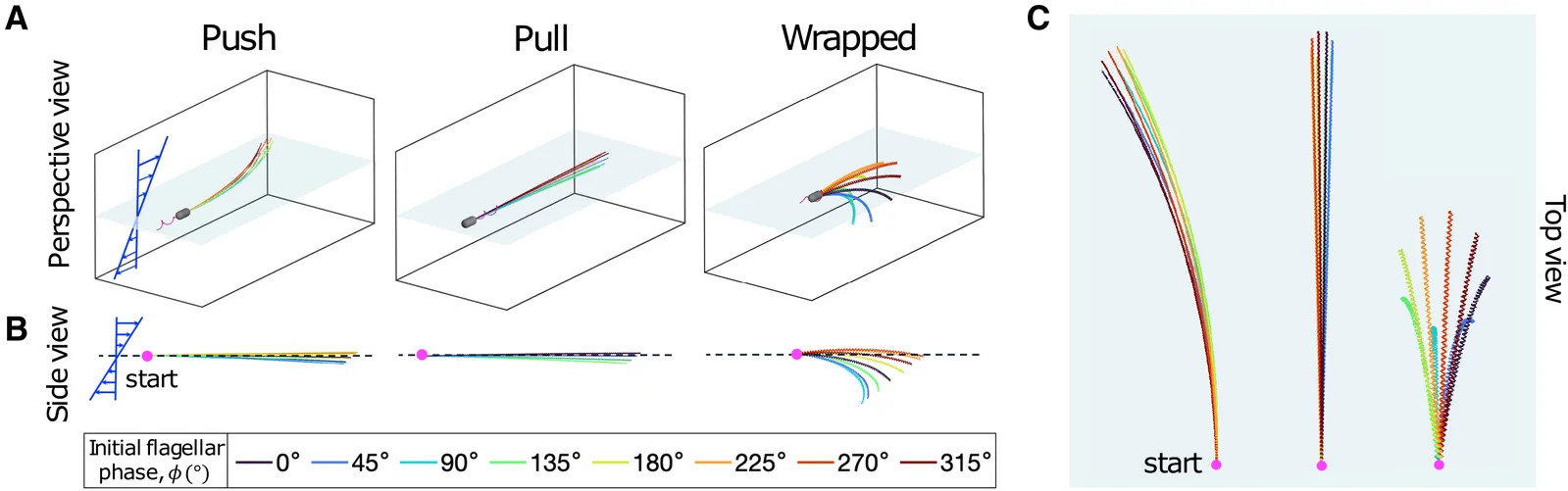
Numerical simulations: trajectories of bacteria in a shear flow. Trajectories of swimmers in push, pull, and wrapped modes under shear flow conditions. (A) Perspective, (B) side, and (C) top views are shown for each swimming mode. In each view, eight trajectories, corresponding to eight different initial phases *ϕ*, are displayed for each swimming mode. In the light blue plane, the flow velocity is zero. Each simulation is run for 0.8 s with a shear rate of *γ* = 3.0 s^−1^. The torque values for each mode are as follows: *τ* = 2.0 nN⋅nm for push, *τ* = −2.0 nN⋅nm for pull, and *τ* = −4.0 nN⋅nm for the wrapped mode. Above the plane of focus, the fluid moves in line with the initial swimming direction (swimming with the flow), while below it moves against the initial swimming direction (swimming against the flow). See also the corresponding Video S3 and additional numerical data in Figure S9 in the supplemental information.

Figure 6 shows trajectories of swimmers in different swimming modes and under shear flow conditions that we obtained from our numerical simulations. The plane of focus, where the flow velocity is zero, is indicated in light blue. The initial swimming direction was oriented within the plane of focus, aligned to the fluid flow in the adjacent planes. Above the plane of focus, the fluid moves in line with the initial swimming direction (swimming with the flow), while below it moves against the initial swimming direction (swimming against the flow). For each swimming mode, eight initial flagellar phases (*ϕ*) were considered, and the resulting trajectories are shown in three views: perspective, side, and top.

Similar to the experimental results in Figure 5B, also in the simulations, a drift motion across the streamlines was observed for a pusher configuration, while the puller stably follows the streamlines over time intervals even longer than the average duration of run episodes observed in experiments (see Table S3 in supplemental information). Interestingly, according to our simulations, swimmers in wrapped mode do not exhibit a directional bias but spread more rapidly in various directions as their trajectories are more strongly influenced by the initial flagellar phase than those of other swimming modes. Based on a larger set of numerical simulations that, in addition, also varies the initial body orientation of the swimmers in the shear flow, we recover histograms that capture most features observed in our experiments (see supplemental information).

### Pullers are stabilized under shear flow conditions

For *P. putida* cells swimming in a fluid at rest, the different swimming modes and the transitions between them have been studied in detail.^15^ When flagellar motors turn CCW, cells propagate in pushing mode with the flagellar bundle propelling the cell body from behind. Upon a switch to CW rotation, cells reverse their direction of motion and continue as pullers with the flagellar bundle pointing in the direction of motion. In most cases, pull runs are short-lived and transition to the wrapped mode of locomotion, where cells propagate in a screw thread fashion with their helical bundle wrapped around the cell body.^15^^,^^20^ Numerical simulations suggest that an increase in the torque of the flagellar motors triggers the transition from pull to wrapped mode, where swimming speed and persistence of motion are decreased but motors continue to rotate in CW direction.^45^ Once motors switch back to CCW rotation, swimming in wrapped mode ends, and the cell changes back to pushing mode.

Here, we experimentally studied whether shear flows affect the transitions and thus the frequencies at which the individual swimming modes are observed. Similar to earlier results,^19^ a fraction of push runs (CCW rotation of the flagellar motors) between 40% and 50% was observed in fluid at rest, as well as in two different shear flows; see Table 1. Within the fraction of pull and wrapped runs (CW rotation of the flagellar motors), we confirmed a dominance of the wrapped mode in resting fluid; see Table 1 and Pfeifer et al.^19^ However, under shear flow conditions, the wrapped mode is reduced to less than 50% within the population of CW runs and the pull mode dominates; see Table 1. The swimming speeds, by contrast, remained similar for cells in resting fluid and under shear condition, when measured at the same distance above the bottom surface of the channel; see Table S4 in supplemental information. These results suggest that filament wrapping becomes less likely in a fluid shear flow, thus stabilizing swimmers in pulling mode.

**Table 1:** Statistics of occurrence of swimming modes

Shear rate *γ*, s^−1^	Numbers of cells	Frequency of mode, %			Ratio CW pull/wrap
		push	pull	wrapped	
0.0	116	45.7	21.5	32.8	0.66
1.5	188	39.9	37.2	22.9	1.62
3.0	462	43.7	30.1	26.2	1.15

Contributions of the three swimming modes to the total number of runs in fluid at rest and in two different shear flows. All data were recorded at a distance of *h* = 20 μm away from the surface.

### Numerical simulations: Critical torque for filament wrapping is increased in fluid shear flow

To confirm that filament wrapping is indeed affected under shear flow conditions, we performed numerical simulations of cells transitioning from the pull to the wrapped mode under shear flow, with the flagellar motor rotating CW, corresponding to negative values of the motor torque (*τ*). Each cell in pull mode was initially positioned in the focal plane, where the fluid velocity was zero; i.e., numerical simulations were performed in the co-moving frame of reference, as described above. Various combinations of motor torque (*τ*), shear rate (*γ*), and initial flagellar phase (*ϕ*) were considered.

**Figure 7:**
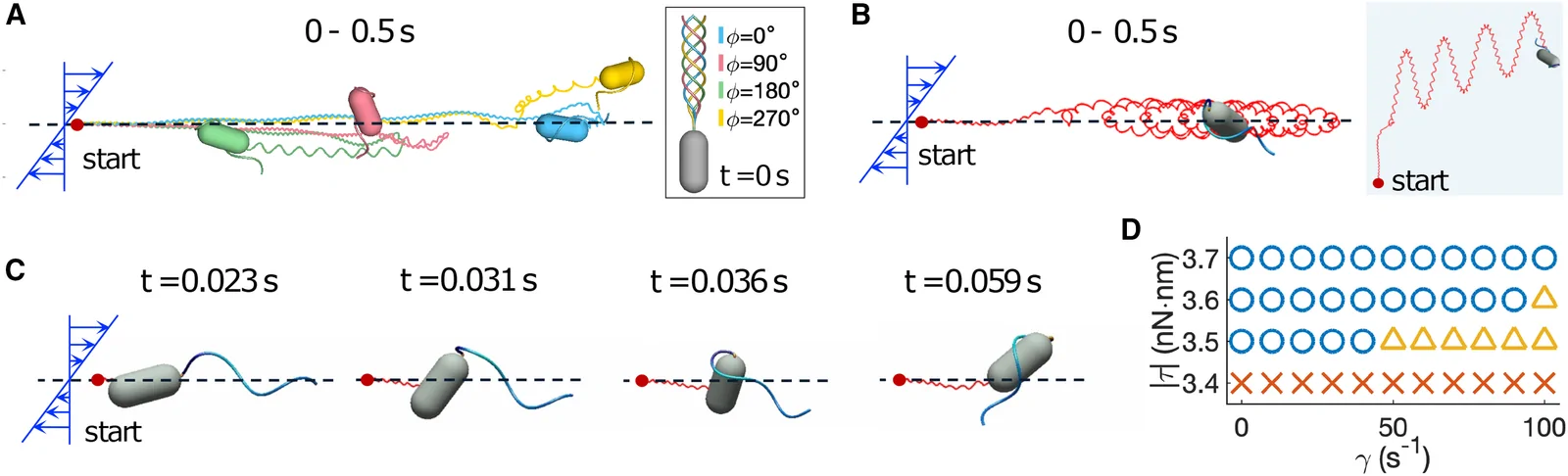
Numerical simulations: wrapped mode formation under shear flow. (A) Tracks of swimmers undergoing a pull-to-wrap transition for four different initial flagellar phases *ϕ* (see inset). The dashed line indicates the focal plane, where the flow speed is zero. The applied torque and the shear rate are *τ* = −3.5 nN⋅nm and *γ* = 20 s^−1^, respectively. (B) Typical periodic trajectory of a wrapped swimmer at high shear rates (*τ* = −3.7 nN⋅nm, *γ* = 50 s^−1^, and *ϕ* = 0°). The left and right panels correspond to side and top views, respectively. (C) Snapshots showing time evolution of a cell’s transition into the wrapped mode, *τ* = −4.0 nN⋅nm, *γ* = 50 s^−1^, and *ϕ* = 90°. (D) Motor torque thresholds (|*τ*|) for the formation of the wrapped mode at different shear rates (*γ*). Circles represent the wrapped mode, crosses represent the pull mode, and triangles represent cases where either mode may occur depending on the initial flagellar phase. See also the corresponding Videos S4, S5, and S6 in the supplemental information.

Figure 7A displays four trajectories corresponding to four distinct initial flagellar phases (*ϕ* = 0°, 90°, 180°, and 270°; see inset), while all other parameters were held constant. It can be seen that the flagellar phase critically influences the turning angle during a transition from pull to wrapped mode. As a result, the initial flagellar phase affects subsequent bacterial spreading in the wrapped mode. Depending on the interplay of turning angle and shear flow geometry, different complex trajectory patterns may emerge. Figure 7B illustrates, as an example, the long-time periodic trajectory of a wrapped swimmer observed at high shear rates. The left and right panels correspond to the side and top views, respectively, as in Figure 6. After the flagellum wrapped around the cell body, the wrapped-mode swimmer oscillated across the focal plane between the regions of opposing flow directions. The detailed temporal evolution of a cell’s transition into the wrapped mode can be seen in Figure 7C for *τ* = −4.0 nN⋅nm, *γ* = 50 s^−1^, and *ϕ* = 90°. At this torque magnitude, the flagellum buckles, causing the cell body to turn out of the focal plane, and hydrodynamic interactions lead to filament wrapping.

Finally, in Figure 7D, we show the resulting swimming mode as a function of motor torque and shear rate, starting from a pull mode swimmer with the motor rotating CW. As has been shown in previous simulations in resting fluid, a critical motor torque is required to initiate filament wrapping.^46^ This is also confirmed here, where the pull mode persists for small motor torques, independent of the shear rate. Our simulations under flow conditions show that the critical torque that is required to initiate filament wrapping increases with increasing shear rate. Note that, for each choice of torque and shear rate, eight initial flagellar phases uniformly distributed over 360° were considered. The resulting swimming mode is generally independent of the initial phase, except near the threshold values, where different initial phases may lead to either pull or wrapped modes.

## Discussion

The environmental conditions of bacterial habitats, such as complex geometries or fluid flows, may strongly affect bacterial locomotion and navigation. In this work, we examined the impact of fluid shear flow on the motility pattern of the gram-negative soil bacterium *P. putida*, a lophotrichously flagellated, rod-shaped swimmer that exhibits runs in push, pull, and wrapped mode and serves as a model for multi-mode bacterial swimming.

We showed that active swimming perturbed shear-flow-induced alignment of the bacterial body axis with the flow direction. This can be attributed to the turning maneuvers that occur between run phases and repeatedly randomize the direction of motion and, hence, the body orientation of the motile wild-type cells. Nevertheless, under shear flow conditions in the bulk fluid (20 μm above the bottom surface of the channel), swimming trajectories exhibit a clear tendency to align with the flow direction. In contrast, close to a solid surface (5 μm above the bottom surface of the channel), flow alignment of the trajectories was no longer detectable. We assume that this is due to the wall-induced reorientation of swimmers parallel to the solid surface, so that the rod-shaped cell bodies are less affected by the shear gradient perpendicular to the surface.^44^

By considering runs in push, pull, and wrapped modes separately, we could show that alignment is more pronounced for pushers and pullers, while swimmers in wrapped mode showed no clear tendency, neither far nor near the interface. This is presumably related to the compact shape of the wrapped mode as compared with the more elongated configurations of pushing and pulling swimmers (according to our fluorescence images, the aspect ratio of unwrapped swimmers is *α* ≃ 7.5, while for wrapped swimmers it is only *α* ≃ 2.5). At the same time, a rheotactic drift across streamlines was observed for swimmers in push mode in agreement with earlier literature.^29^^,^^30^^,^^32^^,^^47^ Unexpectedly, however, only for the wrapped mode swimmer, an analogous effect was observed, while the trajectories of pullers were not affected over the time window of our observation. Numerical simulations relying on realistic swimmer geometries confirmed the experimental observations for pushers and pullers but did not show any directional preference for the wrapped mode swimmers. The absence of a rheotactic drift of pullers in both our experimental and numerical data does not agree with our current understanding of rheotaxis as a consequence of chirality-induced cell reorientation in a shear flow and remains an open question that requires further mechanistic studies.

As swimmers are floating along with the fluid flow, transitions between swimming modes and the associated directional changes may enhance the spreading efficiency. For example, a run-and-reverse swimming strategy was shown to be advantageous for efficiently tracking small food sources in dynamic fluid environments.^48^ Here, we demonstrated that the transitions between swimming modes are affected by fluid shear flow conditions. Specifically, we observed that among the runs with CW-rotating motors, the ratio between straight runs in pull mode and less persistent runs in wrapped mode is shifted in favor of pullers, indicating that the pull mode becomes more stable in a shear flow. This is in line with earlier observations in *E. coli* showing that run times and flagellar transitions are affected by the presence of a fluid flow.^49^ Our numerical modeling results suggest that the pull mode is stabilized due to mechanical interactions between the flagella and the fluid shear flow, where different fluid velocities in the vicinity of the cell body make filament wrapping more difficult. Thus, an increased critical torque is required for wrapping in a shear flow, as demonstrated by our systematic numerical simulations. As a result, more pullers and fewer wrapped swimmers are observed in a shear flow as compared with liquid at rest.

Note that in the experiments, wrapped mode formation is already reduced at shear rates of *γ* = 1.5 s^−1^ and 3.0 s^−1^, while in numerical simulations an impact of the shear flow on wrapped mode formation is only observed for shear rates beyond *γ* = 50.0 s^−1^. Besides uncertainties in some of the mechanical parameters of our numerical model, we assume that the main reason for this discrepancy is the number of flagella that was included in our numerical study. While *P. putida* carries a tuft of 5–7 flagella attached to one pole of the cell body, we approximate this flagellar bundle by a single helical filament in our model simulations. This may serve as a good approximation for a swimmer that moves in push mode. A pulling helical bundle, however, is less stable and prone to jamming at the filament intersection points.^50^^,^^51^ This may facilitate transitions to the wrapped mode, so that lower shear forces are sufficient to hinder the pull-to-wrapped transition in the case of a filament bundle, while higher shear is required to suppress the wrapping of a single filament. Note also that this purely mechanical scenario does not involve any active regulation via receptor-mediated signaling.

Future studies of bacterial swimmers will focus on combining environments of complex geometry with hydrodynamic flows to mimic real-world habitats even more closely. They may also serve as a source of insight for the navigation of artificial microswimmers and may be combined with advanced techniques to induce time-dependent local flow fields by bio-compatible light-sensitive materials.^52^^,^^53^ Such experiments may also include mutant cell lines that allow for the adjustment of run lengths, stop events, and transitions from one swimming mode to another. Here, constructing so-called nontumbling mutants that propagate as push-only, pull-only, or wrapped-only swimmers will be particularly instructive to study the role of the different swimming modes within the complex environment.

## Conclusion

In this study, we investigated how fluid shear flow influences the swimming behavior of *Pseudomonas putida*, a soil bacterium known for its multi-mode swimming strategy. Our experiments revealed that shear-induced alignment and rheotactic drift responses vary depending on the swimming mode. We furthermore observed that flagellar wrapping becomes less effective under higher shear stress. Numerical simulations based on realistic swimmer geometries support the experimental findings. While still at an early stage, we hope to contribute with our findings to predicting the spreading of bacterial infections in complex surroundings as well as to the engineering of artificial microswimmers designed to navigate specific habitats.

## Data availability

The original code has been deposited at Zenodo under https://doi.org/10.5281/zenodo.17456571 and is publicly available as of the date of publication. Any additional information required to reanalyze the data reported in this paper is available from the lead contact upon request.

## Supplemental citation

Reference 54 is cited in the supplemental information.

## Acknowledgments

V.F. acknowledges the kind hospitality of the Erwin Schrödinger Institute (ESI) in Vienna during the workshop “Nonequilibrium processes in Physics and Biology,” where part of this project was productively discussed. The research has been partially funded by the International Max Planck Research School on Multiscale Bio-Systems (V.F.) and by the 10.13039/501100001659Deutsche Forschungsgemeinschaft, Germany, project ID 443369470 – BE 3978/13-1 (V.F., V.P., and C.B.) as well as project ID 318763901 – SFB1294 (A.D., V.P., R.G., and C.B.). S.L. was supported by the NSF (CBET-2415406) and the Charles Phelps Taft Research Center at the University of Cincinnati, USA. Y.K. was supported by the 10.13039/501100003725National Research Foundation of Korea, South Korea (RS-2023-00247232). W.L. was supported in part by the National Institute for Mathematical Sciences, South Korea (B25910000) and in part by the 10.13039/501100003725National Research Foundation of Korea,South Korea (MSIT) (RS-2025-16071334). During most of the period in which this work was carried out, J.P. was a member of the Institute for Advanced Study, supported by the AMIAS Fund, with access to IAS computing resources.

## Author contributions

V.F. designed and conducted experiments, analyzed experimental data, and drafted the manuscript. A.D. designed the image analysis toolbox, analyzed experimental data, and wrote the manuscript. S.L. and J.P. designed the numerical settings and wrote the manuscript. J.P., Y.K., and W.L. performed numerical simulations. J.P. processed and visualized numerical data. R.G. performed statistical analysis and Bayesian inference of bacterial alignment and wrote the corresponding section in the supplemental information. V.P. established the fluorescent staining procedure and generated the stator mutant. C.B. designed the research and wrote the manuscript. C.B. and S.L. supervised the whole project. All authors reviewed and edited the manuscript, discussed the results, and contributed to the final manuscript.

## Declaration of interests

The authors declare no competing interests.

## Supplemental information

Document S1. Figures S1–S9, and Tables S1–S4

Video S1. Examples of raw data and results of the image analysis toolbox processing as described in the main text

Video S2. Examples of raw data and results of the image analysis toolbox processing as described in the main text

Video S3. Numerical simulations: trajectories of cells swimming in a shear flow

Video S4. Numerical simulations: trajectories of cells transitioning from pull to wrapped under shear flow

Video S5. Numerical simulation: long-time periodic trajectory of a wrapped swimmer at high shear rate

Video S6. Numerical simulation: time evolution of a cell that transitions into the wrapped mode under shear flow

Document S2. Article plus supplemental information
