# Supplementary material for: Swimming patterns of a multi-mode bacterial swimmer in fluid shear flow
Source: Biophys J. 2026 May 30;125(14):3547–57. doi: 10.1016/j.bpj.2026.05.032 (PMC13507449; doi:10.1016/j.bpj.2026.05.032)
Supplement: Supplementary file 1 [file mmc1.pdf]

Biophysical Journal, Volume *125*

Supplemental information

Swimming patterns of a multi-mode bacterial swimmer in fluid shear flow

Valeriia Fast, Agniva Datta, Jeungeun Park, Robert Großmann, Veronika Pfeifer, Yongsam Kim, Wanho Lee, Sookkyung Lim, and Carsten Beta

SUPPLEMENTAL MATERIALS

1 Semi-automatic analysis of trajectories

In this section, we discuss how the trajectories are obtained from microscope images and the criteria we use to identify the different swim modes. To track and distinguish cells in different swimming modes, bacteria are stained with fluorescent dyes (red for the body and green for the flagella), see Main Text and [1]. Preliminary processing (signal splitting, frame cropping) of the fluorescent recordings is done with freely available *ImageJ* software as described in the Main Text [2]. For further tracking and analysis, the lab-made Python code [3] is used, which is provided along with this manuscript. The workflow is also shown in Figure 2 in the Main Text.

1.1 Cell tracking

The raw images from the microscope are a stack of *uint16* images consisting of two colored channels, namely the red and the green ones, which capture the staining of the cell body and the flagella bundle of the bacterial cells, respectively. As a first step, we split the image stacks into the red and green channels, thereby obtaining a pair of grey-scale image stacks. Then we perform segmentation and tracking separately on the two image stacks to extract the trajectories corresponding to the center of masses of the segmented cell body and flagella of each moving cell. For the last step, we use the method described in publication [4], which is based on Crocker's processing algorithm. Detailed description of the last step can be found in [5].

1.2 Distinction of swim-modes

We match the coordinates of the cell trajectories (obtained from the red channel) and the flagella trajectories (obtained from the green channel). Based on a minimum displacement δx and minimum time difference δt between the coordinates of each point in the cell and flagella trajectories, we identify the pairs of trajectories which correspond to the individual bacteria. Then, for each identified pair and each frame of the image stacks, we define a vector from the coordinate of the center of mass of a cell to that of the corresponding identified flagella bundle, which we call the pointing vector $\boldsymbol{P}_{i,j}$ and also the velocity vector $\boldsymbol{v}_{i,j}$ corresponding to the displacement of the cell from the previous frame (see Figure S1)., defined as follows:

$$\boldsymbol{P}_{i,j} = \left(x^f_{i,j} - x^c_{i,j}\right)\hat{i} + \left(y^f_{i,j} - y^c_{i,j}\right)\hat{j}, \tag{1a}$$

$$\boldsymbol{v}_{i,j} = \left(x^c_{i,j} - x^c_{i-1,j}\right)\hat{i} + \left(y^c_{i,j} - y^c_{i-1,j}\right)\hat{j}. \tag{1b}$$

Here, the index i corresponds to the ith frame of the image, index j corresponds to the jth pair of tracks (one bacterium), index c corresponds to the coordinates of the center of mass of cell (obtained from red channel) and the index f corresponds to the coordinates of the center of mass of flagella bundle (obtained from green channel). We determine the swim-mode corresponding to the jth bacterium in the ith frame by the inner product of $\boldsymbol{P}_{i,j}$ and $\boldsymbol{v}_{i,j}$ as shown in Table S1.

It is important to note here that the distinction is based on the parameters δx, δt and ϵ, which show little variability from image to image, especially for separate datasets due to the heterogeneity of the bacterial population and quality of recorded images. Further, to calculate the flow drift velocity, we analyze some non-swimming cells (pre-selected manually) in the cell suspension. Then, we perform segmentation and tracking on the red channel of the recorded images. Thereby, we obtain the drift velocity $\boldsymbol{v}^{\text{drift}}_{i,j}$ corresponding to the

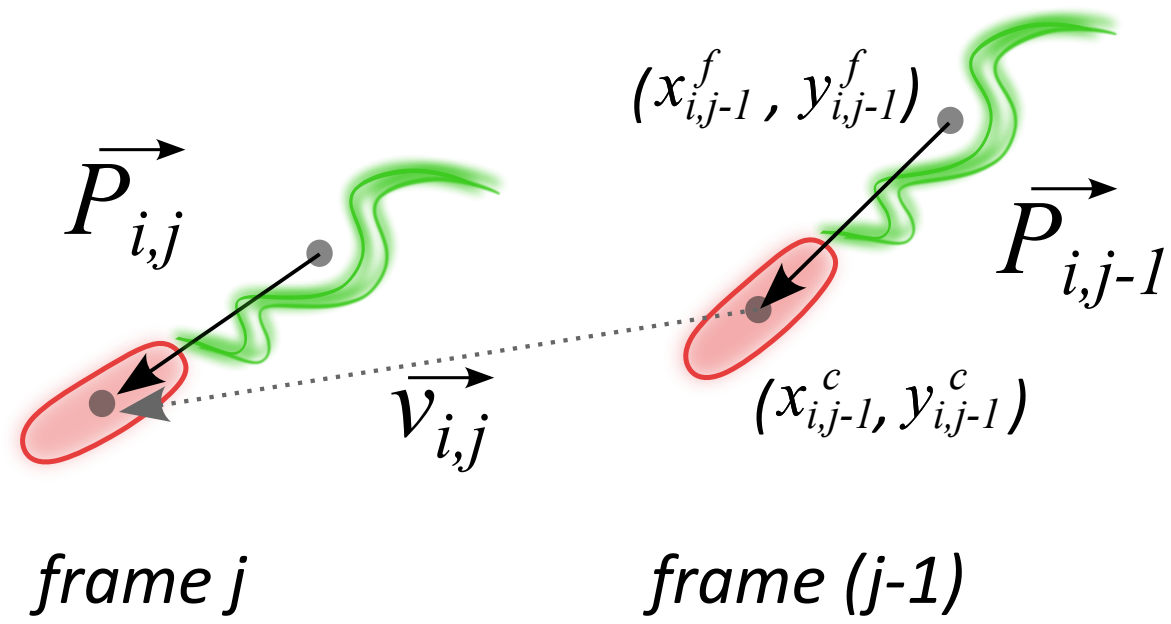

Figure S1: Schematic illustration of vectors of a single swimmer for the swim-mode detection.

swim-mode	criteria
push	$\boldsymbol{P}_{i,j}.\boldsymbol{v}_{i,j} > 0$
pull	$\boldsymbol{P}_{i,j}.\boldsymbol{v}_{i,j} < 0$
wrapped	$\lvert\boldsymbol{P}_{i,j}\rvert < \epsilon$

Table S1: Criteria to identify swim-modes from a pair of trajectories in a particular frame. The threshold for cell body-flagella is distance $\epsilon = 1.1\,\mu\text{m}$ for *P. putida* cells.

flow by taking the average of the velocities calculated from the derived trajectories. Then, we modify the detection criteria by taking the inner product of $\boldsymbol{P}_{i,j}$ and the relative velocity $\boldsymbol{v}^{\text{rel}}_{i,j}$, obtained by subtracting the flow velocity from the observed velocity.

$$\boldsymbol{v}^{\text{rel}}_{i,j} = \boldsymbol{v}_{i,j} - \boldsymbol{v}^{\text{drift}}_{i,j} \tag{2}$$

After, the same criteria are used to distinguish the swim-modes by replacing $\boldsymbol{v}_{i,j}$ by $\boldsymbol{v}^{\text{rel}}_{i,j}$ in Table S1.

Also, $\boldsymbol{v}^{\text{drift}}_{i,j}$ vector is used to define the orientations of the cells with respect to the flow streamlines. We define the angle between the pointing vector $\boldsymbol{P}_{i,j}$ and fluid flow direction, calculated above, as ψ angle (body's orientation), and the angle between the cell's velocity vector $\boldsymbol{v}_{i,j}$ and fluid flow direction as θ angle (the direction of the displacement), see also Figure 1D in the Main Text. The ψ angles (body's orientation) are defined only for unwrapped cells' configurations (push, pull and mutant non-motile cells).

2 Mathematical framework

P. putida consists of a rod-shaped cell body with a tuft of multiple flagella anchored at one pole, and it propels itself through fluid via flagellar rotation. Our computational model is constructed based on the experimentally observed geometry of *P. putida* and incorporates the relevant biological data. For simplicity, the cell model includes a single polar flagellum representing a bundled configuration of multiple flagella. The initial setting and parameter values used in the simulations are provided in Figure S2 and Table S2, respectively.

Flagellar dynamics is modeled using Kirchhoff rod theory, while the cell body is treated as a rigid body. The forces and torques generated by flagellar rotation are transmitted to the surrounding fluid which is governed by the incompressible Stokes equations. To solve these coupled equations, we employ the immersed boundary framework that is useful to solve fluid-structure interaction (FSI) problems. This approach captures the hydrodynamic interactions between the cell and the fluid, enabling bidirectional coupling between the cell motion and the resulting fluid flow. Importantly, the flagellar motor generates a constant torque, introduced as a control parameter, that drives flagellar rotation, while an equal and opposite torque is applied to the cell body, resulting in its counterrotation. The swimming motion is computed

by enforcing the condition that the net force and torque exerted on the surrounding fluid are both zero. Additionally, in the presence of shear flow, another parameter is introduced to control the imposed shear rate. We begin by describing the motion of a model cell composed of a rod-shaped cell body and a single left-handed superflagellum, formulated in Lagrangian coordinates. Subsequently, we present the governing fluid equations using the regularized Stokeslet formulation expressed in Eulerian coordinates. A detailed description of the method can be found in [6, 7] and therein; key features of the approach are summarized below.

The cell body is modeled as a spherocylinder and discretized into n_b points on its surface. It is represented in two forms, $\mathbf{X}_i^\mathrm{b}(t)$ and $\mathbf{Y}_i^\mathrm{b}(t)$, for $i = 1, 2, ..., n_\mathrm{b}$. The former corresponds to the physical body that interacts with the surrounding fluid, while the latter represents a rigid body that does not directly interact with the fluid. Each pair of corresponding points $\mathbf{X}_i^\mathrm{b}(t)$ and $\mathbf{Y}_i^\mathrm{b}(t)$ is connected via a stiff spring which generates a restoring force of the form

$$\mathbf{F}_i^b(t) = K(\mathbf{X}_i^\mathrm{b}(t) - \mathbf{Y}_i^\mathrm{b}(t)), \tag{3}$$

where K is a large stiffness constant that ensures the two representations remain close to each other. The force $\mathbf{F}_i^b(t)$ is applied to the rigid body at $\mathbf{Y}_i^\mathrm{b}(t)$, while an equal and opposite force $-\mathbf{F}_i^b(t)$ is exerted on the fluid at $\mathbf{X}_i^\mathrm{b}(t)$. The reference configuration of the rigid cell body is defined by a set of time-independent points $\mathbf{Z}_i$ that satisfy $\sum_{i=1}^{n_\mathrm{b}} \mathbf{Z}_i = \mathbf{0}$. The position of the rigid body at time t is then given by

$$\mathbf{Y}_i^\mathrm{b}(t) = \mathbf{C}(t) + \mathcal{R}(t)\mathbf{Z}_i, \tag{4}$$

where $\mathbf{C}(t)$ is the centroid of the rigid cell body and $\mathcal{R}(t)$ is a rotation matrix.

Let $\mathbf{f}^\mathrm{b}(t)$ and $\mathbf{n}^\mathrm{b}(t)$ denote the external force and torque, respectively, acting on the cell body, excluding contributions from the spring connections. Then, the balance of linear and angular momentum for the rigid body is given by

$$0 = \mathbf{f}^\mathrm{b}(t) + \sum_{i=1}^{n_\mathrm{b}} \mathbf{F}_i^\mathrm{b}(t), \qquad 0 = \mathbf{n}^\mathrm{b}(t) + \sum_{i=1}^{n_\mathrm{b}} (\mathcal{R}(t)\mathbf{Z}_i) \times \mathbf{F}_i^\mathrm{b}(t). \tag{5}$$

Given $\mathbf{f}^\mathrm{b}(t)$, $\mathbf{n}^\mathrm{b}(t)$ and $\mathbf{X}_i^\mathrm{b}(t)$ at each time t, we can compute $\mathbf{C}(t)$, $\mathcal{R}(t)$, and thus $\mathbf{Y}_i^\mathrm{b}(t)$ by solving the above system approximately [7]. The accuracy of the approximation improves with increasing spring stiffness K.

The motion of a flagellum is described by Kirchhoff rod theory. A flagellum is represented as a tapered helical curve $\mathbf{X}(s,t)$ together with orthonormal triads $\{\mathbf{D}^1(s,t), \mathbf{D}^2(s,t), \mathbf{D}^3(s,t)\}$ along the curve. This curve represents the centerline of the flagellum and the triads show the degree of bending and twisting. Let $\mathbf{F}(s,t)$ and $\mathbf{N}(s,t)$ be the force and the moment, respectively, which are transmitted across a section of the rod at the Lagrangian coordinate s at time t. Let $\mathbf{f}(s,t)$ and $\mathbf{n}(s,t)$ be the applied force and the torque densities, respectively. Then the linear and angular momentum balance equations are described as follows:

$$0 = \mathbf{f} + \frac{\partial \mathbf{F}}{\partial s}, \qquad 0 = \mathbf{n} + \frac{\partial \mathbf{N}}{\partial s} + \frac{\partial \mathbf{X}}{\partial s} \times \mathbf{F}. \tag{6}$$

Here, the internal force $\mathbf{F}$ and torque $\mathbf{N}$ can be written as follows:

$$\mathbf{F} = \sum_{i=1}^{3} b_i \Big(\mathbf{D}^i \cdot \frac{\partial \mathbf{X}}{\partial s} - \delta_{3i}\Big)\mathbf{D}^i, \qquad \mathbf{N} = \sum_{i=1}^{3} a_i \Big(\frac{\partial \mathbf{D}^j}{\partial s} \cdot \mathbf{D}^k - \kappa_i\Big)\mathbf{D}^i, \tag{7}$$

where δ_{3i} is the Kronecker delta, (i, j, k) is a cyclic permutation of $(1, 2, 3)$, and κ_i describes the intrinsic curvature (κ_1 and κ_2) and twist (κ_3). The constitutive relations can be derived from a variational derivative of the elastic energy functional for the unconstrained version of the Kirchhoff rod,

$$E = \frac{1}{2}\Big[\sum_{i=1}^{3} a_i \Big(\frac{\partial \mathbf{D}^j}{\partial s} \cdot \mathbf{D}^k - \kappa_i\Big)^2 + \sum_{i=1}^{3} b_i \Big(\mathbf{D}^i \cdot \frac{\partial \mathbf{X}}{\partial s} - \delta_{3i}\Big)^2\Big] ds, \tag{8}$$

where the parameters a_1 and a_2 are bending moduli, a_3 is the twist modulus, b_1 and b_2 are shearing moduli, and b_3 is the stretching modulus. To model the compliant hook, the bending modulus of the hook a^hook is reduced by a factor of 25 compared to that of the flagellum, reflecting its increased flexibility.

The tapered end of the flagellum is embedded into the cell body at the pole, with the flagellar axis aligned with the normal direction $\mathbf{E}(t)$ to the cell surface. The flagellar motor generates a constant torque τ in this normal direction at the motor point $\mathbf{X}(s_{\mathrm{mot}}, t)$, given by $\mathbf{N}(s_{\mathrm{mot}}, t) = -\tau\mathbf{E}(t)$. Positive values of τ correspond to counterclockwise rotation, while negative values correspond to clockwise rotation. This motor torque drives the rotation of the flagellum, enabling the cell to swim through the surrounding fluid.

Finally, the cell motion described above is coupled with the surrounding fluid which is governed by the incompressible Stokes equations:

$$0 = -\nabla p + \mu\Delta\mathbf{u} + \mathbf{g}, \qquad 0 = \nabla\cdot\mathbf{u}, \tag{9}$$

where the fluid velocity $\mathbf{u}$ and pressure p are unknown functions of the Cartesian coordinates $\mathbf{x}$ and time t, and μ is the dynamic viscosity of the fluid. The regularized force density $\mathbf{g}$ applied to the fluid by the immersed cell is given by:

$$\begin{aligned}\mathbf{g}(\mathbf{x}, t) = &\int_0^L \big[-\mathbf{f}(s,t)\big]\varphi_\varepsilon\big(\mathbf{x}-\mathbf{X}(s,t)\big)ds + \frac{1}{2}\nabla\times\int_0^L \big[-\mathbf{n}(s,t)\big]\varphi_\varepsilon\big(\mathbf{x}-\mathbf{X}(s,t)\big)ds \\ &+\sum_{i=1}^{n_{\mathrm{b}}}\int_{L'}^{L}\big[-\mathbf{f}_i^{\mathrm{r}}(s,t)\big]\varphi_\varepsilon\big(\mathbf{x}-\mathbf{X}(s,t)\big)ds + \sum_{i=1}^{n_{\mathrm{b}}}\Big[\int_{L'}^{L}\mathbf{f}_i^{\mathrm{r}}(s,t)ds\Big]\varphi_\varepsilon\big(\mathbf{x}-\mathbf{X}_i^{\mathrm{b}}(t)\big) \\ &+\sum_{i=1}^{n_{\mathrm{b}}}(-\mathbf{F}_i^{\mathrm{b}}(t))\varphi_\varepsilon(\mathbf{x}-\mathbf{X}_i^{\mathrm{b}}(t)).\end{aligned} \tag{10}$$

The first and second terms on the right-hand side represent the force and torque, respectively, exerted by the flagellum, and the third and fourth terms correspond to a short-range repulsive force that prevents contact between the flagellum and the cell body. Note that the repulsive force is not activated near the motor end of the filament by setting the lower limit L' of the integral to a value greater than zero. The fifth term accounts for the force exerted by the cell body, as discussed above. The bell-shaped blob function φ_ε, with infinite support, is defined as

$$\varphi_\varepsilon(\mathbf{r}) = \frac{15\varepsilon^4}{8\pi(\|\mathbf{r}\|^2+\varepsilon^2)^{7/2}}, \tag{11}$$

where ε is the regularization parameter. The blob function satisfies $\int_{\mathbb{R}^3}\varphi_\varepsilon(\mathbf{r})d\mathbf{r} = 1$, enabling smooth transfer of forces and torques from Lagrangian points of the model cell to the Eulerian fluid domain. The motion of the cell, including the body points $\mathbf{X}_i^{\mathrm{b}}$ and the flagellar configuration $\{\mathbf{X}, \mathbf{D}^1, \mathbf{D}^2, \mathbf{D}^3\}$, is governed by the following equations:

$$\begin{aligned}\frac{\partial\mathbf{X}}{\partial t}(s,t) &= \mathbf{u}\big(\mathbf{X}(s,t),t\big) - \frac{1}{\alpha_1}\Big[\mathbf{f}(s,t) + \sum_{i=1}^{n_{\mathrm{b}}}\mathbf{f}_i^{\mathrm{r}}(s,t)\Big], \\ \frac{\partial\mathbf{X}_i^{\mathrm{b}}}{\partial t}(t) &= \mathbf{u}(\mathbf{X}_i^{\mathrm{b}}(t),t) - \frac{1}{\alpha_2}\Big[\mathbf{F}_i^{\mathrm{b}}(t) - \int_{L'}^{L}\mathbf{f}_i^{\mathrm{r}}(s,t)ds\Big], \qquad i = 1,...,n_{\mathrm{b}}, \\ \frac{\partial\mathbf{D}^j}{\partial t}(s,t) &= \Big[\mathbf{w}\big(\mathbf{X}(s,t),t\big) - \frac{1}{\beta}n_3(s,t)\mathbf{D}^3(s,t)\Big]\times\mathbf{D}^j(s,t), \qquad j = 1,2,3,\end{aligned} \tag{12}$$

where the angular velocity of the fluid is given as $\mathbf{w} = \frac{1}{2}\nabla\times\mathbf{u}(\mathbf{x},t)$. The parameters α_i's and β are translational and rotational drag coefficients, respectively, and $n_3(s,t)$ is the axial component of torque density $\mathbf{n}$.

To create shear flow, we impose a linear shear profile in the y-direction, with the shear velocity field defined as

$$\mathbf{u}_{\mathrm{shear}}(\mathbf{x}) = (0, \gamma z, 0), \tag{13}$$

where $\mathbf{x} = (x, y, z)$ and γ denotes the shear rate. The corresponding angular velocity field associated with this shear flow is given by

$$\mathbf{w}_{\mathrm{shear}}(\mathbf{x}) = \frac{1}{2}\nabla\times\mathbf{u}_{\mathrm{shear}} = (-\frac{\gamma}{2}, 0, 0). \tag{14}$$

Both the translational velocity $\mathbf{u}_{\mathrm{shear}}(\mathbf{x})$ and the angular velocity $\mathbf{w}_{\mathrm{shear}}(\mathbf{x})$ are added directly to the velocities of the immersed cell as specified in Eq. 12.

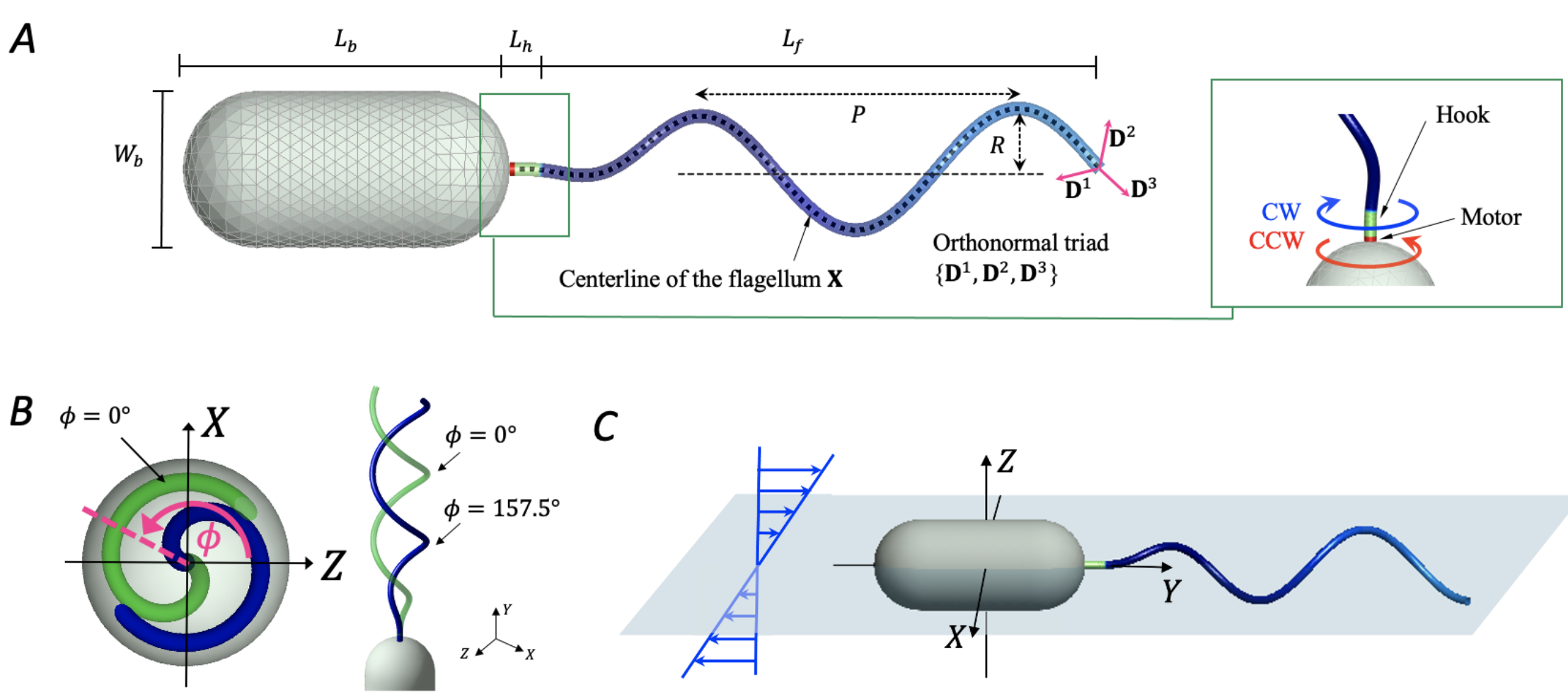

Figure S2: (A) Schematic diagram of the numerical model organism. (B) Definition of the initial phase angle (ϕ) of the flagellum. In the left panel, the initial phase angle ϕ is defined by counterclockwise rotation of the $\phi = 0°$ flagellum (green) about the helical axis. Flagella with initial phase angles of $0°$ (green) and $157.5°$ (navy) are shown in the right panel. (C) Initial setup under shear flow. The dark blue arrows indicate the direction and magnitude of the flow. The light blue panel represents the imaginary focal plane, where the fluid velocity is zero, corresponding to the experimental observation field. In the numerical simulation, flow velocities are computed by subtracting the experimental flow velocity from the measured velocity in the observation field.

Parameter (symbol)	Value
Mesh width for flagellum (Δs)	$0.0303\,\mu\text{m}$
Helical radius of filament (R)	$0.35\,\mu\text{m}$
Helical pitch of filament (P)	$2.0\,\mu\text{m}$
Axial length of helical filament (L_f)	$3.5\,\mu\text{m}$
Curvilinear length of filament (ℓ)	$4.99\,\mu\text{m}$
Intrinsic curvature of filament ($\kappa_1 = \kappa_2$)	$1.5637\,\mu\text{m}^{-1}$
Intrinsic twist of filament (κ_3)	$1.4222\,\mu\text{m}^{-1}$
Bending modulus of filament ($a_1 = a_2 = a$)	$0.003\,\text{g}\mu\text{m}^3\text{s}^{-2}$
Twist modulus of filament (a_3)	$0.003\,\text{g}\mu\text{m}^3\text{s}^{-2}$
Shear modulus ($b_1 = b_2$)	$2\,\text{g}\mu\text{ms}^{-2}$
Stretch modulus (b_3)	$2\,\text{g}\mu\text{ms}^{-2}$
Length of hook (L_h)	$4\Delta s$
Bending modulus of hook ($a_{1,2}^{\text{hook}} = a^{\text{hook}} = a/25$)	$0.0012\,\text{g}\mu\text{m}^3\text{s}^{-2}$
Twist modulus of hook ($a_3^{\text{hook}} = a_3$)	$0.003\,\text{g}\mu\text{m}^3\text{s}^{-2}$
Torque (τ)	2.0 (push), -2.0 (pull), -4.0 (wrapped) nN·nm
Cell body length (L_b)	$2.2\,\mu\text{m}$
Cell body width (W_b)	$0.9\,\mu\text{m}$
Penalty parameter for restoring force (K)	$2.0\,\text{gs}^{-2}$
Number of material points on the cell body (n_b)	770
Fluid viscosity (μ)	$1 \times 10^{-4}\ \text{g}(\mu\text{ms})^{-1}$
Regularization parameter (ε)	$3\Delta s$
Translational drag coefficient (α_1)	$1.3221 \times 10^{-4}\ \text{gs}^{-1}$
Translational drag coefficient (α_2)	$4.0 \times 10^{-6}\ \text{g}\mu\text{ms}^{-1}$
Rotational drag coefficient (β)	$1.3221 \times 10^{-4}\ \text{gs}^{-1}$
Shear rate (γ)	$0 \sim 100\ \text{s}^{-1}$
Time step (Δt)	$2 \times 10^{-8}\ \text{s}$

Table S2: Computational and physical parameters

3 Supplemental experimental results

In this part, we discuss how the orientation of bacterial cells is defined with respect to fluid flow streamlines. Also, we consider the role of motility in the orientation and show the reorientation process of the individual swimming cells.

3.1 Swimmers' velocities and run time

To demonstrate that swimming bacteria do not change their motility in response to local shear, the swimming speeds are measured under flow conditions and in the rest fluid. Velocities are comparable for cell populations at the same distance above the channel surface.

conditions	run time, s		
	push	pull	wrapped
bulk:			
no flow	0.30	0.39	0.56
flow	0.34	0.43	0.54
surface:			
no flow	0.39	0.25	0.34
flow	0.31	0.62	0.49

Table S3: The mean run time in different swimming modes. By italic font, the measurement focal area is specified, where *bulk* is related to $h = 20\,\mu\mathrm{m}$ and *surface* to $h = 5\,\mu\mathrm{m}$ above the bottom surface of the channel.

conditions	swimming velocity, $\mu m/s$		
	push	pull	wrapped
no flow	25.8	16.6	17.2
flow	23.2	17.0	13.4

Table S4: The mean population velocities in different swimming modes, $h = 20\,\mu\mathrm{m}$.

3.2 Cell body orientations and velocity vectors

We define two types of bacterial cell orientation angles: ψ corresponds to the angle between the pointing vector (vector pointed from the center of mass of the flagella to that of the cell body) and the direction of flow (drift velocity). On the other hand, θ corresponds to the angle between the direction of the displacement (by connecting the cell body positions from the previous frame to the current frame) and the direction of flow, see also Figure 1D, in the Main Text. The ψ angles are defined for push and pull modes.

The angles as a function of time for several typical swimmers are shown in Figure S3. As expected, for the push configuration, the values of the ψ and θ angles are almost the same, the cell body is oriented similarly to the direction of the swimming motion. The difference between the direction of motion and the cell body orientation is around 180° for the pull configuration.

Throughout the cell population, the cell displacement direction (ψ angle) can be assigned with cell body orientation (θ angle) with respect to flow. However, for individual tracks, the time evolution of the angles might be slightly different, see Figure S3. For the wrapped swimming mode, the ψ angle is not defined, and we discuss only the θ angle in the Main Text.

3.3 Dynamics of cell reorientation

To show the details of the cells' reorientation process, the θ angles with time are considered for the long swimming episodes, see Figure S4. The distance between single dots corresponds to the velocities of the

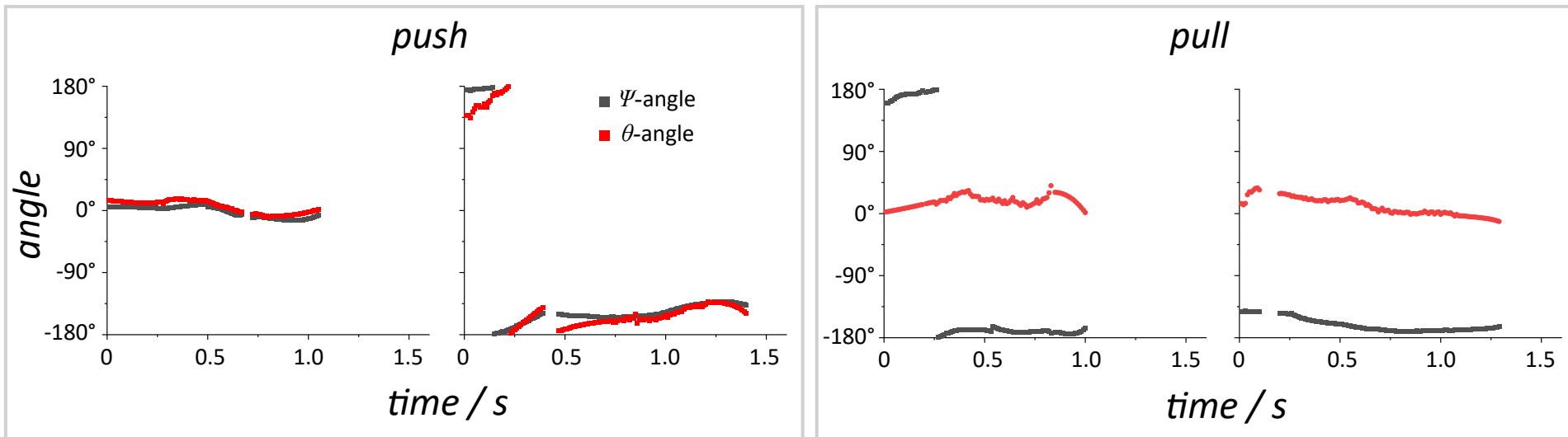

Figure S3: Typical examples of the cells orientation with time. Cell body orientations(ψ angles) and direction of forward swimming (θ angles) with respect to flow, shear rate $1.5s^{-1}$, $h = 20\,\mu\text{m}$. The gaps in the tracking happen due to the loss of one of the signals (red or green) of the swimmer.

angular changes. For the wrapped configuration, the angular velocities are higher in comparison with the unwrapped modes (see Figure S4, heatmaps) and the amplitude of the angular changes along the tracks are bigger (see green and red dots in plots). For the unwrapped modes, the reorientations around 180° and -180° are slower across streamline orientations. The reorientation of the swimming locomotion direction is not persistent. The competition between the alignment and chirality-driven drift-side drift can cause it at the same time as thermal noise.

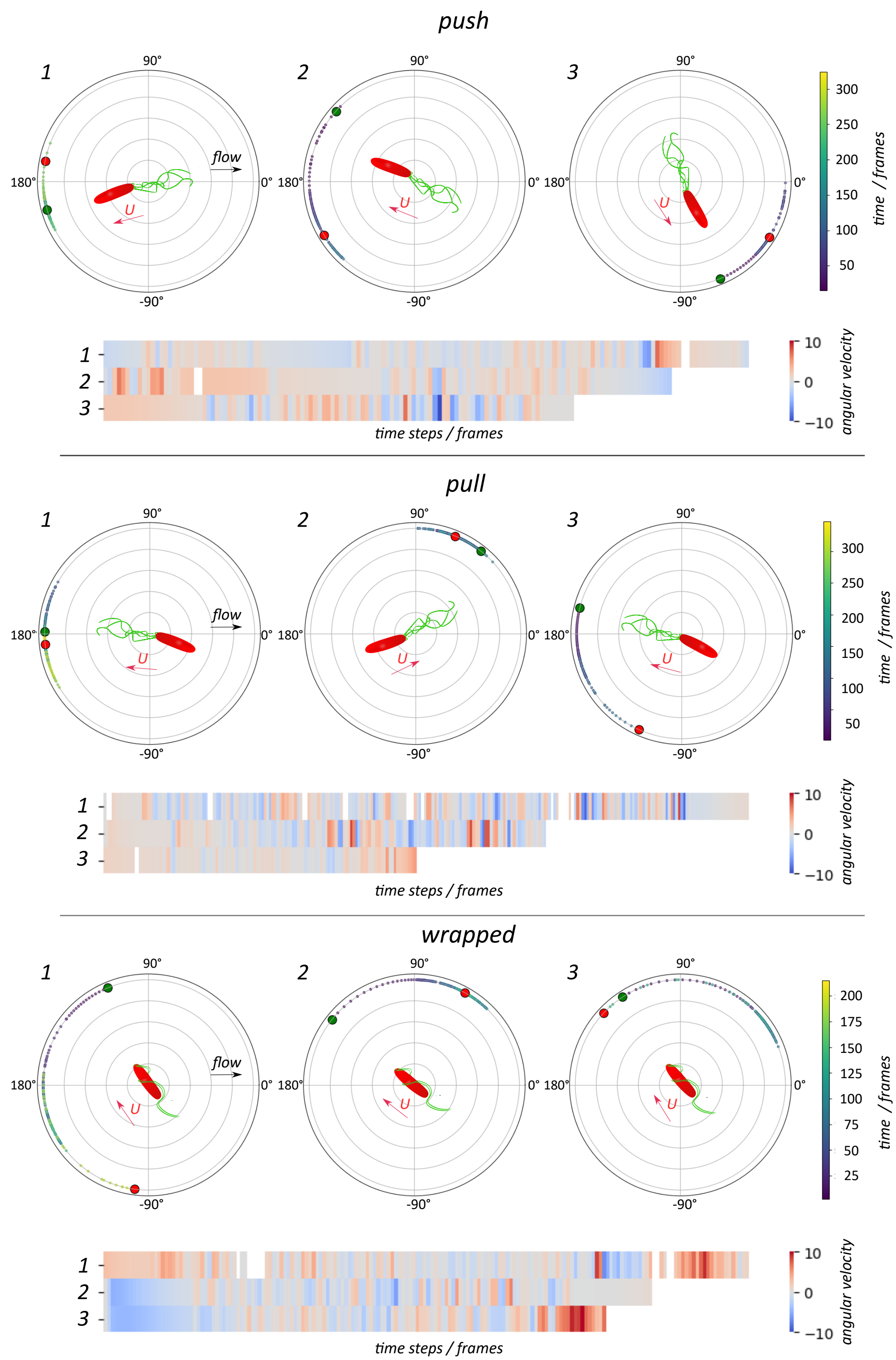

Figure S4: Orientations of velocity vectors (ψ angles) and angular velocities of individual cells in different swimming modes. Results are shown for the typical long episodes in bulk. Colorbar representing the time, time resolution 100 fps, the green dots indicate the angle at the beginning of the episode, the red dots point to the end of it. Heatmaps were shown for the filtered angular velocities between +10° and -10°. Shear rate $1.5s^{-1}$, $h = 20\,\mu\text{m}$.

4 Statistical analysis: Bayesian inference of bacterial alignment

4.1 Parametric inference—maximum-likelihood approach

In Figures. 3, 4B and 5B of the main text, histograms of the cell body orientation (angle ψ) and the swimming direction of bacteria with respect to the flow (angle θ) are shown, respectively[1]. Due to the high persistence of bacterial motion, consecutive orientation vectors during one run episode (push, pull or wrapped) are highly correlated. For small numbers of trajectories, this may introduce unintended biases or statistical artfacts in the histograms, e.g. due to different trajectory lengths. To verify the robustness of the reported results, we present a complementary statistical analysis in this appendix section.

In order to avoid the usage of correlated angle data, we select only one angle φ from each run episode at random; in the following, the variable φ may stand for both, the body orientation ψ or the displacement direction θ, depending on the context. This random selection procedure leads to a reduction of the sample size, such that histograms are not immediately informative. Here, we therefore rather employ a parametric inference approach that does not rely on binned histograms.

As bacteria are expected to align their body axis with respect to the flow [8], we model the distribution of angles $P(\varphi)$ as a mixture of a uniform background and two Gaussian peaks located at 0 and π,

$$P(\varphi|\sigma_1, \sigma_2, p_1, p_2) = p_1 G(\varphi|0, \sigma_1) + p_2 G(\varphi|\pi, \sigma_2) + \frac{1 - p_1 - p_2}{2\pi}, \tag{15}$$

in which

$$G(\varphi|\mu, \sigma) = \frac{1}{2\pi\sigma^2} \sum_{k=-\infty}^{\infty} \exp\left[-\frac{(\varphi - \mu + 2\pi k)^2}{2\sigma^2}\right] \tag{16}$$

abbreviates the probability density function (PDF) of a wrapped Gaussian distribution with location parameter μ and width σ. The parameters p_i determine the relative weight of the two Gaussians with respect to the uniform background. Note that more subtle effects, such as shifts in the peak positions or an asymmetry of the peaks are not taken into account in this analysis, where we focus solely on the overall shear-induced alignment.

Given a sample of N uncorrelated angles φ_j, the likelihood function $L_{\boldsymbol{\varphi}}(\sigma_1, \sigma_2, p_1, p_2)$ is obtained as the product of the probability densities $P(\varphi|\sigma_1, \sigma_2, p_1, p_2)$, evaluated at φ_j:

$$L_{\boldsymbol{\varphi}}(\sigma_1, \sigma_2, p_1, p_2) = \prod_{j=1}^{N} P(\varphi_j|\sigma_1, \sigma_2, p_1, p_2). \tag{17}$$

We inferred the parameter values of the statistical model Eq. (15) by maximizing the likelihood with respect to the parameter values. Figures S5-S7 show the inferred distributions along with the empirical histograms of the underlying sample (for illustration purposes only).

4.2 Quantification of bacterial alignment

Supplemented by suitable priors, the likelihood-based approach outlined above enables us to calculate Bayesian uncertainty bounds. Here, we use uniform priors for the parameters,

$$p(\sigma_1, \sigma_2, p_1, p_2) \propto \Theta(\sigma_1)\,\Theta(\sigma_2)\,\Theta(\pi - \sigma_1)\,\Theta(\pi - \sigma_2)\,\Theta(p_1)\,\Theta(p_2)\,\Theta(1 - p_1 - p_2)\,, \tag{18}$$

in which $\Theta(x)$ denotes the Heaviside-step function. By virtue of Bayes' theorem, the product of likelihood and prior yields the posterior distribution of parameters

$$P(\sigma_1, \sigma_2, p_1, p_2|\varphi_1, \varphi_2, \dots, \varphi_N) \propto L_{\boldsymbol{\varphi}}(\sigma_1, \sigma_2, p_1, p_2) p(\sigma_1, \sigma_2, p_1, p_2) \tag{19}$$

that describes the degree of believe of parameters $\{\sigma_1, \sigma_2, p_1, p_2\}$ given the observed data $\{\varphi_1, \varphi_2, \dots, \varphi_N\}$. It therefore allows to determine uncertainty bounds of parameter values.

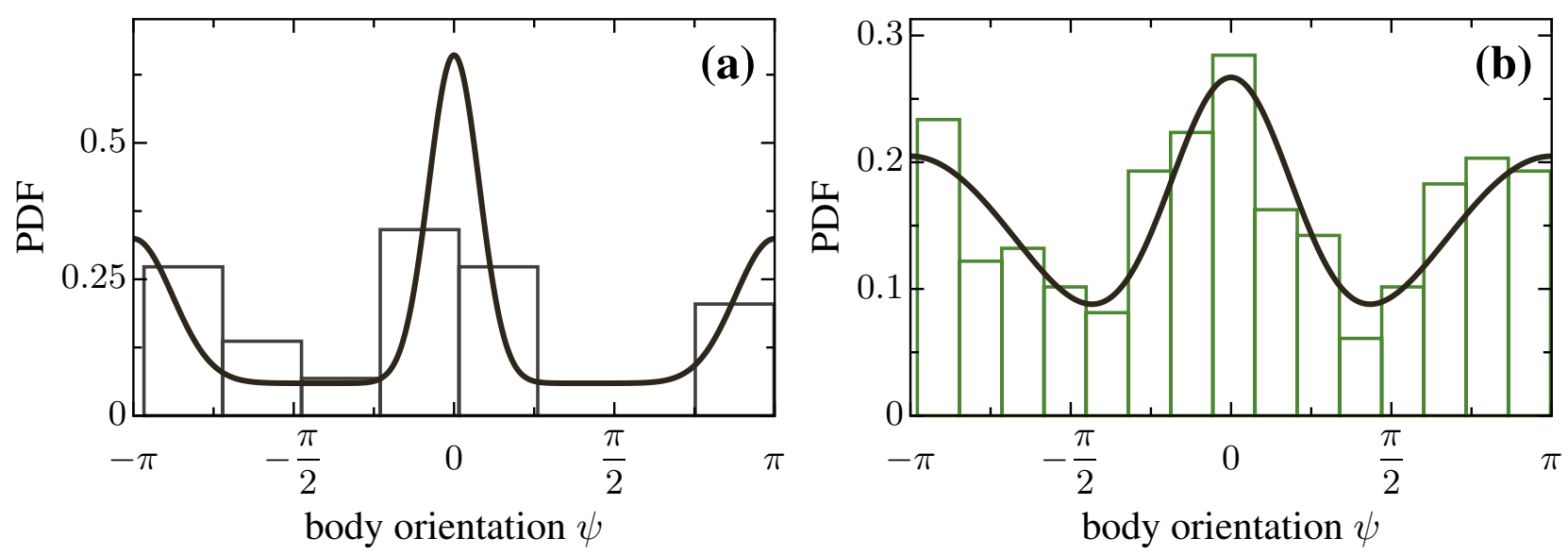

Figure S5: PDF of the body orientation ψ, inferred by likelihood maximization (black line), of (a) non-motile (passive) vs. (b) actively moving bacteria, along with the histograms of the underlying sample (see also Figure 3 of the main text). Sample size: $N = 19$ (passive), $N = 238$ (active).

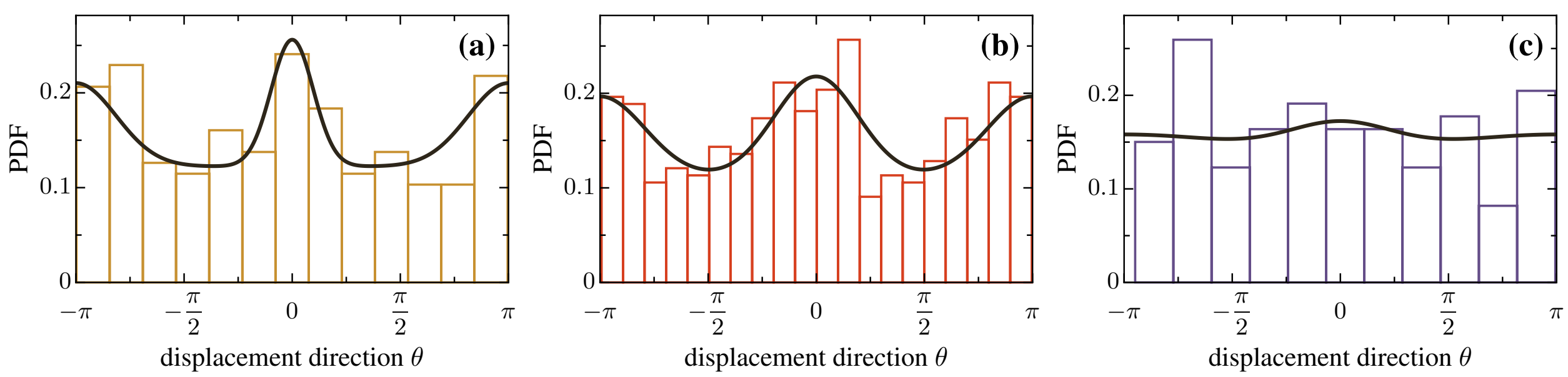

Figure S6: PDF of the displacement orientation θ, inferred by likelihood maximization (black line), for different flow conditions (corresponding to Figure 4 of the main text), along with histograms of the underlying sample. (a) bulk flow ($h = 20\,\mu\mathrm{m}$) with shear rate $\gamma_1 = 1.5\,\mathrm{s}^{-1}$, (b) bulk flow ($h = 20\,\mu\mathrm{m}$) with shear rate $\gamma_1 = 3\,\mathrm{s}^{-1}$, (c) data taken close to a surface ($h = 5\,\mu\mathrm{m}$) with shear rate γ_1. Sample size: $N = 181$ (a), $N = 424$ (b), $N = 132$ (c).

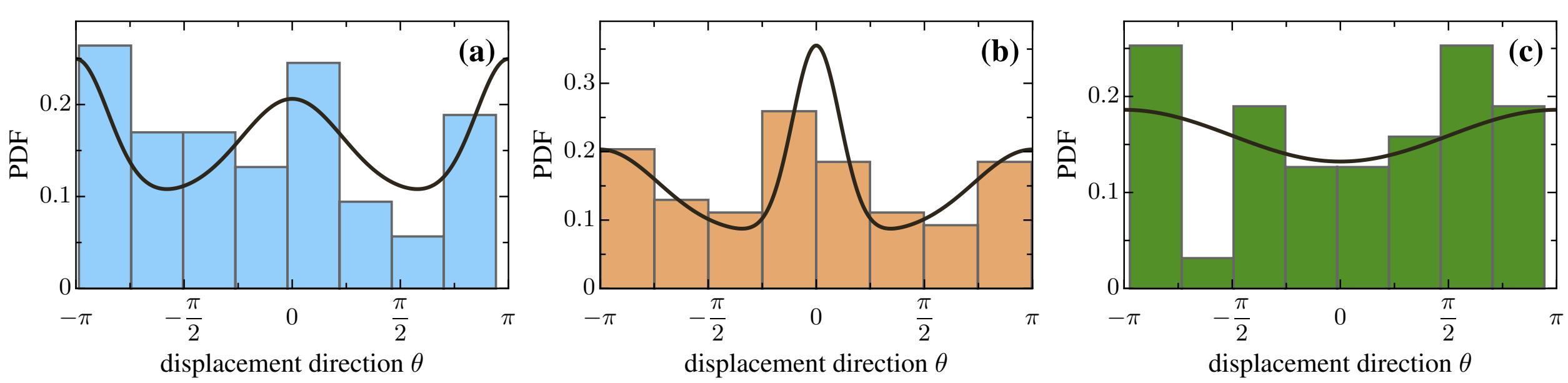

Figure S7: PDF of the displacement orientation θ, inferred by likelihood maximization (black line), for different swim modes corresponding to Figure 5 of the main text (a: push, b: pull, c: wrapped), along with histograms of the underlying sample. Data taken $h = 20\,\mu\mathrm{m}$ above the surface at a shear rate of $\gamma_1 = 1.5\,\mathrm{s}^{-1}$. Sample size: $N = 70$ (a: push), $N = 69$ (b: pull), $N = 42$ (c: wrapped).

In the present context, we are not interested in the parameter values themselves but rather in the relative strength of bacterial alignment under different flow conditions and of different swim modes, respectively—strong alignment is reflected by a strongly peaked distribution, whereas weak alignment results in a uniform PDF of φ angles. In order to quantify how strongly an inferred distribution deviates from the uniform

[1] The swimming direction θ was obtained from the velocity of bacteria after subtraction of the fluid velocity; the angle θ is a proxy for the direction of self-propulsion and, thus, for the body axis orientation of bacteria.

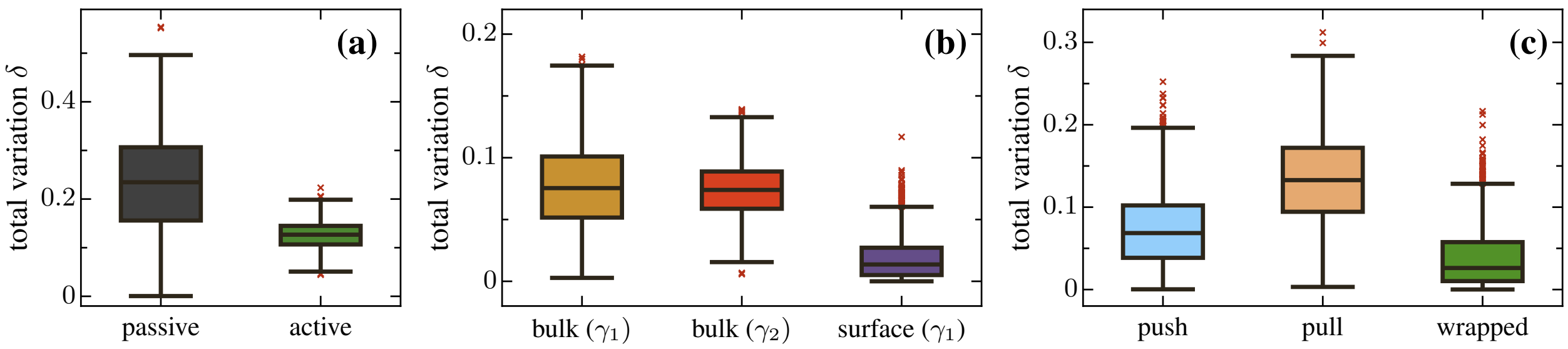

Figure S8: The box plots show the total variation δ, cf. Eq. (20), quantifying the deviation of an inferred distribution from a uniform distribution ($\delta = 0$ corresponds to the uniform distribution, $\delta = 1$ to an infinitely sharply peaked PDF). The sample of δ-values was obtained by sampling from the posterior PDF $P(\sigma_1, \sigma_2, p_1, p_2|\varphi_1, \varphi_2, \ldots, \varphi_N)$ Eq. (19). Sample size in all cases: $N = 10^3$. The data in panel (a) correspond to the data in Figure S5, (b) corresponds to Figure S6 and (c) corresponds to Figure S7.

distribution, we use the total deviation

$$\delta(\sigma_1, \sigma_2, p_1, p_2) = \frac{1}{2} \left| \int_{-\pi}^{\pi} d\varphi \, P(\varphi|\sigma_1, \sigma_2, p_1, p_2) - \frac{1}{2\pi} \right|. \tag{20}$$

It is generally used as a measure of similarity of probability distributions: the total variation δ equals zero if the inferred distribution $P(\varphi|\sigma_1, \sigma_2, p_1, p_2)$ is equal to the uniform density, and one for infinitely sharp peaks ($\sigma_{1,2} \to 0$).

We sampled 10^3 parameter combinations from the posterior $P(\sigma_1, \sigma_2, p_1, p_2|\varphi_1, \varphi_2, \ldots, \varphi_N)$, Eq. (19), applying Metropolis–Hastings algorithm, and calculated the total variation each time. The resulting distributions of δ-values are shown as box plots in Figure S8. In Figure S8a, the alignment of non-motile bacteria are compared to actively moving bacteria (cf. Figure 3 of the main text); the alignment in the latter case is reduced due to changes of swim modes which are accompanied by a randomization of the direction of motion. Figure S8b reveals that bacterial alignment is significantly reduced close to surfaces in comparison to the alignment in bulk at comparable shear rates (cf. Figure 4B of the main text). In addition, Figure S8c shows that bacteria in the wrapped swimming mode tend to be less aligned as compared to bacteria in the push and pull mode (see also Figure 5B of the main text).

5 Comparison of simulation results with the experiment

In this part, we numerically reproduced the experimental orientation histograms displayed in Figure 5 of the main text. Specifically, we simulated larger numbers of cells swimming in push, pull, and wrapped mode (28 cells in each mode) in the shear flow, starting from different initial body orientations and with different initial flagella phases. From this new data set, we computed directional histograms, similar to the experimental ones shown in Figure 5, and could successfully reproduce the different rheotactic responses of pushers and pullers. The direction of the y-axis in Figure S9 corresponds to the flow direction.

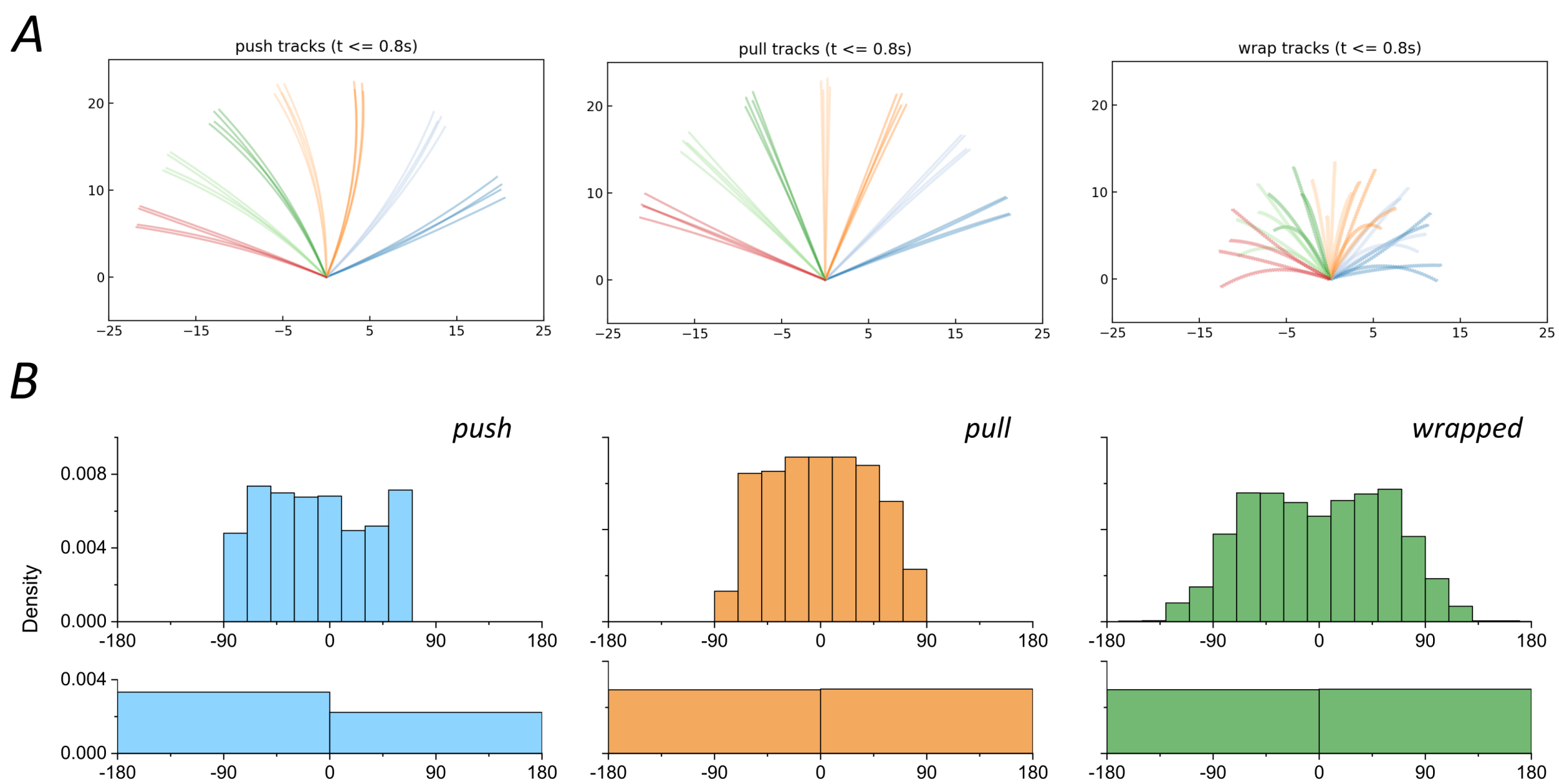

Figure S9: Numerical simulations: trajectories of bacteria in a shear flow. Trajectories of swimmers in push, pull, and wrapped modes with different initial body orientations under shear flow conditions. (A) Top views are shown for each swimming mode. In each view, 28 trajectories, corresponding to 7 different initial cell body orientations and 4 different initial flagella phases, are displayed for each swimming mode. Each color represents a different body tilting angle ($22.5° \times 0, \pm1, \pm2, \pm3$) with respect to the flow direction. Different trajectories with the same color correspond to different initial flagellar phase angles ($\phi = 0°, 90°, 180°, 270°$). Each simulation is run for 0.8 s with a shear rate of $\gamma = 3.0\ s^{-1}$. The torque values for each mode are as follows: $\tau = 2.0$ nN·nm for push, $\tau = -2.0$ nN·nm for pull, and $\tau = -4.0$ nN·nm for the wrapped mode. (B) Corresponding distributions of θ angles, separated according to swimming modes, the time resolution of the trajectories used to compute the histograms is $\Delta T = 0.05$ s.

References

[1] V. Pfeifer, V. Muraveva, and C. Beta, Flagella and Cell Body Staining of Bacteria with Fluorescent Dyes, Cell Motility and Chemotaxis: Methods and Protocols, 79-85, (2024)

[2] M. Hintsche, V. Waljor, R. Großmann, M.J. Kühn, K.M. Thormann, F. Peruani, and C. Beta, A polar bundle of flagella can drive bacterial swimming by pushing, pulling, or coiling around the cell body, Scientific Reports 7, 16771 (2017)

[3] A. Datta, TrackScoPy: A Python toolbox for tracking and analyzing bacterial motility from fluorescence microscopy videos, Zenodo [doi:10.5281/zenodo.17456571] (2025)

[4] M. Theves, J. Taktikos, V. Zaburdaev, H. Stark, and C. Beta, A Bacterial Swimmer with Two Alternating Speeds of Propagation, Biophysical Journal, 105(8), 1915 (2013)

[5] J. Crocker and D. Grier, Methods of Digital Video Microscopy for Colloidal Studies, Journal of Colloid and Interface Science, 179(1), 289 (1996)

[6] S. Olson, S. Lim, and R. Cortez, Modeling the dynamics of an elastic rod with intrinsic curvature and twist using a regularized Stokes formulation, Journal of Computational Physics, 238, 169 (2013)

[7] W. Lee, Y. Kim, C. S. Peskin, and S. Lim, A novel computational approach to simulate microswimmers propelled by bacterial flagella, Physics of Fluids 33, 111903 (2021)

[8] G. Jing, A. Zöttl, É. Clement, and A. Lindner, Chirality-induced bacterial rheotaxis in bulk shear flows, Science Advances 6, eabb2012 (2020)
