# Supplementary material for: Swimming patterns of a multi-mode bacterial swimmer in fluid shear flow
Source: Biophys J. 2026 May 30;125(14):3547–57. doi: 10.1016/j.bpj.2026.05.032 (PMC13507449; doi:10.1016/j.bpj.2026.05.032)
Supplement: Supplementary file 2 [file mmc8.pdf]

Biophysical Journal
Article

Biophysical Society

Swimming patterns of a multi-mode bacterial swimmer in fluid shear flow

Valeriia Fast,[1] Agniva Datta,[1] Jeungeun Park,[2] Robert Großmann,[1] Veronika Pfeifer,[1] Yongsam Kim,[3] Wanho Lee,[4] Sookkyung Lim,[5] and Carsten Beta[1,6,*]

[1]Institute of Physics and Astronomy, University of Potsdam, 14476 Potsdam, Germany; [2]Department of Mathematics, State University of New York at New Paltz, New Paltz, NY 12561, USA; [3]Department of Mathematics, Chung-Ang University, Seoul 06974, South Korea; [4]National Institute for Mathematical Sciences, Daejeon 34047, South Korea; [5]Department of Mathematical Sciences, University of Cincinnati, Cincinnati, OH 45221, USA; and [6]Nano Life Science Institute (WPI-NanoLSI), Kanazawa University, Kanazawa 920-1192, Japan

ABSTRACT Bacterial swimming is well characterized in uniform liquids at rest. The natural habitat of bacterial swimmers, however, is often dominated by moving fluids and interfaces, resulting in shear flows that may strongly alter bacterial navigation strategies. Here, we study how fluid shear flow affects the swimming motility of the soil bacterium *Pseudomonas putida*, a bacterial swimmer that moves in a versatile pattern composed of three different swimming modes, where the flagella may push, pull, or wrap around the cell body (multi-mode swimmer). We introduce a computer automated cell tracking and swimming mode detection tool to show that shear-induced alignment depends on the swimming mode, while motility and proximity to surfaces counteract the alignment effect. Moreover, filament wrapping becomes less efficient with increasing shear stress. Numerical simulations of realistic swimmer geometries complement our experimental results, providing more detailed mechanistic insights into movement patterns of bacterial swimmers in a shear flow.

SIGNIFICANCE When colonizing plant roots in the soil or during spreading of infections in the human body, bacteria propel themselves through moving fluids. This work explores how fluid shear flow influences the swimming pattern of *Pseudomonas putida*, a soil bacterium that moves in three alternating swimming modes, where flagella push, pull, or wrap around the cell body. Using dual-color fluorescence microscopy together with semi-automated image analysis, we reveal that shear-induced alignment depends on the swimming mode. Moreover, increased shear stress stabilizes the pulling mode by reducing the efficiency of filament wrapping. Numerical simulations support our experimental findings, providing insights into bacterial movement patterns under flow conditions and emphasizing how distinct types of swimmers differ in their response to shear flow.

INTRODUCTION

The locomotion of biological microswimmers in complex environments is central to many medical functions, such as the spreading of infections[1,2] or sperm navigation inside the female reproductive tract.[3,4] For bacteria, one of the largest classes of biological microswimmers, studies in uniform liquid environments have shown that they control their direction of locomotion by changing the mode of operation of their helical flagella. *Escherichia coli* (*E. coli*), the most widely studied example, carries flagella distributed all over its cell body (peritrichous flagellation) and moves in a run-and-tumble pattern.[5] Here, counterclockwise (CCW) rotation of the flagella results in the formation of a coherent bundle that pushes the cell body forward (run). Persistent run episodes are interrupted by erratic turn events (tumbles) that are induced by one or several motors switching to clockwise (CW) rotation, driving the coherent bundle apart and resulting in a random reorientation of the cell body.[6]

Apart from the well-studied run-and-tumble motility of peritrichously flagellated *E. coli*, more complex swimming patterns have been reported for bacteria that exhibit other flagellation architectures.[7] While *E. coli* always swims as a pusher, other species may exhibit combinations of different run modes (multi-mode swimming), such as in the run-reverse-flick pattern of monotrichously flagellated species.[8] Here, we focus on the soil bacterium *Pseudomonas putida* (*P. putida*) as an example of a multi-mode bacterial

Submitted October 31, 2025, and accepted for publication May 21, 2026.
*Correspondence: beta@uni-potsdam.de
Editor: N.G. Cogan.
https://doi.org/10.1016/j.bpj.2026.05.032
© 2026 The Author(s). Published by Elsevier Inc. on behalf of Biophysical Society.
This is an open access article under the CC BY license (http://creativecommons.org/licenses/by/4.0/).

swimmer that has been intensely studied both in open uniform liquid[9] and in confined and complex environmental geometries.[10–13] *P. putida* carries a tuft of several helical flagella located at one cell pole (lophotrichous flagellation).[14] It may push itself forward by CCW rotation of its flagellar bundle, while CW operation of the flagellar motor pulls the cell body in the opposite direction. Additionally, *P. putida* can wrap its bundle of flagella around the cell body to move in a screw thread fashion (wrapped mode),[15] a swimming mode that has also been reported for other polarly flagellated species.[16–18] Wrapped mode formation is promoted by increasing the viscosity of the surrounding medium[16,19] and plays a key role for chemotaxis of *P. putida* in nutrient gradients.[20] Nevertheless, the functional relevance of filament wrapping and the general benefits of switching between different swimming modes are not fully understood and remain a matter of current debate.[21]

In many natural habitats of bacterial swimmers, fluid flows are commonly observed, such as blood flow in the vascular system or groundwater flows in a granular soil environment.[1,22,23] Close to surfaces, the velocity gradients of the resulting shear flows will affect the direction of bacterial locomotion. According to the pioneering work by Jeffery, a passive elongated object in a linear shear flow will perform periodic revolutions, following so-called *Jeffery's orbits*.[24] The study of shear flow effects on particle dynamics was later extended by Bretherton and others.[25,26] For active particles in a Poiseuille flow, two types of trajectory patterns, "tumbling" and "swinging," defined by initial conditions and particle shape, were observed in experiments[27] and described theoretically.[28] Later, also rheotaxis in fluid shear flows, the directed drift of nonmotile and motile chiral objects across streamlines, was reported[29–33] and complemented by mathematical models.[34,35] For elongated swimmers with chiral filaments, the local shear-induced alignment with the flow competes with the chirality-induced side drift.[30,35] Also close to a surface, the combination of shear flow and other hydrodynamic effects leads to the upstream motion of microswimmers.[30,31,36] All of the above demonstrates that complex environmental flow conditions may strongly affect the spreading of a population of bacterial microswimmers.

In this work, we examine how the swimming behavior of a multi-mode bacterial swimmer is altered under flow conditions. We focus on *P. putida* and report experimental and theoretical results on the swimming pattern of this multi-mode swimmer in bulk and near a solid surface under local shear flow. To examine the runs in each swimming mode separately, dual-color fluorescent microscopy recordings were combined with semi-automatic tracking and swimming mode detection based on a dedicated software tool. On the theoretical side, these results were complemented with mathematical modeling of a realistic swimmer geometry. The model relied on Kirchhoff rod theory to describe the flagellar filaments and their hooks, rigid body dynamics to capture the motion of the cell body, and a generalization of the regularized Stokeslet method to account for their hydrodynamic interactions.[37–39] In this way, we can evaluate the pros and cons of each swimming configuration. Furthermore, our study will provide the basis for future predictions of the spreading efficiency of biological and novel artificial microswimmers under flow conditions.

MATERIALS AND METHODS

Cell culture and staining

We used the bacterial strain *Pseudomonas putida* KT 2440 $FliC_{S267C}$ (later in the text referred to as "wild type"), where the protein FliC was genetically modified for fluorescent staining of the flagella bundle. These bacteria perform run-and-reverse motion in three swimming modes (push, pull, and wrapped; see Figure 1).[15] To investigate the influence of the active propulsion, we tested a nonmotile strain for comparison. In this strain, the torque-generating stators MotAB and MotCD of the bacterial flagella were knocked out. The *P. putida* KT2440 $FliC_{S267C}$ ΔmotAB ΔmotCD double knockout mutant was generated by sequential double homologous recombination as described elsewhere.[19]

A day before the experiment, a single colony was harvested from an LB-agar plate and transformed in 25 mL of tryptone broth media (10 g/L tryptone [AppliChem], 5 g/L NaCl). The solution was placed in an incubator at 30°C and shaken at 300 rpm for 14 h (up to a cell density of $OD_{600} = 0.4$). Later, washing with motility buffer (11.2 g/L K_2HPO_4, 4.8 g/L KH_2PO_4, 3.93 g/L NaCl, 0.029 g/L EDTA, and 5 g/L glucose), the fluorescent staining and further filtration of aggregates were done as described in Pfeifer et al.[40] In short, a washed concentrated bacterial suspension was incubated with Alexa Fluor 488 C5-maleimide (Invitrogen, Thermo Fisher Scientific) over 30 min at room temperature for staining of the flagella. After that, the suspension was washed and re-incubated with the red membrane dye FM4-64 (Invitrogen, Thermo Fisher Scientific) to visualize the cell body. During the last step of staining, the bacterial suspension was gently filtered to remove aggregates of cells that appeared during intermediate centrifugation and washing steps. Finally, the cell suspension was resuspended to $OD_{600} \sim 0.2$ (low-density suspension of cells, to avoid interactions of cells) in motility buffer, which forced the bacteria to stop growing but kept them motile.

Microfluidic setup and image acquisition

The suspension of fluorescently stained cells was injected with a syringe into a rectangular polymer channel of type μ-Slide 0.1 VI, ibiTreat (Ibidi), with height 100 μm (the setup is shown in Figure 1A). For experiments under flow conditions, the bacterial suspension was pushed through

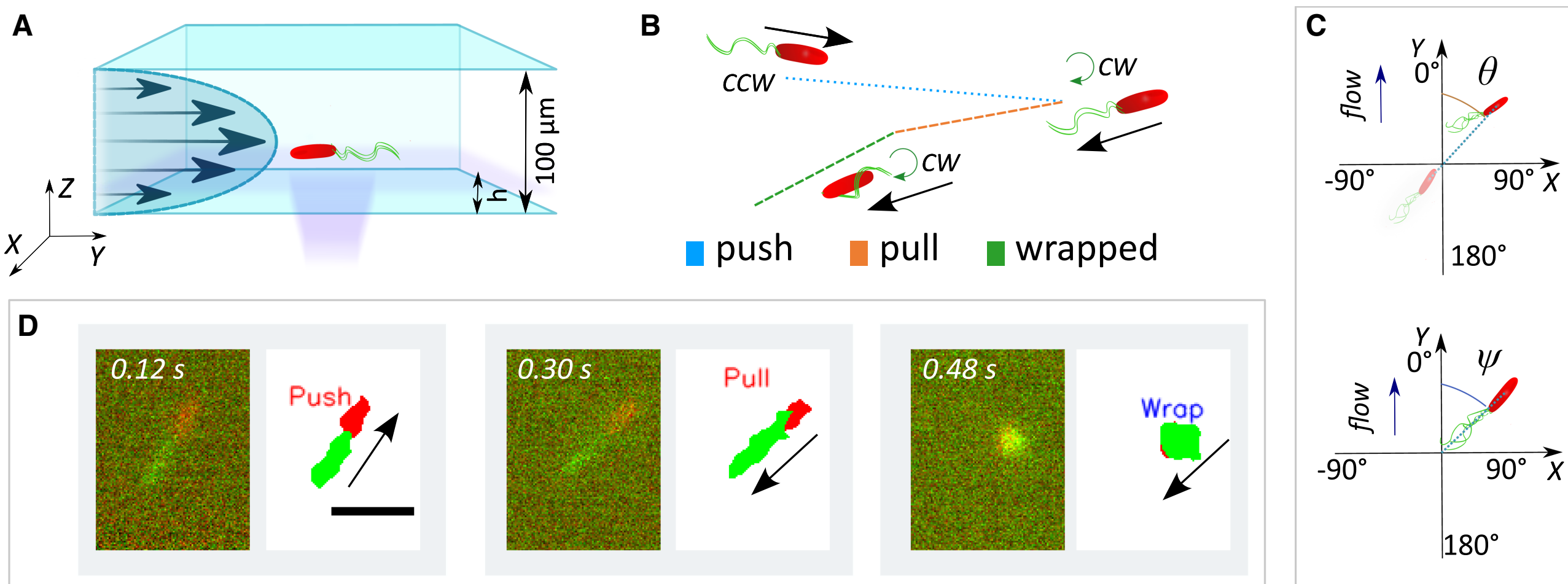

Figure 1. Experimental setup. (A) Bacterial suspension in Poiseuille flow in a microfluidic channel. Recordings are taken at a distance h above the surface. (B) Swimming modes of *P. putida*. (C) Angles in the x-y plane: θ is defined as the orientation of the velocity vector, ψ is the orientation of the cell body, i.e., the vector pointing from the center of mass of the flagellar bundle to the center of the cell body. (D) Examples of raw recording data and results of semi-automatic tracking (see the corresponding Videos S1 and S2 in the supplemental information). The arrows show the direction of the velocity vector in the co-moving frame; scale bar 5 µm.

the channel with a constant flow rate by a syringe pump (Harvard Apparatus, 11 Elite). To achieve a stationary state in the system, the pump was running 20 min before the first measurements. For the bulk experiments, the flow rates were 250 nL/min or 500 nL/min, resulting in local shear rates of $\gamma = 1.5\ s^{-1}$ or $\gamma = 3.0\ s^{-1}$, respectively, at a distance of $h =$ 20 µm above the surface. For h = 5 µm above the surface, the flow rate was decreased to 125 nL/min to maintain a shear rate of $\gamma = 3.0\ s^{-1}$. The experiments with nonmotile mutant cells were carried out at $h = 20$ µm and under a local shear rate of $\gamma = 3.0\ s^{-1}$. The measurements were run 40 times (over 4 days) for the height above bottom surface $h = 20$ µm and shear rate $\gamma = 3.0\ s^{-1}$, 32 times (over 3 days) for $h = 20$ µm and $\gamma = 1.5\ s^{-1}$, 23 times (over 3 days) for $h = 20$ µm in the absence of flow, 10 times (on one day) for $h = 5$ µm and $\gamma = 3.0\ s^{-1}$, and 5 times (on one day) for the case of passive cells. On new measurement days, a fresh cell culture was used.

For fluorescence imaging, an inverted microscope (Olympus, IX71) was equipped with a monochromatic blue LED source with $\lambda = 470$ nm (Prizmatix, UHP-T-LED-470). For splitting the red signal of the cell body and the green signal of the flagella, the splitting optics (Hamamatsu, W-View Gemini) was used. The recordings were taken with a CMOS high-speed camera (Hamamatsu, ORCA-Fusion BT Digital CMOS camera, C15440-20UP) in a fixed focal plane at distances of $h = 5$ µm, or 20 µm above the bottom of the microfluidic channel (see Figure 1A). Images were acquired with a time resolution of 100 frames per second, using a 60x UPLFLN-PH objective (Olympus) with a small depth of field (DP = 0.7 µm) to obtain information from a thin layer of the sample.

Image processing and cell tracking

To track and distinguish cells in different swimming modes, bacteria were stained with fluorescent dyes (red for the body and green for the flagella; see paragraph on "cell culture and staining" above and Figure 1B). Preliminary processing of the fluorescence recordings (channel splitting and frame cropping) was done with the freely available ImageJ software as described earlier.[15] For further tracking and analysis, a custom-made Python code was used[41] (see also supplemental information).

The two channels corresponding to the fluorescence recordings of the red and green signals were segmented and tracked separately; see the workflow scheme in Figure 2. For every position in a trajectory in the red channel (associated with the center of mass of the cell body), the position of the corresponding trajectory in the green channel (associated with the center of mass of the flagellar bundle) of the same bacterium was determined based on a search radius, which was taken to be comparable to the size of the bacterium. The two points were then connected by a vector.

To subtract the flow velocity, separate recordings with only nonmotile cells were used for each measurement condition. The speed of well-resolved inactive cells was averaged and subtracted from the velocities of the cell bodies of the active swimmers obtained from the analysis of trajectories in the red channel. To distinguish between the swimming modes, the orientation of the vector pointing from the center of mass of the flagella to the center of the cell body with respect to the direction of cell displacement was analyzed; see Figure 1C. If the vector connecting flagella and cell body was pointing in the same direction as the displacement vector (after subtracting the fluid flow speed), the cell was assigned to be a pusher (see Figure S1; Table S1 in supplemental

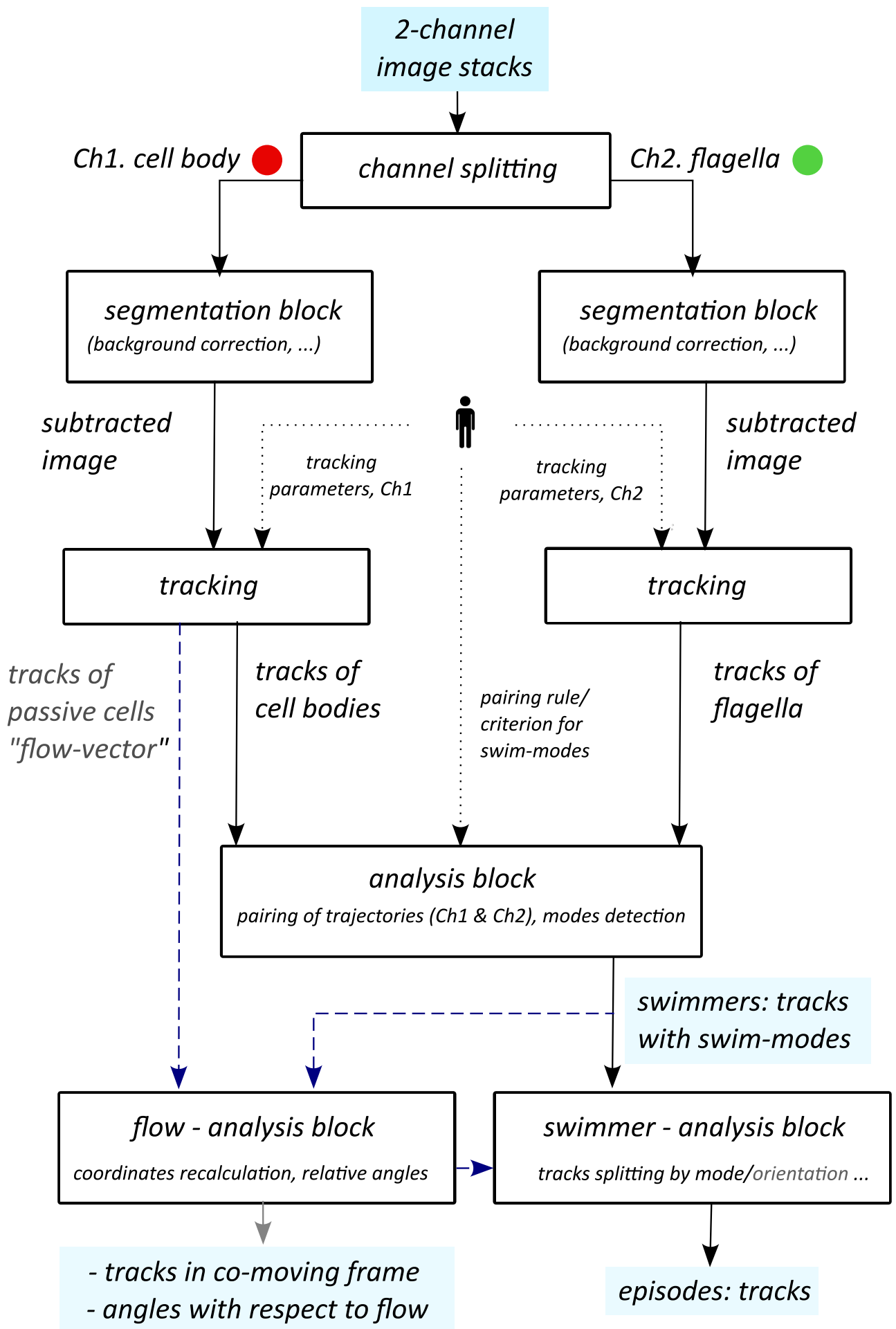

Figure 2. Diagram of the imaging analysis workflow. In the analysis block, the tracks of swimmers are considered as detected from the positions of the swimmers' cell bodies unless otherwise stated. With the blue dashed arrows, the steps required for the flow subtraction are shown. We use the human icon and dotted arrows to mark the parameters that a user must specify manually.

information). On the other hand, a puller was identified when these vectors were oriented in opposite directions. Finally, if the distance between the centers of mass of the cell body and the flagellar bundle fell below a threshold of 1.1 μm, the swimmer was assigned to move in wrapped mode (see also Table S1 in supplemental information). Prior to the swimming mode detection, trajectories were split into individual run episodes, for which the swimming modes were then identified separately; see also Figure 1B.

Note that this analysis relies on two types of orientation angles (see Figure 1C). The angle ψ characterizes the orientation of the cell body and is defined as the angle between the vector, which connects the centers of mass of the flagellar bundle and the cell body, and the direction of the fluid flow velocity, determined from the drift direction of nonmotile cells. The angle ψ can be determined for both pushers and pullers, but not for wrapped swimmers, due to the overlap of a cell body and a flagellar bundle. Also, for the passive nonmotile mutant cells, ψ can be determined. On the other hand, the angle θ represents the orientation of the swimming velocity vector with respect to the direction of fluid flow in the co-moving frame. The velocity vector is determined by connecting the cell body positions in adjacent time frames; see also Figure 1C and Figure S3 in supplemental information. The cell's center of mass positions were detected for the time step $\Delta T = 0.05$ s to determine the bacteria displacement vector and calculation of angle θ. To quantify differences between the angular histograms that were obtained under different flow conditions, a detailed statistical analysis was performed; see supplemental information.

Mathematical framework

To simulate the hydrodynamics of a swimming bacterial cell in a viscous fluid, we employ the regularized Stokeslet formulation coupled with Kirchhoff rod theory, implemented within the immersed boundary framework, which is well suited for fluid-structure interaction problems.[38,42] The cell body is modeled as a neutrally buoyant, rigid body, and the flagellum is treated as an elastic slender filament governed by Kirchhoff rod theory (see Figure S2; Table S2 in supplemental information). The flagellum is attached to the pole of the cell body to complete the full cell model, and this cell is immersed in an incompressible Newtonian fluid. The bacterial flagellar motor generates torque that drives the rotation of the flagellum. Simultaneously, an equal and opposite torque is applied to the cell body, resulting in its counterrotation. The swimming motion is calculated by enforcing force-free and torque-free conditions on the surrounding fluid. This framework enables the accurate capture of both direct mechanical interactions and fluid-structure coupling between the cell body and its motor-driven, flexible flagella. See supplemental information for a detailed mathematical description.

RESULTS

Switches between swimming modes counteract alignment of bacteria in shear flow

It is well known that the orientation of elongated objects in a moving fluid is affected by shear flow.[2,24,26,43,44] Here, we analyze the impact of shear flow on the alignment of motile *P. putida* cells. We first elucidated the overall impact of motility on the alignment of rod-shaped, lophotrichously flagellated bacterial cells in a shear flow. We compared the cell body orientations (ψ angles) of motile *P. putida* wild-type cells with nonmotile *P. putida* mutant cells. A Δ*motAB* Δ*motCD* double mutant, deficient in both stators of the flagellar motor, was used as the nonmotile reference case, as these cells still carry flagellar filaments, but are unable to rotate their flagellar motors.[19] For both wild-type and nonmotile cells, alignment with the flow direction was observed, indicated by peaks at 0° and 180° in the

orientation angle ψ histograms displayed in Figure 3. However, as can be seen from the heights of the peaks in the histogram, the alignment effect was weaker for the motile wild type compared with the nonmotile mutant cells, where the alignment peaks were more pronounced.

Shear-induced alignment of swimming trajectories is reduced close to surfaces

Even though switching between swimming modes compromises alignment in a shear flow, a clear preference to orient with the flow direction was observed. This was also reflected at the level of cell trajectories. In Figure 4A, the swimming trajectories of *P. putida* wild-type cells are shown for two different shear rates in the bulk fluid (at a distance of 20 μm away from the bottom of the microchannel) and in a shear flow close to a surface (at a distance of 5 μm from the bottom of the microchannel). The trajectories are displayed in a co-moving reference frame (i.e., the speed of fluid flow in the focal plane was subtracted), and their starting positions were centered at the origin.

For the two bulk fluid cases (left and middle), the pattern of trajectories is extended in flow direction, indicating an alignment of the direction of motion with the fluid flow. This is also reflected in the histogram of displacement angles θ shown in Figure 4B. For both shear rates, similar peaks at 0° and 180° indicate a preferred swimming direction with or against the fluid flow. In contrast, alignment vanished in the vicinity of the surface; see Figure 4, right-hand side. Here, the orientations of trajectories are more isotropic, and no clear peaks can be distinguished in the histogram of the displacement angle. Note that correlations in the data, resulting from the high degree of persistence during run phases, may lead to additional fluctuations in the histograms. For an in-depth statistical analysis that takes these effects into account and quantifies the differences between the histograms, see supplemental information.

Pushers and pullers align more strongly than swimmers in wrapped mode

In a fluid shear flow away from solid boundaries, we observed an overall alignment of swimming trajectories with the flow

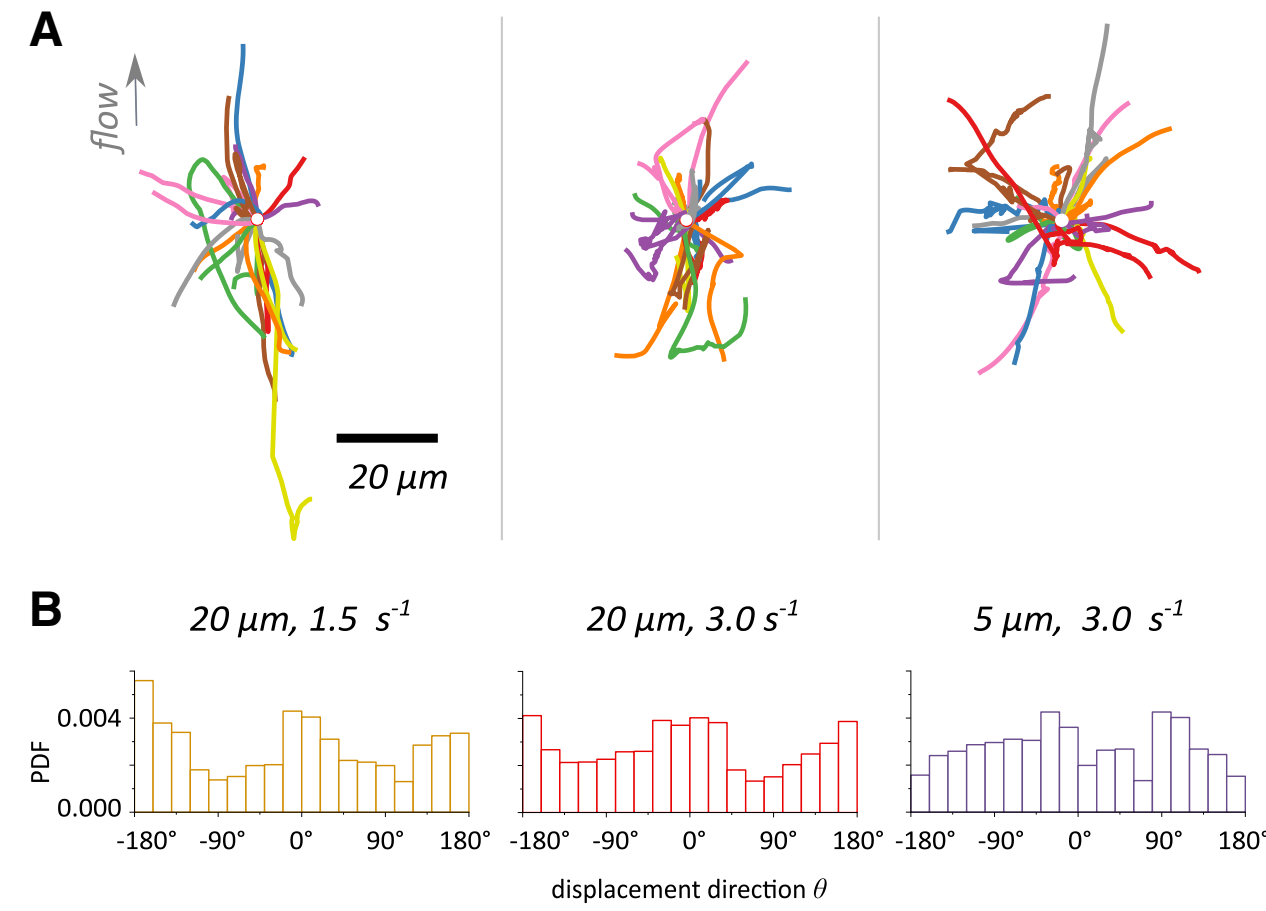

Figure 4. Alignment of bacterial locomotion for different shear rates $\gamma = 1.5\ s^{-1}$ and $3.0\ s^{-1}$, and different distances $h = 20$ μm and 5 μm from the surface. (A) Typical swimmers' trajectories in the co-moving frame, where the 25 longest tracks are shown. Flow speed is subtracted, flow direction is parallel with the Y axis, and the beginning of the tracks is marked by a white circle. (B) Distribution of θ angles (swimming direction) with respect to the flow in bulk ($h = 20$ μm) and near the surface ($h = 5$ μm). The time lag is $\Delta T = 0.05$ s. Numbers of cells: $n_1 = 188$ (left), $n_2 = 462$ (middle), and $n_3 = 144$ (right). The number of data points are 1,385 (left), 2,209 (middle), and 1,081 (right). For statistical analysis and comparison of the histograms, see supplemental information.

direction that breaks down close to a solid boundary. In order to find out to what extent alignment depends on the swimming mode, we performed dual-color fluorescence imaging experiments allowing us to identify for each run, whether the respective cells move in push, pull, or wrapped mode (relying on our semi-automated tracking and assignment software; see Figures 1D and 2 and materials and methods). Based on the assignments of runs to the different swimming modes, we quantified the orientations of cell displacements in the co-moving frame relative to the flow direction for each swimming mode separately. We classified a displacement as being aligned with the flow if its orientation deviated from the flow by less than 45° (with or against the flow direction; see schematic in Figure 5A). The bar plots in Figure 5A display the percentages of displacements aligned in flow direction for each swimming mode separately. The three different flow configurations correspond to the cases shown in Figure 4.

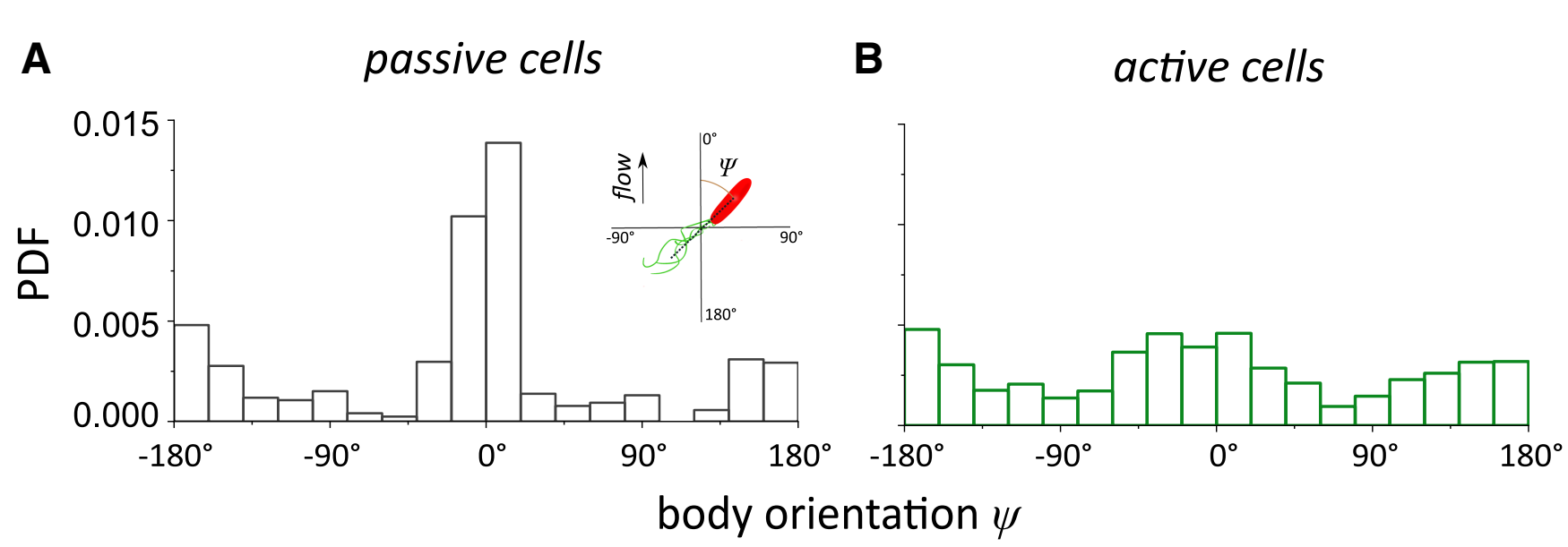

Figure 3. Flow alignment of cell bodies of motile and nonmotile bacteria. Distribution of body orientations (ψ angles) of (A) nonmotile mutant cells and (B) motile wild-type cells with respect to flow. Only unwrapped configurations (push and pull for swimming cells) were considered; shear rate $\gamma = 3.0\ s^{-1}$, measurement h = 20 μm above the bottom surface. Numbers of passive cells $n_1 = 19$ (A) and active cells $n_2 = 116$ (B). The number of data points are 3,319 frames (A) and 2,397 frames (B). For statistical analysis and comparison of the histograms, see supplemental information.

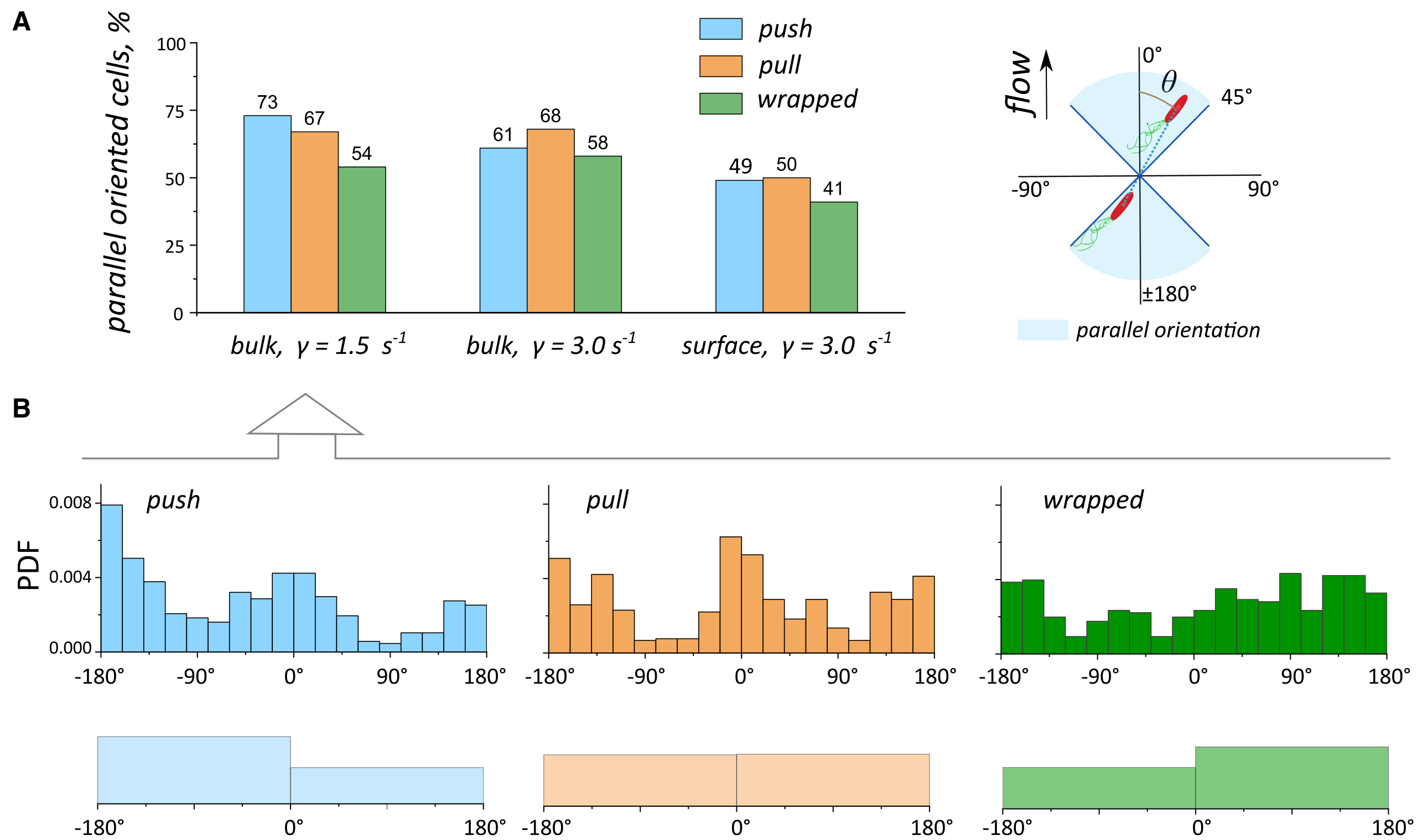

Figure 5. Flow alignment of bacteria in dependence on the swimming mode. (A) Percentage of displacements parallel to the direction of fluid flow, i.e., within a deviation from streamlines of less than $\pm 45°$; see insert on the right; "bulk" refers to $h = 20$ μm, and "surface" refers to $h = 5$ μm above bottom surface of the channel. (B) Distributions of θ angles is separated according to swimming modes ($h = 20$ μm, $\gamma = 1.5$ s^{-1}). The time lag is $\Delta T = 0.05$ s. Numbers of pushers $n_1 = 75$ (left), pullers $n_2 = 70$ (middle), and wrapped mode swimmers $n_3 = 43$ (right). The number of data points are 437 (left), 521 (middle), and 427 (right). For statistical analysis and comparison of the histograms, see supplemental information.

The bar plots confirm that in a shear flow far from solid surfaces, swimming directions preferentially align with the fluid flow, whereas alignment vanishes at the surface. With respect to the different swimming modes, these results moreover indicate that push and pull modes have an increased tendency to align as compared with cells swimming in wrapped mode. Comparing different shear rates far from the wall (left and middle bar plot), we observed that differences between the swimming modes became less pronounced at higher shear rate. As frequent turn events in the trajectories of active swimmers limit the degree of alignment that can be achieved, we speculate that with increasing shear rate, the different swimming modes eventually saturate at similar levels of alignment.

We also considered the temporal alignment dynamics of individual swimmers (see Figure S4 in supplemental information). For long episodes in push or pull mode, the θ angle mostly fluctuates around 180° (against flow direction). Cells in wrapped mode, in contrast, reorient faster and undergo more pronounced changes in their swimming direction. Note, however, that only small numbers of sufficiently long trajectories were available in our data, so that we cannot provide a rigorous statistical analysis of the alignment dynamics.

In Figure 5B, we consider the full histograms of displacement angles θ of all three swimming modes for a shear flow of $\gamma = 1.5$ s^{-1} and $h = 5$ μm away from a solid surface. In agreement with the bar plots in Figure 5A, the histograms of push and pull modes exhibit pronounced peaks at 0° and 180°, indicating alignment in flow direction, while no clear pattern is observed in the histogram of the wrapped mode. Interestingly, the histogram for *P. putida* swimmers in push mode additionally shows a preferred directional drift across streamlines in $-x$ direction (see also Figure 1A), which means toward the right-hand side with respect to the negative flow ($-y$) direction. This can be seen as an indication of bacterial rheotaxis in bulk resulting from an interplay between the helical flagellar bundle and the velocity gradient of the fluid shear flow.[29,31] Even though a similar chiral flagellar tuft is present in both pushers and pullers, contrary to our expectations, no drift was observed for runs in pulling mode. However, the compact wrapped configuration of the swimmer, which does not exhibit pronounced alignment with the streamlines, shows a directional bias to the opposite side, which, together with the behavior of pushers and pullers, will be considered in the following numerical simulations.

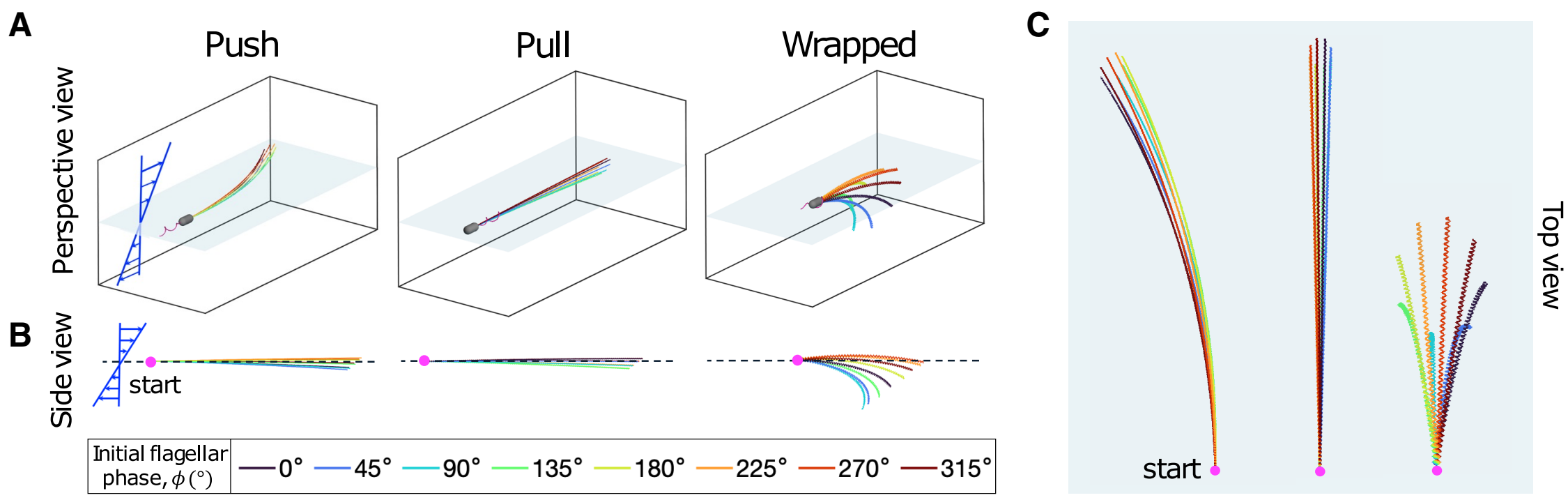

Figure 6. Numerical simulations: trajectories of bacteria in a shear flow. Trajectories of swimmers in push, pull, and wrapped modes under shear flow conditions. (A) Perspective, (B) side, and (C) top views are shown for each swimming mode. In each view, eight trajectories, corresponding to eight different initial phases ϕ, are displayed for each swimming mode. In the light blue plane, the flow velocity is zero. Each simulation is run for 0.8 s with a shear rate of $\gamma = 3.0\ s^{-1}$. The torque values for each mode are as follows: $\tau = 2.0$ nN·nm for push, $\tau = -2.0$ nN·nm for pull, and $\tau = -4.0$ nN·nm for the wrapped mode. Above the plane of focus, the fluid moves in line with the initial swimming direction (swimming with the flow), while below it moves against the initial swimming direction (swimming against the flow). See also the corresponding Video S3 and additional numerical data in Figure S9 in the supplemental information.

Numerical simulations confirm different rheotactic responsiveness of pushers and pullers

To further substantiate whether *P. putida* cells swimming in push and pull modes show different sensitivity in their rheotactic response to shear flow, we performed numerical simulations. Our computational cell model is based on the real geometry of *P. putida*, with the exception that the cell has a single superflagellum at one pole. This simplification assumes that all flagella, when driven by a constant torque, are synchronized to form a flagellar bundle. For each swimming mode, the flagellar motor applies a specific constant torque to produce that mode. No tumbles or transitions between swimming modes occur within a given simulation. To mimic the experimental setting, each cell is positioned in a shear flow, such that the fluid velocity is zero in the focal plane where the cell is located. By imposing zero velocity in the focal plane, the flow field in our simulations corresponds to the co-moving frame of reference of the cell; i.e., the flow speed in the focal plane, as seen from the laboratory frame of reference, was subtracted. A uniform shear rate of $\gamma = 3.0\ s^{-1}$ was applied in all simulations, unless stated otherwise (see materials and methods and supplemental information for further details).

Figure 6 shows trajectories of swimmers in different swimming modes and under shear flow conditions that we obtained from our numerical simulations. The plane of focus, where the flow velocity is zero, is indicated in light blue. The initial swimming direction was oriented within the plane of focus, aligned to the fluid flow in the adjacent planes. Above the plane of focus, the fluid moves in line with the initial swimming direction (swimming with the flow), while below it moves against the initial swimming direction (swimming against the flow). For each swimming mode, eight initial flagellar phases (ϕ) were considered, and the resulting trajectories are shown in three views: perspective, side, and top.

Similar to the experimental results in Figure 5B, also in the simulations, a drift motion across the streamlines was observed for a pusher configuration, while the puller stably follows the streamlines over time intervals even longer than the average duration of run episodes observed in experiments (see Table S3 in supplemental information). Interestingly, according to our simulations, swimmers in wrapped mode do not exhibit a directional bias but spread more rapidly in various directions as their trajectories are more strongly influenced by the initial flagellar phase than those of other swimming modes. Based on a larger set of numerical simulations that, in addition, also varies the initial body orientation of the swimmers in the shear flow, we recover histograms that capture most features observed in our experiments (see supplemental information).

Pullers are stabilized under shear flow conditions

For *P. putida* cells swimming in a fluid at rest, the different swimming modes and the transitions between them have been studied in detail.[15] When flagellar motors turn CCW, cells propagate in pushing mode with the flagellar bundle propelling the cell body from behind. Upon a switch to CW rotation, cells reverse their direction of motion and continue as pullers with the flagellar bundle pointing in the direction of motion. In most cases, pull runs are short-lived and transition to the wrapped mode of locomotion, where cells propagate in a screw thread fashion with their helical bundle wrapped around the cell body.[15,20] Numerical simulations suggest that an increase in the torque of the flagellar motors triggers the transition from pull to wrapped mode, where swimming speed and persistence of motion are decreased but motors continue to rotate in CW direction.[45] Once motors switch back to CCW rotation, swimming in wrapped mode ends, and the cell changes back to pushing mode.

Table 1. Statistics of occurrence of swimming modes

Shear rate γ, s^{-1}	Numbers of cells	Frequency of mode, % push	pull	wrapped	Ratio CW pull/wrap
0.0	116	45.7	21.5	32.8	0.66
1.5	188	39.9	37.2	22.9	1.62
3.0	462	43.7	30.1	26.2	1.15

Contributions of the three swimming modes to the total number of runs in fluid at rest and in two different shear flows. All data were recorded at a distance of $h = 20$ μm away from the surface.

Here, we experimentally studied whether shear flows affect the transitions and thus the frequencies at which the individual swimming modes are observed. Similar to earlier results,[19] a fraction of push runs (CCW rotation of the flagellar motors) between 40% and 50% was observed in fluid at rest, as well as in two different shear flows; see Table 1. Within the fraction of pull and wrapped runs (CW rotation of the flagellar motors), we confirmed a dominance of the wrapped mode in resting fluid; see Table 1 and Pfeifer et al.[19] However, under shear flow conditions, the wrapped mode is reduced to less than 50% within the population of CW runs and the pull mode dominates; see Table 1. The swimming speeds, by contrast, remained similar for cells in resting fluid and under shear condition, when measured at the same distance above the bottom surface of the channel; see Table S4 in supplemental information. These results suggest that filament wrapping becomes less likely in a fluid shear flow, thus stabilizing swimmers in pulling mode.

Numerical simulations: Critical torque for filament wrapping is increased in fluid shear flow

To confirm that filament wrapping is indeed affected under shear flow conditions, we performed numerical simulations of cells transitioning from the pull to the wrapped mode under shear flow, with the flagellar motor rotating CW, corresponding to negative values of the motor torque (τ). Each cell in pull mode was initially positioned in the focal plane, where the fluid velocity was zero; i.e., numerical simulations were performed in the co-moving frame of reference, as described above. Various combinations of motor torque (τ), shear rate (γ), and initial flagellar phase (ϕ) were considered.

Figure 7A displays four trajectories corresponding to four distinct initial flagellar phases ($\phi = 0°, 90°, 180°$, and $270°$; see inset), while all other parameters were held constant. It can be seen that the flagellar phase critically influences the turning angle during a transition from pull to wrapped mode. As a result, the initial flagellar phase affects subsequent bacterial spreading in the wrapped mode. Depending on the interplay of turning angle and shear flow geometry, different complex trajectory patterns may emerge. Figure 7B illustrates, as an example, the long-time periodic trajectory of a wrapped swimmer observed at high shear rates. The left and right panels correspond to the side and top views, respectively, as in Figure 6. After the flagellum wrapped around the cell body, the wrapped-mode swimmer oscillated across the focal plane between the regions of opposing flow directions. The detailed temporal evolution of a cell's transition into the wrapped mode can be seen in Figure 7C for $\tau = -4.0$ nN·nm, $\gamma = 50\ s^{-1}$, and $\phi = 90°$. At this torque magnitude, the flagellum buckles, causing the cell body to turn out of the focal plane, and hydrodynamic interactions lead to filament wrapping.

Finally, in Figure 7D, we show the resulting swimming mode as a function of motor torque and shear rate, starting from a pull mode swimmer with the motor rotating CW. As has been shown in previous simulations in resting fluid, a critical motor torque is required to initiate filament wrapping.[46] This is also confirmed here, where the pull mode persists for small motor torques, independent of the shear rate. Our simulations under flow conditions show that the critical torque that is required to initiate filament wrapping increases with increasing shear rate. Note that, for each choice of torque and shear rate, eight initial flagellar phases uniformly distributed over 360° were considered. The resulting swimming mode is generally independent of the initial phase, except near the threshold values, where different initial phases may lead to either pull or wrapped modes.

DISCUSSION

The environmental conditions of bacterial habitats, such as complex geometries or fluid flows, may strongly affect bacterial locomotion and navigation. In this work, we examined the impact of fluid shear flow on the motility pattern of the gram-negative soil bacterium *P. putida*, a lophotrichously flagellated, rod-shaped swimmer that exhibits runs in push, pull, and wrapped mode and serves as a model for multi-mode bacterial swimming.

We showed that active swimming perturbed shear-flow-induced alignment of the bacterial body axis with the flow direction. This can be attributed to the turning maneuvers that occur between run phases and repeatedly randomize the direction of motion and, hence, the body orientation of the motile wild-type cells. Nevertheless, under shear flow conditions in the bulk fluid (20 μm above the bottom surface of the channel), swimming trajectories exhibit a clear tendency to align with the flow direction. In contrast, close to a solid surface (5 μm above the bottom surface of the channel), flow alignment of the trajectories was no longer detectable. We assume that this is due to the wall-induced reorientation of swimmers parallel to the solid surface, so that the rod-shaped cell bodies are less affected by the shear gradient perpendicular to the surface.[44]

By considering runs in push, pull, and wrapped modes separately, we could show that alignment is more pronounced for pushers and pullers, while swimmers in

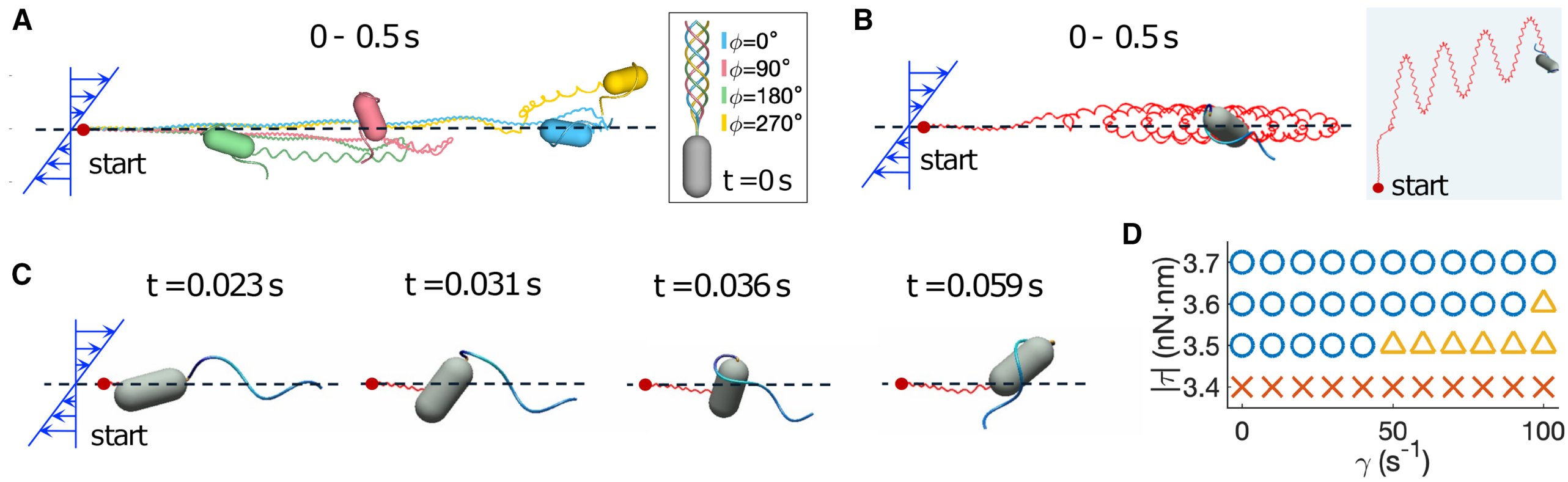

Figure 7. Numerical simulations: wrapped mode formation under shear flow. (A) Tracks of swimmers undergoing a pull-to-wrap transition for four different initial flagellar phases ϕ (see inset). The dashed line indicates the focal plane, where the flow speed is zero. The applied torque and the shear rate are $\tau = -3.5$ nN·nm and $\gamma = 20$ s^{-1}, respectively. (B) Typical periodic trajectory of a wrapped swimmer at high shear rates ($\tau = -3.7$ nN·nm, $\gamma = 50$ s^{-1}, and $\phi = 0°$). The left and right panels correspond to side and top views, respectively. (C) Snapshots showing time evolution of a cell's transition into the wrapped mode, $\tau = -4.0$ nN·nm, $\gamma = 50$ s^{-1}, and $\phi = 90°$. (D) Motor torque thresholds ($|\tau|$) for the formation of the wrapped mode at different shear rates (γ). Circles represent the wrapped mode, crosses represent the pull mode, and triangles represent cases where either mode may occur depending on the initial flagellar phase. See also the corresponding Videos S4, S5, and S6 in the supplemental information.

wrapped mode showed no clear tendency, neither far nor near the interface. This is presumably related to the compact shape of the wrapped mode as compared with the more elongated configurations of pushing and pulling swimmers (according to our fluorescence images, the aspect ratio of unwrapped swimmers is $\alpha \simeq 7.5$, while for wrapped swimmers it is only $\alpha \simeq 2.5$). At the same time, a rheotactic drift across streamlines was observed for swimmers in push mode in agreement with earlier literature.[29,30,32,47] Unexpectedly, however, only for the wrapped mode swimmer, an analogous effect was observed, while the trajectories of pullers were not affected over the time window of our observation. Numerical simulations relying on realistic swimmer geometries confirmed the experimental observations for pushers and pullers but did not show any directional preference for the wrapped mode swimmers. The absence of a rheotactic drift of pullers in both our experimental and numerical data does not agree with our current understanding of rheotaxis as a consequence of chirality-induced cell reorientation in a shear flow and remains an open question that requires further mechanistic studies.

As swimmers are floating along with the fluid flow, transitions between swimming modes and the associated directional changes may enhance the spreading efficiency. For example, a run-and-reverse swimming strategy was shown to be advantageous for efficiently tracking small food sources in dynamic fluid environments.[48] Here, we demonstrated that the transitions between swimming modes are affected by fluid shear flow conditions. Specifically, we observed that among the runs with CW-rotating motors, the ratio between straight runs in pull mode and less persistent runs in wrapped mode is shifted in favor of pullers, indicating that the pull mode becomes more stable in a shear flow. This is in line with earlier observations in *E. coli* showing that run times and flagellar transitions are affected by the presence of a fluid flow.[49] Our numerical modeling results suggest that the pull mode is stabilized due to mechanical interactions between the flagella and the fluid shear flow, where different fluid velocities in the vicinity of the cell body make filament wrapping more difficult. Thus, an increased critical torque is required for wrapping in a shear flow, as demonstrated by our systematic numerical simulations. As a result, more pullers and fewer wrapped swimmers are observed in a shear flow as compared with liquid at rest.

Note that in the experiments, wrapped mode formation is already reduced at shear rates of $\gamma = 1.5$ s^{-1} and 3.0 s^{-1}, while in numerical simulations an impact of the shear flow on wrapped mode formation is only observed for shear rates beyond $\gamma = 50.0$ s^{-1}. Besides uncertainties in some of the mechanical parameters of our numerical model, we assume that the main reason for this discrepancy is the number of flagella that was included in our numerical study. While *P. putida* carries a tuft of 5–7 flagella attached to one pole of the cell body, we approximate this flagellar bundle by a single helical filament in our model simulations. This may serve as a good approximation for a swimmer that moves in push mode. A pulling helical bundle, however, is less stable and prone to jamming at the filament intersection points.[50,51] This may facilitate transitions to the wrapped mode, so that lower shear forces are sufficient to hinder the pull-to-wrapped transition in the case of a filament bundle, while higher shear is required to suppress the wrapping of a single filament. Note also that this purely mechanical scenario does not involve any active regulation via receptor-mediated signaling.

Future studies of bacterial swimmers will focus on combining environments of complex geometry with hydrodynamic flows to mimic real-world habitats even more closely. They may also serve as a source of insight for the navigation of artificial microswimmers and may be

combined with advanced techniques to induce time-dependent local flow fields by bio-compatible light-sensitive materials.[52,53] Such experiments may also include mutant cell lines that allow for the adjustment of run lengths, stop events, and transitions from one swimming mode to another. Here, constructing so-called nontumbling mutants that propagate as push-only, pull-only, or wrapped-only swimmers will be particularly instructive to study the role of the different swimming modes within the complex environment.

CONCLUSION

In this study, we investigated how fluid shear flow influences the swimming behavior of *Pseudomonas putida*, a soil bacterium known for its multi-mode swimming strategy. Our experiments revealed that shear-induced alignment and rheotactic drift responses vary depending on the swimming mode. We furthermore observed that flagellar wrapping becomes less effective under higher shear stress. Numerical simulations based on realistic swimmer geometries support the experimental findings. While still at an early stage, we hope to contribute with our findings to predicting the spreading of bacterial infections in complex surroundings as well as to the engineering of artificial microswimmers designed to navigate specific habitats.

DATA AVAILABILITY

The original code has been deposited at Zenodo under https://doi.org/10.5281/zenodo.17456571 and is publicly available as of the date of publication. Any additional information required to reanalyze the data reported in this paper is available from the lead contact upon request.

SUPPLEMENTAL CITATION

Reference 54 is cited in the supplemental information.

ACKNOWLEDGMENTS

V.F. acknowledges the kind hospitality of the Erwin Schrödinger Institute (ESI) in Vienna during the workshop “Nonequilibrium processes in Physics and Biology,” where part of this project was productively discussed. The research has been partially funded by the International Max Planck Research School on Multiscale Bio-Systems (V.F.) and by the Deutsche Forschungsgemeinschaft, Germany, project ID 443369470 – BE 3978/13-1 (V.F., V.P., and C.B.) as well as project ID 318763901 – SFB1294 (A.D., V.P., R.G., and C.B.). S.L. was supported by the NSF (CBET-2415406) and the Charles Phelps Taft Research Center at the University of Cincinnati, USA. Y.K. was supported by the National Research Foundation of Korea, South Korea (RS-2023-00247232). W.L. was supported in part by the National Institute for Mathematical Sciences, South Korea (B25910000) and in part by the National Research Foundation of Korea,-South Korea (MSIT) (RS-2025-16071334). During most of the period in which this work was carried out, J.P. was a member of the Institute for Advanced Study, supported by the AMIAS Fund, with access to IAS computing resources.

AUTHOR CONTRIBUTIONS

V.F. designed and conducted experiments, analyzed experimental data, and drafted the manuscript. A.D. designed the image analysis toolbox, analyzed experimental data, and wrote the manuscript. S.L. and J.P. designed the numerical settings and wrote the manuscript. J.P., Y.K., and W.L. performed numerical simulations. J.P. processed and visualized numerical data. R.G. performed statistical analysis and Bayesian inference of bacterial alignment and wrote the corresponding section in the supplemental information. V.P. established the fluorescent staining procedure and generated the stator mutant. C.B. designed the research and wrote the manuscript. C.B. and S.L. supervised the whole project. All authors reviewed and edited the manuscript, discussed the results, and contributed to the final manuscript.

DECLARATION OF INTERESTS

The authors declare no competing interests.

SUPPLEMENTAL INFORMATION

Supplemental information can be found online at https://doi.org/10.1016/j.bpj.2026.05.032.

REFERENCES

1. Lauga, E. 2020. The Fluid Dynamics of Cell Motility. Cambridge Texts in Applied Mathematics. Cambridge University Press.
2. Elgeti, J., R. G. Winkler, and G. Gompper. 2015. Physics of Microswimmers—Single Particle Motion and Collective Behavior: A Review. *Rep. Prog. Phys.* 78:056601.
3. Gaffney, E. A., H. Gadêlha, …, J. C. Kirkman-Brown. 2011. Mammalian Sperm Motility: Observation and Theory. *Annu. Rev. Fluid Mech.* 43:501–528.
4. Alvarez, L., B. M. Friedrich, …, U. B. Kaupp. 2014. The Computational Sperm Cell. *Trends Cell Biol.* 24:198–207.
5. Berg, H. C. 2004. *E. coli* in Motion. Biological and Medical Physics, Biomedical Engineering. Springer.
6. Turner, L., W. S. Ryu, and H. C. Berg. 2000. Real-Time Imaging of Fluorescent Flagellar Filaments. *J. Bacteriol.* 182:2793–2801.
7. Grognot, M., and K. M. Taute. 2021. More than Propellers: How Flagella Shape Bacterial Motility Behaviors. *Curr. Opin. Microbiol.* 61:73–81.
8. Xie, L., T. Altindal, …, X.-L. Wu. 2011. Bacterial Flagellum as a Propeller and as a Rudder for Efficient Chemotaxis. *Proc. Natl. Acad. Sci.* 108:2246–2251.
9. Theves, M., J. Taktikos, …, C. Beta. 2013. A Bacterial Swimmer with Two Alternating Speeds of Propagation. *Biophys. J.* 105:1915–1924.
10. Theves, M., J. Taktikos, …, C. Beta. 2015. Random Walk Patterns of a Soil Bacterium in Open and Confined Environments. *EPL.* 109:28007.
11. Raatz, M., M. Hintsche, …, C. Beta. 2015. Swimming Patterns of a Polarly Flagellated Bacterium in Environments of Increasing Complexity. *Eur. Phys. J. Spec. Top.* 224:1185–1198.
12. Weber, A., M. Bahrs, …, V. Zaburdaev. 2019. Rectification of Bacterial Diffusion in Microfluidic Labyrinths. *Front. Phys.* 7:148.
13. Datta, A., S. Beier, …, C. Beta. 2025. Bacterial Swimming in Porous Gels Exhibits Intermittent Run Motility with Active Turns and Mechanical Trapping. *Sci. Rep.* 15:20320.
14. Harwood, C. S., K. Fosnaugh, and M. Dispensa. 1989. Flagellation of *Pseudomonas putida* and Analysis of Its Motile Behavior. *J. Bacteriol.* 171:4063–4066.
15. Hintsche, M., V. Waljor, …, C. Beta. 2017. A Polar Bundle of Flagella Can Drive Bacterial Swimming by Pushing, Pulling, or Coiling around the Cell Body. *Sci. Rep.* 7:16771.

16. Kühn, M. J., F. K. Schmidt, …, K. M. Thormann. 2017. Bacteria Exploit a Polymorphic Instability of the Flagellar Filament to Escape from Traps. *Proc. Natl. Acad. Sci.* 114:6340–6345.

17. Kinosita, Y., Y. Kikuchi, …, T. Nishizaka. 2017. Unforeseen Swimming and Gliding Mode of an Insect Gut Symbiont, *Burkholderia* Sp. RPE64, with Wrapping of the Flagella around Its Cell Body. *ISME J.* 12:838–848.

18. Tian, M., Z. Wu, …, J. Yuan. 2022. A New Mode of Swimming in Singly Flagellated *Pseudomonas aeruginosa*. *Proc. Natl. Acad. Sci.* 119:e2120508119.

19. Pfeifer, V., S. Beier, …, C. Beta. 2022. Role of the Two Flagellar Stators in Swimming Motility of *Pseudomonas putida*. *mBio.* 13:e0218222.

20. Alirezaeizanjani, Z., R. Großmann, …, C. Beta. 2020. Chemotaxis Strategies of Bacteria with Multiple Run Modes. *Sci. Adv.* 6:eaaz6153.

21. Thormann, K. M., C. Beta, and M. J. Kühn. 2022. Wrapped up: The Motility of Polarly Flagellated Bacteria. *Annu. Rev. Microbiol.* 76:349–367.

22. Rusconi, R., J. S. Guasto, and R. Stocker. 2014. Bacterial Transport Suppressed by Fluid Shear. *Nat. Phys.* 10:212–217.

23. Wheeler, J. D., E. Secchi, …, R. Stocker. 2019. Not Just Going with the Flow: The Effects of Fluid Flow on Bacteria and Plankton. *Annu. Rev. Cell Dev. Biol.* 35:213–237.

24. Jeffery, G. B. 1922. The Motion of Ellipsoidal Particles Immersed in a Viscous Fluid. *Proc. R. Soc. Lond. Ser. A.* 102:161–179.

25. Bretherton, F. P. 1962. The Motion of Rigid Particles in a Shear Flow at Low Reynolds Number. *J. Fluid Mech.* 14:284–304.

26. Ishimoto, K. 2023. Jeffery's Orbits and Microswimmers in Flows: A Theoretical Review. *J. Phys. Soc. Jpn.* 92:062001.

27. Uppaluri, S., N. Heddergott, …, T. Pfohl. 2012. Flow Loading Induces Oscillatory Trajectories in a Bloodstream Parasite. *Biophys. J.* 103:1162–1169.

28. Zöttl, A., and H. Stark. 2013. Periodic and Quasiperiodic Motion of an Elongated Microswimmer in Poiseuille Flow. *Eur. Phys. J. E.* 36:4.

29. Marcos, H. C. Fu, T. R. Powers, and R. Stocker. 2012. Bacterial Rheotaxis. *Proc. Natl. Acad. Sci.* 109:4780–4785.

30. Zhang, Z., J. Liu, …, Y. Sun. 2016. Human Sperm Rheotaxis: A Passive Physical Process. *Sci. Rep.* 6:23553.

31. Mathijssen, A. J. T. M., N. Figueroa-Morales, …, A. Zöttl. 2019. Oscillatory Surface Rheotaxis of Swimming *E. coli* Bacteria. *Nat. Commun.* 10:3434.

32. Jing, G., A. Zöttl, …, A. Lindner. 2020. Chirality-Induced Bacterial Rheotaxis in Bulk Shear Flows. *Sci. Adv.* 6:eabb2012.

33. Zöttl, A., F. Tesser, …, A. Lindner. 2023. Asymmetric Bistability of Chiral Particle Orientation in Viscous Shear Flows. *Proc. Natl. Acad. Sci.* 120:e2310939120.

34. Li, R., G. Gompper, and M. Ripoll. 2021. Tumbling and Vorticity Drift of Flexible Helicoidal Polymers in Shear Flow. *Macromolecules.* 54:812–823.

35. Makino, M., and M. Doi. 2005. Migration of Twisted Ribbon-like Particles in Simple Shear Flow. *Phys. Fluids.* 17:103605.

36. Kaya, T., and H. Koser. 2012. Direct Upstream Motility in *Escherichia coli*. *Biophys. J.* 102:1514–1523.

37. Kim, D., Y. Kim, and S. Lim. 2022. Effects of Swimming Environment on Bacterial Motility. *Phys. Fluids.* 34:031907.

38. Lee, W., Y. Kim, …, S. Lim. 2021. A Novel Computational Approach to Simulate Microswimmers Propelled by Bacterial Flagella. *Phys. Fluids.* 33:111903.

39. Lee, W., Y. Kim, and S. Lim. 2023. Bio-Inspired in Silico Microswimmer: Run and Tumble Kinematics. *Phys. Fluids.* 35:031908.

40. Pfeifer, V., V. Muraveva, and C. Beta. 2024. Flagella and Cell Body Staining of Bacteria with Fluorescent Dyes. *In* Cell Motility and Chemotaxis: Methods and Protocols. C. Beta and C. Martinez-Torres, eds Springer, pp. 79–85.

41. Datta, A. 2025. TrackScoPy: A Python Toolbox for Tracking and Analyzing Bacterial Motility from Fluorescence Microscopy Videos, Zenodo. https://doi.org/10.5281/zenodo.17456571.

42. Olson, S. D., S. Lim, and R. Cortez. 2013. Modeling the Dynamics of an Elastic Rod with Intrinsic Curvature and Twist Using a Regularized Stokes Formulation. *J. Comput. Phys.* 238:169–187.

43. Zöttl, A., K. E. Klop, …, D. G. A. L. Aarts. 2019. Dynamics of Individual Brownian Rods in a Microchannel Flow. *Soft Matter.* 15:5810–5814.

44. Rubio, L. D., M. Potomkin, …, I. S. Aranson. 2021. Self-propulsion and Shear Flow Align Active Particles in Nozzles and Channels. *Adv. Intell. Syst.* 3:2000178.

45. Park, J., Y. Kim, …, S. Lim. 2024. Bundling Instability of Lophotrichous Bacteria. *Phys. Fluids.* 36:101917.

46. Park, J., Y. Kim, …, S. Lim. 2022. Modeling of Lophotrichous Bacteria Reveals Key Factors for Swimming Reorientation. *Sci. Rep.* 12:6482.

47. Stocker, R. 2011. Reverse and Flick: Hybrid Locomotion in Bacteria. *Proc. Natl. Acad. Sci.* 108:2635–2636.

48. Guseva, K., and U. Feudel. 2025. Advantages of Run-Reverse Motility Pattern of Bacteria for Tracking Light and Small Food Sources in Dynamic Fluid Environments. *J. R. Soc. Interface.* 22:20250037.

49. Liu, G., Z. Liu, …, J. Yuan. 2021. Upcoming Flow Promotes the Bundle Formation of Bacterial Flagella. *Biophys. J.* 120:4391–4398.

50. Macnab, R. M. 1977. Bacterial Flagella Rotating in Bundles: A Study in Helical Geometry. *Proc. Natl. Acad. Sci.* 74:221–225.

51. Kim, M., J. C. Bird, …, T. R. Powers. 2003. A Macroscopic Scale Model of Bacterial Flagellar Bundling. *Proc. Natl. Acad. Sci.* 100:15481–15485.

52. Muraveva, V., N. Lomadze, …, S. Santer. 2024. Manipulation of Artificial and Living Small Objects by Light Driven Diffusioosmotic Flow. *Sci. Rep.* 14:18342.

53. Umlandt, M., P. Arya, …, S. Santer. 2025. Shaping Microbial Motion with Light: A Contactless Approach Using Diffusioosmotic Flow. *Adv. Mater. Interfaces.* 12:e00663.

54. Crocker, J. C., and D. G. Grier. 1996. Methods of Digital Video Microscopy for Colloidal Studies. *J. Colloid Interface Sci.* 179:298–310.

Biophysical Journal, Volume *125*

Supplemental information

Swimming patterns of a multi-mode bacterial swimmer in fluid shear flow

Valeriia Fast, Agniva Datta, Jeungeun Park, Robert Großmann, Veronika Pfeifer, Yongsam Kim, Wanho Lee, Sookkyung Lim, and Carsten Beta

SUPPLEMENTAL MATERIALS

1 Semi-automatic analysis of trajectories

In this section, we discuss how the trajectories are obtained from microscope images and the criteria we use to identify the different swim modes. To track and distinguish cells in different swimming modes, bacteria are stained with fluorescent dyes (red for the body and green for the flagella), see Main Text and [1]. Preliminary processing (signal splitting, frame cropping) of the fluorescent recordings is done with freely available *ImageJ* software as described in the Main Text [2]. For further tracking and analysis, the lab-made Python code [3] is used, which is provided along with this manuscript. The workflow is also shown in Figure 2 in the Main Text.

1.1 Cell tracking

The raw images from the microscope are a stack of *uint16* images consisting of two colored channels, namely the red and the green ones, which capture the staining of the cell body and the flagella bundle of the bacterial cells, respectively. As a first step, we split the image stacks into the red and green channels, thereby obtaining a pair of grey-scale image stacks. Then we perform segmentation and tracking separately on the two image stacks to extract the trajectories corresponding to the center of masses of the segmented cell body and flagella of each moving cell. For the last step, we use the method described in publication [4], which is based on Crocker's processing algorithm. Detailed description of the last step can be found in [5].

1.2 Distinction of swim-modes

We match the coordinates of the cell trajectories (obtained from the red channel) and the flagella trajectories (obtained from the green channel). Based on a minimum displacement δx and minimum time difference δt between the coordinates of each point in the cell and flagella trajectories, we identify the pairs of trajectories which correspond to the individual bacteria. Then, for each identified pair and each frame of the image stacks, we define a vector from the coordinate of the center of mass of a cell to that of the corresponding identified flagella bundle, which we call the pointing vector $\boldsymbol{P}_{i,j}$ and also the velocity vector $\boldsymbol{v}_{i,j}$ corresponding to the displacement of the cell from the previous frame (see Figure S1)., defined as follows:

$$\boldsymbol{P}_{i,j} = \left(x^f_{i,j} - x^c_{i,j}\right)\hat{\imath} + \left(y^f_{i,j} - y^c_{i,j}\right)\hat{\jmath}, \quad (1a)$$

$$\boldsymbol{v}_{i,j} = \left(x^c_{i,j} - x^c_{i-1,j}\right)\hat{\imath} + \left(y^c_{i,j} - y^c_{i-1,j}\right)\hat{\jmath}. \quad (1b)$$

Here, the index i corresponds to the ith frame of the image, index j corresponds to the jth pair of tracks (one bacterium), index c corresponds to the coordinates of the center of mass of cell (obtained from red channel) and the index f corresponds to the coordinates of the center of mass of flagella bundle (obtained from green channel). We determine the swim-mode corresponding to the jth bacterium in the ith frame by the inner product of $\boldsymbol{P}_{i,j}$ and $\boldsymbol{v}_{i,j}$ as shown in Table S1.

It is important to note here that the distinction is based on the parameters δx, δt and ϵ, which show little variability from image to image, especially for separate datasets due to the heterogeneity of the bacterial population and quality of recorded images. Further, to calculate the flow drift velocity, we analyze some non-swimming cells (pre-selected manually) in the cell suspension. Then, we perform segmentation and tracking on the red channel of the recorded images. Thereby, we obtain the drift velocity $\boldsymbol{v}^{\text{drift}}_{i,j}$ corresponding to the

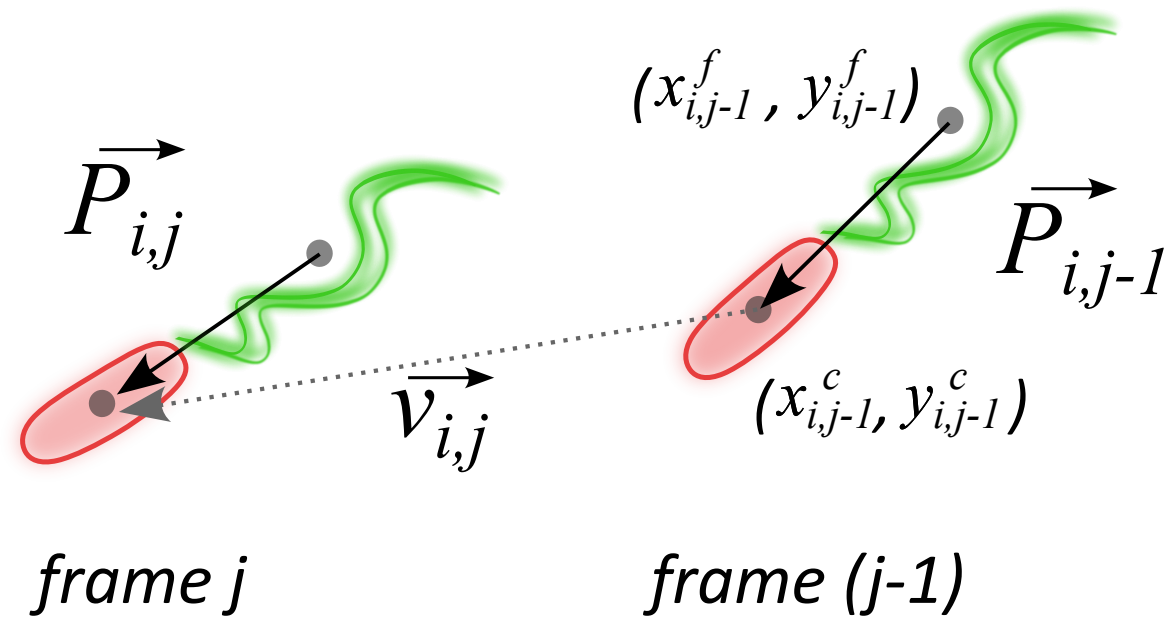

Figure S1: Schematic illustration of vectors of a single swimmer for the swim-mode detection.

swim-mode	criteria
push	$\boldsymbol{P}_{i,j}.\boldsymbol{v}_{i,j} > 0$
pull	$\boldsymbol{P}_{i,j}.\boldsymbol{v}_{i,j} < 0$
wrapped	$\lvert\boldsymbol{P}_{i,j}\rvert < \epsilon$

Table S1: Criteria to identify swim-modes from a pair of trajectories in a particular frame. The threshold for cell body-flagella is distance $\epsilon = 1.1\,\mu\text{m}$ for *P. putida* cells.

flow by taking the average of the velocities calculated from the derived trajectories. Then, we modify the detection criteria by taking the inner product of $\boldsymbol{P}_{i,j}$ and the relative velocity $\boldsymbol{v}^{\text{rel}}_{i,j}$, obtained by subtracting the flow velocity from the observed velocity.

$$\boldsymbol{v}^{\text{rel}}_{i,j} = \boldsymbol{v}_{i,j} - \boldsymbol{v}^{\text{drift}}_{i,j} \tag{2}$$

After, the same criteria are used to distinguish the swim-modes by replacing $\boldsymbol{v}_{i,j}$ by $\boldsymbol{v}^{\text{rel}}_{i,j}$ in Table S1.

Also, $\boldsymbol{v}^{\text{drift}}_{i,j}$ vector is used to define the orientations of the cells with respect to the flow streamlines. We define the angle between the pointing vector $\boldsymbol{P}_{i,j}$ and fluid flow direction, calculated above, as ψ angle (body's orientation), and the angle between the cell's velocity vector $\boldsymbol{v}_{i,j}$ and fluid flow direction as θ angle (the direction of the displacement), see also Figure 1D in the Main Text. The ψ angles (body's orientation) are defined only for unwrapped cells' configurations (push, pull and mutant non-motile cells).

2 Mathematical framework

P. putida consists of a rod-shaped cell body with a tuft of multiple flagella anchored at one pole, and it propels itself through fluid via flagellar rotation. Our computational model is constructed based on the experimentally observed geometry of *P. putida* and incorporates the relevant biological data. For simplicity, the cell model includes a single polar flagellum representing a bundled configuration of multiple flagella. The initial setting and parameter values used in the simulations are provided in Figure S2 and Table S2, respectively.

Flagellar dynamics is modeled using Kirchhoff rod theory, while the cell body is treated as a rigid body. The forces and torques generated by flagellar rotation are transmitted to the surrounding fluid which is governed by the incompressible Stokes equations. To solve these coupled equations, we employ the immersed boundary framework that is useful to solve fluid-structure interaction (FSI) problems. This approach captures the hydrodynamic interactions between the cell and the fluid, enabling bidirectional coupling between the cell motion and the resulting fluid flow. Importantly, the flagellar motor generates a constant torque, introduced as a control parameter, that drives flagellar rotation, while an equal and opposite torque is applied to the cell body, resulting in its counterrotation. The swimming motion is computed

by enforcing the condition that the net force and torque exerted on the surrounding fluid are both zero. Additionally, in the presence of shear flow, another parameter is introduced to control the imposed shear rate. We begin by describing the motion of a model cell composed of a rod-shaped cell body and a single left-handed superflagellum, formulated in Lagrangian coordinates. Subsequently, we present the governing fluid equations using the regularized Stokeslet formulation expressed in Eulerian coordinates. A detailed description of the method can be found in [6, 7] and therein; key features of the approach are summarized below.

The cell body is modeled as a spherocylinder and discretized into n_b points on its surface. It is represented in two forms, $\mathbf{X}_i^\mathrm{b}(t)$ and $\mathbf{Y}_i^\mathrm{b}(t)$, for $i = 1, 2, ..., n_\mathrm{b}$. The former corresponds to the physical body that interacts with the surrounding fluid, while the latter represents a rigid body that does not directly interact with the fluid. Each pair of corresponding points $\mathbf{X}_i^\mathrm{b}(t)$ and $\mathbf{Y}_i^\mathrm{b}(t)$ is connected via a stiff spring which generates a restoring force of the form

$$\mathbf{F}_i^b(t) = K(\mathbf{X}_i^\mathrm{b}(t) - \mathbf{Y}_i^\mathrm{b}(t)), \tag{3}$$

where K is a large stiffness constant that ensures the two representations remain close to each other. The force $\mathbf{F}_i^b(t)$ is applied to the rigid body at $\mathbf{Y}_i^\mathrm{b}(t)$, while an equal and opposite force $-\mathbf{F}_i^b(t)$ is exerted on the fluid at $\mathbf{X}_i^\mathrm{b}(t)$. The reference configuration of the rigid cell body is defined by a set of time-independent points $\mathbf{Z}_i$ that satisfy $\sum_{i=1}^{n_\mathrm{b}} \mathbf{Z}_i = \mathbf{0}$. The position of the rigid body at time t is then given by

$$\mathbf{Y}_i^\mathrm{b}(t) = \mathbf{C}(t) + \mathcal{R}(t)\mathbf{Z}_i, \tag{4}$$

where $\mathbf{C}(t)$ is the centroid of the rigid cell body and $\mathcal{R}(t)$ is a rotation matrix.

Let $\mathbf{f}^\mathrm{b}(t)$ and $\mathbf{n}^\mathrm{b}(t)$ denote the external force and torque, respectively, acting on the cell body, excluding contributions from the spring connections. Then, the balance of linear and angular momentum for the rigid body is given by

$$0 = \mathbf{f}^\mathrm{b}(t) + \sum_{i=1}^{n_\mathrm{b}} \mathbf{F}_i^\mathrm{b}(t), \qquad 0 = \mathbf{n}^\mathrm{b}(t) + \sum_{i=1}^{n_\mathrm{b}} (\mathcal{R}(t)\mathbf{Z}_i) \times \mathbf{F}_i^\mathrm{b}(t). \tag{5}$$

Given $\mathbf{f}^\mathrm{b}(t)$, $\mathbf{n}^\mathrm{b}(t)$ and $\mathbf{X}_i^\mathrm{b}(t)$ at each time t, we can compute $\mathbf{C}(t)$, $\mathcal{R}(t)$, and thus $\mathbf{Y}_i^\mathrm{b}(t)$ by solving the above system approximately [7]. The accuracy of the approximation improves with increasing spring stiffness K.

The motion of a flagellum is described by Kirchhoff rod theory. A flagellum is represented as a tapered helical curve $\mathbf{X}(s,t)$ together with orthonormal triads $\{\mathbf{D}^1(s,t), \mathbf{D}^2(s,t), \mathbf{D}^3(s,t)\}$ along the curve. This curve represents the centerline of the flagellum and the triads show the degree of bending and twisting. Let $\mathbf{F}(s,t)$ and $\mathbf{N}(s,t)$ be the force and the moment, respectively, which are transmitted across a section of the rod at the Lagrangian coordinate s at time t. Let $\mathbf{f}(s,t)$ and $\mathbf{n}(s,t)$ be the applied force and the torque densities, respectively. Then the linear and angular momentum balance equations are described as follows:

$$0 = \mathbf{f} + \frac{\partial \mathbf{F}}{\partial s}, \qquad 0 = \mathbf{n} + \frac{\partial \mathbf{N}}{\partial s} + \frac{\partial \mathbf{X}}{\partial s} \times \mathbf{F}. \tag{6}$$

Here, the internal force $\mathbf{F}$ and torque $\mathbf{N}$ can be written as follows:

$$\mathbf{F} = \sum_{i=1}^{3} b_i \Big(\mathbf{D}^i \cdot \frac{\partial \mathbf{X}}{\partial s} - \delta_{3i}\Big)\mathbf{D}^i, \qquad \mathbf{N} = \sum_{i=1}^{3} a_i \Big(\frac{\partial \mathbf{D}^j}{\partial s} \cdot \mathbf{D}^k - \kappa_i\Big)\mathbf{D}^i, \tag{7}$$

where δ_{3i} is the Kronecker delta, (i, j, k) is a cyclic permutation of $(1, 2, 3)$, and κ_i describes the intrinsic curvature (κ_1 and κ_2) and twist (κ_3). The constitutive relations can be derived from a variational derivative of the elastic energy functional for the unconstrained version of the Kirchhoff rod,

$$E = \frac{1}{2}\Big[\sum_{i=1}^{3} a_i \Big(\frac{\partial \mathbf{D}^j}{\partial s} \cdot \mathbf{D}^k - \kappa_i\Big)^2 + \sum_{i=1}^{3} b_i \Big(\mathbf{D}^i \cdot \frac{\partial \mathbf{X}}{\partial s} - \delta_{3i}\Big)^2\Big] ds, \tag{8}$$

where the parameters a_1 and a_2 are bending moduli, a_3 is the twist modulus, b_1 and b_2 are shearing moduli, and b_3 is the stretching modulus. To model the compliant hook, the bending modulus of the hook a^hook is reduced by a factor of 25 compared to that of the flagellum, reflecting its increased flexibility.

The tapered end of the flagellum is embedded into the cell body at the pole, with the flagellar axis aligned with the normal direction $\mathbf{E}(t)$ to the cell surface. The flagellar motor generates a constant torque τ in this normal direction at the motor point $\mathbf{X}(s_{\mathrm{mot}}, t)$, given by $\mathbf{N}(s_{\mathrm{mot}}, t) = -\tau\mathbf{E}(t)$. Positive values of τ correspond to counterclockwise rotation, while negative values correspond to clockwise rotation. This motor torque drives the rotation of the flagellum, enabling the cell to swim through the surrounding fluid.

Finally, the cell motion described above is coupled with the surrounding fluid which is governed by the incompressible Stokes equations:

$$0 = -\nabla p + \mu\Delta\mathbf{u} + \mathbf{g}, \qquad 0 = \nabla\cdot\mathbf{u}, \tag{9}$$

where the fluid velocity $\mathbf{u}$ and pressure p are unknown functions of the Cartesian coordinates $\mathbf{x}$ and time t, and μ is the dynamic viscosity of the fluid. The regularized force density $\mathbf{g}$ applied to the fluid by the immersed cell is given by:

$$\begin{aligned}\mathbf{g}(\mathbf{x}, t) = &\int_0^L \big[-\mathbf{f}(s,t)\big]\varphi_\varepsilon\big(\mathbf{x} - \mathbf{X}(s,t)\big)ds + \frac{1}{2}\nabla\times\int_0^L \big[-\mathbf{n}(s,t)\big]\varphi_\varepsilon\big(\mathbf{x} - \mathbf{X}(s,t)\big)ds \\ &+ \sum_{i=1}^{n_{\mathrm{b}}}\int_{L'}^L \big[-\mathbf{f}_i^{\mathrm{r}}(s,t)\big]\varphi_\varepsilon\big(\mathbf{x} - \mathbf{X}(s,t)\big)ds + \sum_{i=1}^{n_{\mathrm{b}}}\big[\int_{L'}^L \mathbf{f}_i^{\mathrm{r}}(s,t)ds\big]\varphi_\varepsilon\big(\mathbf{x} - \mathbf{X}_i^{\mathrm{b}}(t)\big) \\ &+ \sum_{i=1}^{n_{\mathrm{b}}}(-\mathbf{F}_i^{\mathrm{b}}(t))\varphi_\varepsilon(\mathbf{x} - \mathbf{X}_i^{\mathrm{b}}(t)).\end{aligned} \tag{10}$$

The first and second terms on the right-hand side represent the force and torque, respectively, exerted by the flagellum, and the third and fourth terms correspond to a short-range repulsive force that prevents contact between the flagellum and the cell body. Note that the repulsive force is not activated near the motor end of the filament by setting the lower limit L' of the integral to a value greater than zero. The fifth term accounts for the force exerted by the cell body, as discussed above. The bell-shaped blob function φ_ε, with infinite support, is defined as

$$\varphi_\varepsilon(\mathbf{r}) = \frac{15\varepsilon^4}{8\pi(\|\mathbf{r}\|^2 + \varepsilon^2)^{7/2}}, \tag{11}$$

where ε is the regularization parameter. The blob function satisfies $\int_{\mathbb{R}^3}\varphi_\varepsilon(\mathbf{r})d\mathbf{r} = 1$, enabling smooth transfer of forces and torques from Lagrangian points of the model cell to the Eulerian fluid domain. The motion of the cell, including the body points $\mathbf{X}_i^{\mathrm{b}}$ and the flagellar configuration $\{\mathbf{X}, \mathbf{D}^1, \mathbf{D}^2, \mathbf{D}^3\}$, is governed by the following equations:

$$\begin{aligned}\frac{\partial\mathbf{X}}{\partial t}(s,t) &= \mathbf{u}\big(\mathbf{X}(s,t),t\big) - \frac{1}{\alpha_1}\Big[\mathbf{f}(s,t) + \sum_{i=1}^{n_{\mathrm{b}}}\mathbf{f}_i^{\mathrm{r}}(s,t)\Big], \\ \frac{\partial\mathbf{X}_i^{\mathrm{b}}}{\partial t}(t) &= \mathbf{u}(\mathbf{X}_i^{\mathrm{b}}(t),t) - \frac{1}{\alpha_2}\Big[\mathbf{F}_i^{\mathrm{b}}(t) - \int_{L'}^L \mathbf{f}_i^{\mathrm{r}}(s,t)ds\Big], \qquad i = 1,...,n_{\mathrm{b}}, \\ \frac{\partial\mathbf{D}^j}{\partial t}(s,t) &= \Big[\mathbf{w}\big(\mathbf{X}(s,t),t\big) - \frac{1}{\beta}n_3(s,t)\mathbf{D}^3(s,t)\Big]\times\mathbf{D}^j(s,t), \qquad j = 1,2,3,\end{aligned} \tag{12}$$

where the angular velocity of the fluid is given as $\mathbf{w} = \dfrac{1}{2}\nabla\times\mathbf{u}(\mathbf{x},t)$. The parameters α_i's and β are translational and rotational drag coefficients, respectively, and $n_3(s,t)$ is the axial component of torque density $\mathbf{n}$.

To create shear flow, we impose a linear shear profile in the y-direction, with the shear velocity field defined as

$$\mathbf{u}_{\mathrm{shear}}(\mathbf{x}) = (0, \gamma z, 0), \tag{13}$$

where $\mathbf{x} = (x,y,z)$ and γ denotes the shear rate. The corresponding angular velocity field associated with this shear flow is given by

$$\mathbf{w}_{\mathrm{shear}}(\mathbf{x}) = \frac{1}{2}\nabla\times\mathbf{u}_{\mathrm{shear}} = (-\frac{\gamma}{2}, 0, 0). \tag{14}$$

Both the translational velocity $\mathbf{u}_{\mathrm{shear}}(\mathbf{x})$ and the angular velocity $\mathbf{w}_{\mathrm{shear}}(\mathbf{x})$ are added directly to the velocities of the immersed cell as specified in Eq. 12.

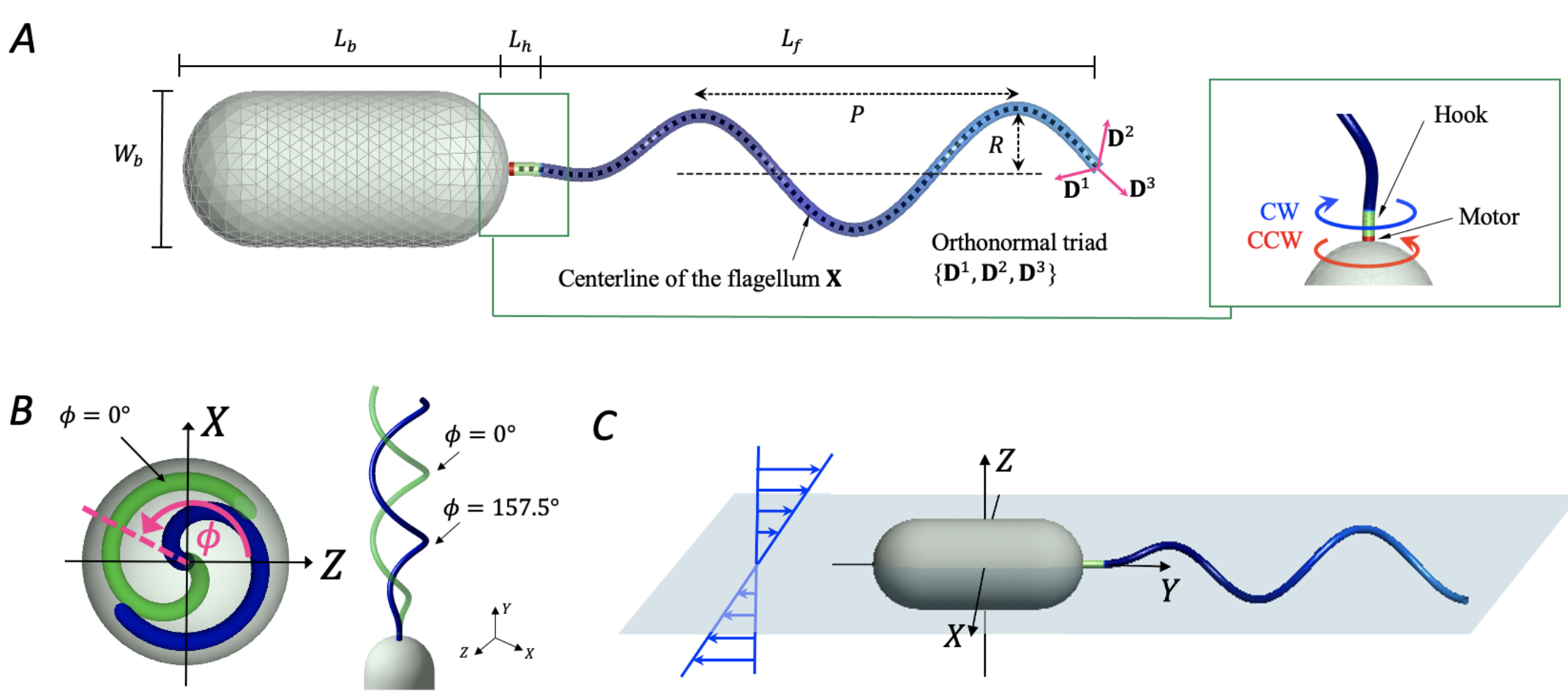

Figure S2: (A) Schematic diagram of the numerical model organism. (B) Definition of the initial phase angle (ϕ) of the flagellum. In the left panel, the initial phase angle ϕ is defined by counterclockwise rotation of the $\phi = 0°$ flagellum (green) about the helical axis. Flagella with initial phase angles of 0° (green) and 157.5° (navy) are shown in the right panel. (C) Initial setup under shear flow. The dark blue arrows indicate the direction and magnitude of the flow. The light blue panel represents the imaginary focal plane, where the fluid velocity is zero, corresponding to the experimental observation field. In the numerical simulation, flow velocities are computed by subtracting the experimental flow velocity from the measured velocity in the observation field.

Parameter (symbol)	Value
Mesh width for flagellum (Δs)	$0.0303\,\mu m$
Helical radius of filament (R)	$0.35\,\mu m$
Helical pitch of filament (P)	$2.0\,\mu m$
Axial length of helical filament (L_f)	$3.5\,\mu m$
Curvilinear length of filament (ℓ)	$4.99\,\mu m$
Intrinsic curvature of filament ($\kappa_1 = \kappa_2$)	$1.5637\,\mu m^{-1}$
Intrinsic twist of filament (κ_3)	$1.4222\,\mu m^{-1}$
Bending modulus of filament ($a_1 = a_2 = a$)	$0.003\,g\mu m^3 s^{-2}$
Twist modulus of filament (a_3)	$0.003\,g\mu m^3 s^{-2}$
Shear modulus ($b_1 = b_2$)	$2\,g\mu m s^{-2}$
Stretch modulus (b_3)	$2\,g\mu m s^{-2}$
Length of hook (L_h)	$4\Delta s$
Bending modulus of hook ($a_{1,2}^{hook} = a^{hook} = a/25$)	$0.0012\,g\mu m^3 s^{-2}$
Twist modulus of hook ($a_3^{hook} = a_3$)	$0.003\,g\mu m^3 s^{-2}$
Torque (τ)	2.0 (push), -2.0 (pull), -4.0 (wrapped) nN·nm
Cell body length (L_b)	$2.2\,\mu m$
Cell body width (W_b)	$0.9\,\mu m$
Penalty parameter for restoring force (K)	$2.0\,gs^{-2}$
Number of material points on the cell body (n_b)	770
Fluid viscosity (μ)	$1 \times 10^{-4}\,g(\mu ms)^{-1}$
Regularization parameter (ε)	$3\Delta s$
Translational drag coefficient (α_1)	$1.3221 \times 10^{-4}\,gs^{-1}$
Translational drag coefficient (α_2)	$4.0 \times 10^{-6}\,g\mu ms^{-1}$
Rotational drag coefficient (β)	$1.3221 \times 10^{-4}\,gs^{-1}$
Shear rate (γ)	$0 \sim 100\ s^{-1}$
Time step (Δt)	2×10^{-8} s

Table S2: Computational and physical parameters

3 Supplemental experimental results

In this part, we discuss how the orientation of bacterial cells is defined with respect to fluid flow streamlines. Also, we consider the role of motility in the orientation and show the reorientation process of the individual swimming cells.

3.1 Swimmers' velocities and run time

To demonstrate that swimming bacteria do not change their motility in response to local shear, the swimming speeds are measured under flow conditions and in the rest fluid. Velocities are comparable for cell populations at the same distance above the channel surface.

conditions	run time, s		
	push	pull	wrapped
bulk:			
no flow	0.30	0.39	0.56
flow	0.34	0.43	0.54
surface:			
no flow	0.39	0.25	0.34
flow	0.31	0.62	0.49

Table S3: The mean run time in different swimming modes. By italic font, the measurement focal area is specified, where *bulk* is related to $h = 20\,\mu\text{m}$ and *surface* to $h = 5\,\mu\text{m}$ above the bottom surface of the channel.

conditions	swimming velocity, $\mu m/s$		
	push	pull	wrapped
no flow	25.8	16.6	17.2
flow	23.2	17.0	13.4

Table S4: The mean population velocities in different swimming modes, $h = 20\,\mu\text{m}$.

3.2 Cell body orientations and velocity vectors

We define two types of bacterial cell orientation angles: ψ corresponds to the angle between the pointing vector (vector pointed from the center of mass of the flagella to that of the cell body) and the direction of flow (drift velocity). On the other hand, θ corresponds to the angle between the direction of the displacement (by connecting the cell body positions from the previous frame to the current frame) and the direction of flow, see also Figure 1D, in the Main Text. The ψ angles are defined for push and pull modes.

The angles as a function of time for several typical swimmers are shown in Figure S3. As expected, for the push configuration, the values of the ψ and θ angles are almost the same, the cell body is oriented similarly to the direction of the swimming motion. The difference between the direction of motion and the cell body orientation is around 180° for the pull configuration.

Throughout the cell population, the cell displacement direction (ψ angle) can be assigned with cell body orientation (θ angle) with respect to flow. However, for individual tracks, the time evolution of the angles might be slightly different, see Figure S3. For the wrapped swimming mode, the ψ angle is not defined, and we discuss only the θ angle in the Main Text.

3.3 Dynamics of cell reorientation

To show the details of the cells' reorientation process, the θ angles with time are considered for the long swimming episodes, see Figure S4. The distance between single dots corresponds to the velocities of the

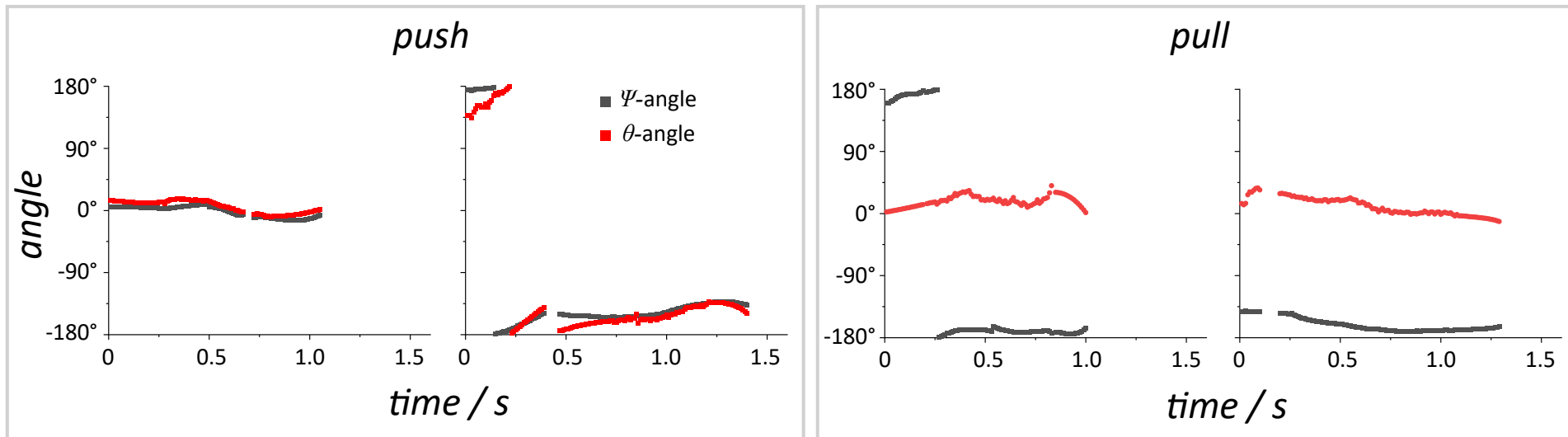

Figure S3: Typical examples of the cells orientation with time. Cell body orientations(ψ angles) and direction of forward swimming (θ angles) with respect to flow, shear rate $1.5s^{-1}$, $h = 20\,\mu\text{m}$. The gaps in the tracking happen due to the loss of one of the signals (red or green) of the swimmer.

angular changes. For the wrapped configuration, the angular velocities are higher in comparison with the unwrapped modes (see Figure S4, heatmaps) and the amplitude of the angular changes along the tracks are bigger (see green and red dots in plots). For the unwrapped modes, the reorientations around 180° and -180° are slower across streamline orientations. The reorientation of the swimming locomotion direction is not persistent. The competition between the alignment and chirality-driven drift-side drift can cause it at the same time as thermal noise.

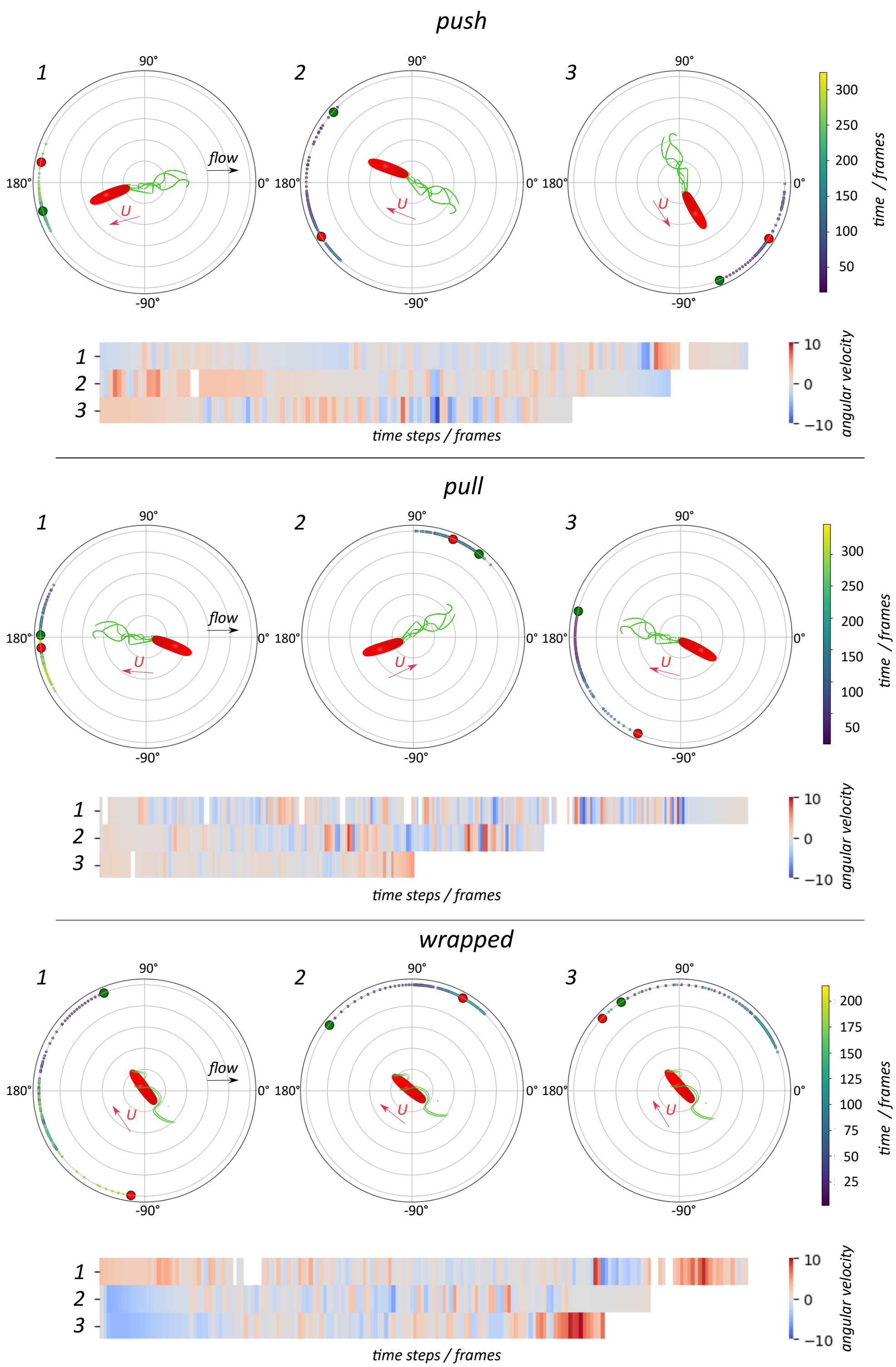

Figure S4: Orientations of velocity vectors (ψ angles) and angular velocities of individual cells in different swimming modes. Results are shown for the typical long episodes in bulk. Colorbar representing the time, time resolution 100 fps, the green dots indicate the angle at the beginning of the episode, the red dots point to the end of it. Heatmaps were shown for the filtered angular velocities between +10° and -10°. Shear rate $1.5s^{-1}$, $h = 20\,\mu m$.

4 Statistical analysis: Bayesian inference of bacterial alignment

4.1 Parametric inference—maximum-likelihood approach

In Figures. 3, 4B and 5B of the main text, histograms of the cell body orientation (angle ψ) and the swimming direction of bacteria with respect to the flow (angle θ) are shown, respectively[1]. Due to the high persistence of bacterial motion, consecutive orientation vectors during one run episode (push, pull or wrapped) are highly correlated. For small numbers of trajectories, this may introduce unintended biases or statistical artfacts in the histograms, e.g. due to different trajectory lengths. To verify the robustness of the reported results, we present a complementary statistical analysis in this appendix section.

In order to avoid the usage of correlated angle data, we select only one angle φ from each run episode at random; in the following, the variable φ may stand for both, the body orientation ψ or the displacement direction θ, depending on the context. This random selection procedure leads to a reduction of the sample size, such that histograms are not immediately informative. Here, we therefore rather employ a parametric inference approach that does not rely on binned histograms.

As bacteria are expected to align their body axis with respect to the flow [8], we model the distribution of angles $P(\varphi)$ as a mixture of a uniform background and two Gaussian peaks located at 0 and π,

$$P(\varphi|\sigma_1, \sigma_2, p_1, p_2) = p_1 G(\varphi|0, \sigma_1) + p_2 G(\varphi|\pi, \sigma_2) + \frac{1 - p_1 - p_2}{2\pi}, \tag{15}$$

in which

$$G(\varphi|\mu, \sigma) = \frac{1}{2\pi\sigma^2} \sum_{k=-\infty}^{\infty} \exp\left[-\frac{(\varphi - \mu + 2\pi k)^2}{2\sigma^2} \right] \tag{16}$$

abbreviates the probability density function (PDF) of a wrapped Gaussian distribution with location parameter μ and width σ. The parameters p_i determine the relative weight of the two Gaussians with respect to the uniform background. Note that more subtle effects, such as shifts in the peak positions or an asymmetry of the peaks are not taken into account in this analysis, where we focus solely on the overall shear-induced alignment.

Given a sample of N uncorrelated angles φ_j, the likelihood function $L_{\boldsymbol{\varphi}}(\sigma_1, \sigma_2, p_1, p_2)$ is obtained as the product of the probability densities $P(\varphi|\sigma_1, \sigma_2, p_1, p_2)$, evaluated at φ_j:

$$L_{\boldsymbol{\varphi}}(\sigma_1, \sigma_2, p_1, p_2) = \prod_{j=1}^{N} P(\varphi_j|\sigma_1, \sigma_2, p_1, p_2). \tag{17}$$

We inferred the parameter values of the statistical model Eq. (15) by maximizing the likelihood with respect to the parameter values. Figures S5-S7 show the inferred distributions along with the empirical histograms of the underlying sample (for illustration purposes only).

4.2 Quantification of bacterial alignment

Supplemented by suitable priors, the likelihood-based approach outlined above enables us to calculate Bayesian uncertainty bounds. Here, we use uniform priors for the parameters,

$$p(\sigma_1, \sigma_2, p_1, p_2) \propto \Theta(\sigma_1)\,\Theta(\sigma_2)\,\Theta(\pi - \sigma_1)\,\Theta(\pi - \sigma_2)\,\Theta(p_1)\,\Theta(p_2)\,\Theta(1 - p_1 - p_2)\,, \tag{18}$$

in which $\Theta(x)$ denotes the Heaviside-step function. By virtue of Bayes' theorem, the product of likelihood and prior yields the posterior distribution of parameters

$$P(\sigma_1, \sigma_2, p_1, p_2|\varphi_1, \varphi_2, \ldots, \varphi_N) \propto L_{\boldsymbol{\varphi}}(\sigma_1, \sigma_2, p_1, p_2) p(\sigma_1, \sigma_2, p_1, p_2) \tag{19}$$

that describes the degree of believe of parameters $\{\sigma_1, \sigma_2, p_1, p_2\}$ given the observed data $\{\varphi_1, \varphi_2, \ldots, \varphi_N\}$. It therefore allows to determine uncertainty bounds of parameter values.

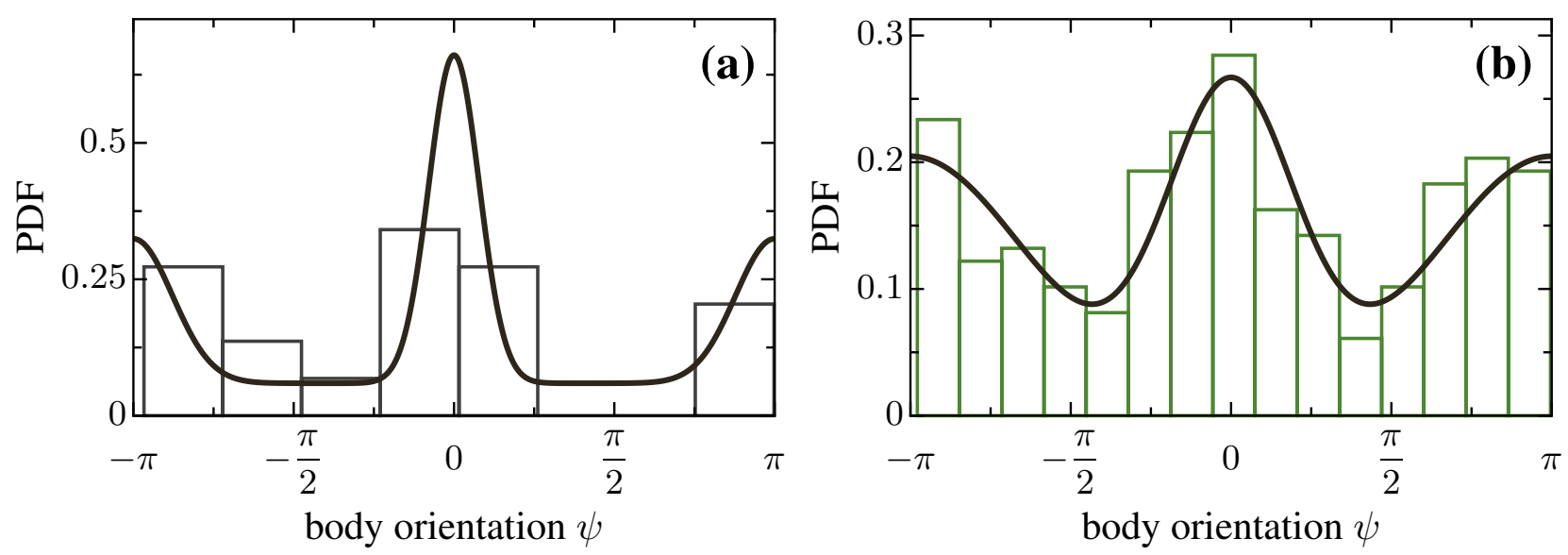

Figure S5: PDF of the body orientation ψ, inferred by likelihood maximization (black line), of (a) non-motile (passive) vs. (b) actively moving bacteria, along with the histograms of the underlying sample (see also Figure 3 of the main text). Sample size: $N = 19$ (passive), $N = 238$ (active).

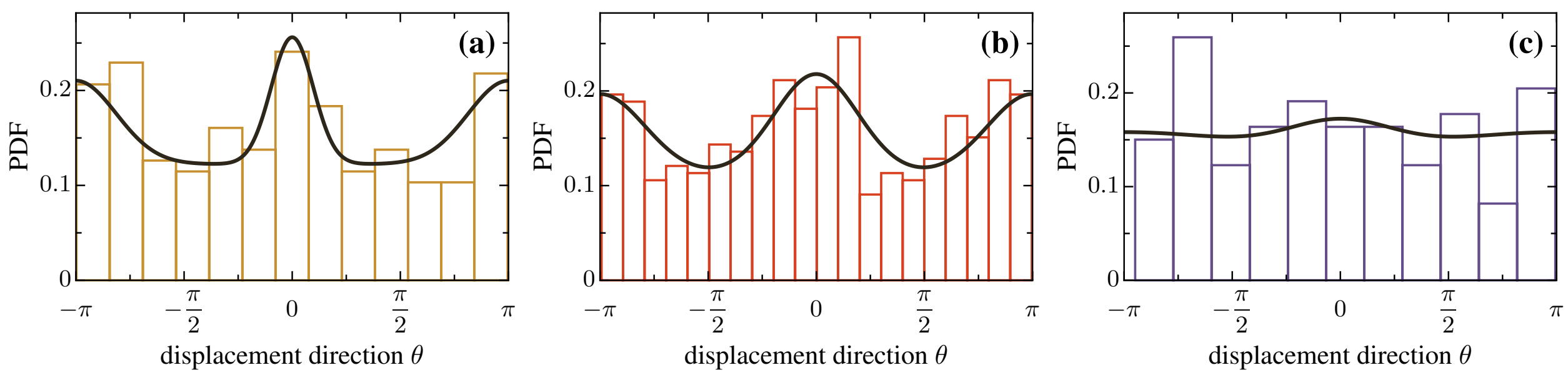

Figure S6: PDF of the displacement orientation θ, inferred by likelihood maximization (black line), for different flow conditions (corresponding to Figure 4 of the main text), along with histograms of the underlying sample. (a) bulk flow ($h = 20\,\mu\text{m}$) with shear rate $\gamma_1 = 1.5\,\text{s}^{-1}$, (b) bulk flow ($h = 20\,\mu\text{m}$) with shear rate $\gamma_1 = 3\,\text{s}^{-1}$, (c) data taken close to a surface ($h = 5\,\mu\text{m}$) with shear rate γ_1. Sample size: $N = 181$ (a), $N = 424$ (b), $N = 132$ (c).

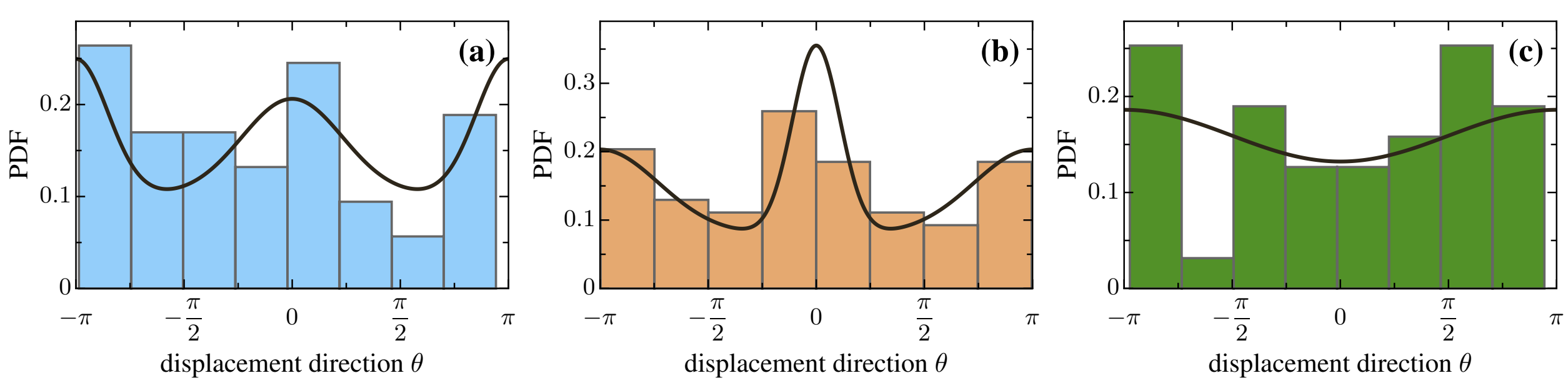

Figure S7: PDF of the displacement orientation θ, inferred by likelihood maximization (black line), for different swim modes corresponding to Figure 5 of the main text (a: push, b: pull, c: wrapped), along with histograms of the underlying sample. Data taken $h = 20\,\mu\text{m}$ above the surface at a shear rate of $\gamma_1 = 1.5\,\text{s}^{-1}$. Sample size: $N = 70$ (a: push), $N = 69$ (b: pull), $N = 42$ (c: wrapped).

In the present context, we are not interested in the parameter values themselves but rather in the relative strength of bacterial alignment under different flow conditions and of different swim modes, respectively—strong alignment is reflected by a strongly peaked distribution, whereas weak alignment results in a uniform PDF of φ angles. In order to quantify how strongly an inferred distribution deviates from the uniform

[1]The swimming direction θ was obtained from the velocity of bacteria after subtraction of the fluid velocity; the angle θ is a proxy for the direction of self-propulsion and, thus, for the body axis orientation of bacteria.

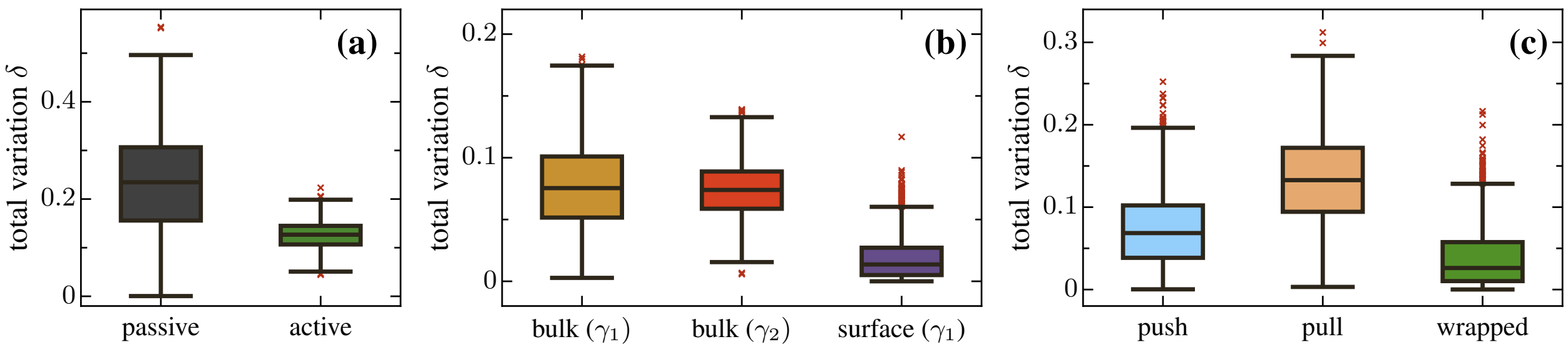

Figure S8: The box plots show the total variation δ, cf. Eq. (20), quantifying the deviation of an inferred distribution from a uniform distribution ($\delta = 0$ corresponds to the uniform distribution, $\delta = 1$ to an infinitely sharply peaked PDF). The sample of δ-values was obtained by sampling from the posterior PDF $P(\sigma_1, \sigma_2, p_1, p_2|\varphi_1, \varphi_2, \ldots, \varphi_N)$ Eq. (19). Sample size in all cases: $N = 10^3$. The data in panel (a) correspond to the data in Figure S5, (b) corresponds to Figure S6 and (c) corresponds to Figure S7.

distribution, we use the total deviation

$$\delta(\sigma_1, \sigma_2, p_1, p_2) = \frac{1}{2}\left|\int_{-\pi}^{\pi} d\varphi\, P(\varphi|\sigma_1, \sigma_2, p_1, p_2) - \frac{1}{2\pi}\right|. \tag{20}$$

It is generally used as a measure of similarity of probability distributions: the total variation δ equals zero if the inferred distribution $P(\varphi|\sigma_1, \sigma_2, p_1, p_2)$ is equal to the uniform density, and one for infinitely sharp peaks ($\sigma_{1,2} \to 0$).

We sampled 10^3 parameter combinations from the posterior $P(\sigma_1, \sigma_2, p_1, p_2|\varphi_1, \varphi_2, \ldots, \varphi_N)$, Eq. (19), applying Metropolis–Hastings algorithm, and calculated the total variation each time. The resulting distributions of δ-values are shown as box plots in Figure S8. In Figure S8a, the alignment of non-motile bacteria are compared to actively moving bacteria (cf. Figure 3 of the main text); the alignment in the latter case is reduced due to changes of swim modes which are accompanied by a randomization of the direction of motion. Figure S8b reveals that bacterial alignment is significantly reduced close to surfaces in comparison to the alignment in bulk at comparable shear rates (cf. Figure 4B of the main text). In addition, Figure S8c shows that bacteria in the wrapped swimming mode tend to be less aligned as compared to bacteria in the push and pull mode (see also Figure 5B of the main text).

5 Comparison of simulation results with the experiment

In this part, we numerically reproduced the experimental orientation histograms displayed in Figure 5 of the main text. Specifically, we simulated larger numbers of cells swimming in push, pull, and wrapped mode (28 cells in each mode) in the shear flow, starting from different initial body orientations and with different initial flagella phases. From this new data set, we computed directional histograms, similar to the experimental ones shown in Figure 5, and could successfully reproduce the different rheotactic responses of pushers and pullers. The direction of the y-axis in Figure S9 corresponds to the flow direction.

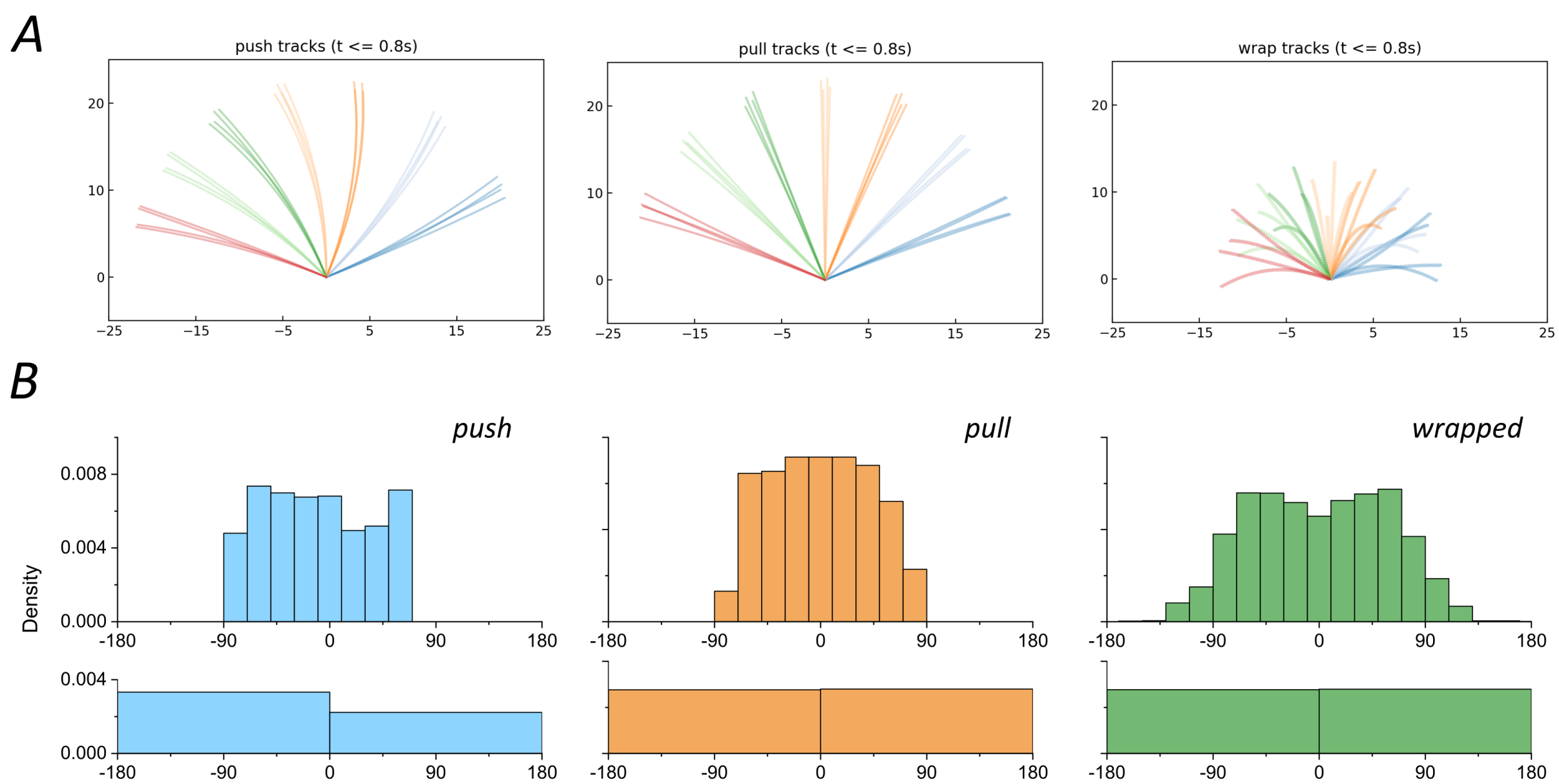

Figure S9: Numerical simulations: trajectories of bacteria in a shear flow. Trajectories of swimmers in push, pull, and wrapped modes with different initial body orientations under shear flow conditions. (A) Top views are shown for each swimming mode. In each view, 28 trajectories, corresponding to 7 different initial cell body orientations and 4 different initial flagella phases, are displayed for each swimming mode. Each color represents a different body tilting angle ($22.5° \times 0, \pm1, \pm2, \pm3$) with respect to the flow direction. Different trajectories with the same color correspond to different initial flagellar phase angles ($\phi = 0°, 90°, 180°, 270°$). Each simulation is run for 0.8 s with a shear rate of $\gamma = 3.0\ s^{-1}$. The torque values for each mode are as follows: $\tau = 2.0$ nN·nm for push, $\tau = -2.0$ nN·nm for pull, and $\tau = -4.0$ nN·nm for the wrapped mode. (B) Corresponding distributions of θ angles, separated according to swimming modes, the time resolution of the trajectories used to compute the histograms is $\Delta T = 0.05$ s.

References

[1] V. Pfeifer, V. Muraveva, and C. Beta, Flagella and Cell Body Staining of Bacteria with Fluorescent Dyes, Cell Motility and Chemotaxis: Methods and Protocols, 79-85, (2024)

[2] M. Hintsche, V. Waljor, R. Großmann, M.J. Kühn, K.M. Thormann, F. Peruani, and C. Beta, A polar bundle of flagella can drive bacterial swimming by pushing, pulling, or coiling around the cell body, Scientific Reports 7, 16771 (2017)

[3] A. Datta, TrackScoPy: A Python toolbox for tracking and analyzing bacterial motility from fluorescence microscopy videos, Zenodo [doi:10.5281/zenodo.17456571] (2025)

[4] M. Theves, J. Taktikos, V. Zaburdaev, H. Stark, and C. Beta, A Bacterial Swimmer with Two Alternating Speeds of Propagation, Biophysical Journal, 105(8), 1915 (2013)

[5] J. Crocker and D. Grier, Methods of Digital Video Microscopy for Colloidal Studies, Journal of Colloid and Interface Science, 179(1), 289 (1996)

[6] S. Olson, S. Lim, and R. Cortez, Modeling the dynamics of an elastic rod with intrinsic curvature and twist using a regularized Stokes formulation, Journal of Computational Physics, 238, 169 (2013)

[7] W. Lee, Y. Kim, C. S. Peskin, and S. Lim, A novel computational approach to simulate microswimmers propelled by bacterial flagella, Physics of Fluids 33, 111903 (2021)

[8] G. Jing, A. Zöttl, É. Clement, and A. Lindner, Chirality-induced bacterial rheotaxis in bulk shear flows, Science Advances 6, eabb2012 (2020)
